# Synthesis and Bioevaluation of Chalcones as Broad-Spectrum Antiviral Compounds Against Single-Stranded RNA Viruses

**DOI:** 10.3390/biom15091285

**Published:** 2025-09-05

**Authors:** Lorael K. M. Kirton, Nasser N. Yousef, Griffith D. Parks, Otto Phanstiel

**Affiliations:** 1 College of Medicine, University of Central Florida, 12722 Research Parkway, Orlando, FL 32826, USA; lorael.kirton@ucf.edu; 2 Burnett School of Biomedical Sciences, College of Medicine, University of Central Florida, 6900 Lake Nona Blvd, Orlando, FL 32827, USA; nasser.yousef@ucf.edu (N.N.Y.); griffith.parks@ucf.edu (G.D.P.)

**Keywords:** chalcones, RNA viruses, PIV5, La Crosse, Zika, coronavirus, Hs27 cells

## Abstract

Chalcones are flavonoid compounds containing an α,β-unsaturated ketone core that are often found in plants and have diverse biological activities including antiviral activity. For example, chalcone **8o** was previously shown to have antiviral activity against human cytomegalovirus (HCMV) and human immunodeficiency virus (HIV); two viruses that use a nuclear phase to complete their growth cycle. Here, we synthesized ten new derivatives of **8o** and tested them for antiviral activity against four RNA viruses that replicate exclusively in the cytoplasm, including prototype members of the paramyxovirus, flavivirus, bunyavirus, and coronavirus families. For example, chalcones **8o** and **8p** showed potent inhibition of PIV5 replication with minimal cytotoxicity in human fibroblast cultures. Time-of-addition studies showed that these chalcones inhibit an early stage of viral replication and prevent viral spread through cell cultures. Most importantly, our top performing chalcones showed potent in vitro antiviral activity against Zika virus, La Crosse Virus, and the coronavirus OC43. These studies offer mechanistic insight into chalcone-mediated inhibition of viral replication, demonstrate the influence of functional group changes of chalcone scaffolds on their efficacy as antivirals, and support the development of chalcones as broad-spectrum antiviral compounds.

## 1. Introduction

RNA viruses present a persistent and evolving threat to global public health. This was clearly exemplified by the recent devastating effects of the SARS-CoV-2 pandemic, causing greater than 700 million infections and 7 million deaths worldwide [1]. The Center for Disease Control (CDC) has repeatedly reported its concern about the continuous threat of RNA viruses, specifically emphasizing viral families with low existing countermeasures and high pandemic potential [2]. Virus groups that are of particular concern for future pandemics include bunyaviruses, which are responsible for hemorrhagic fevers and encephalitis, flaviviruses such as Zika virus (ZIKV), and the large and diverse paramyxovirus family [2]. The CDC also highlights the importance of countermeasures against RNA viruses that are characterized by rapid mutation and evolution. An example of this includes the emergence of numerous strains of the coronavirus (a family of RNA viruses), presenting challenges to prevention and treatment measures. The high mutation rates, diverse transmission pathways, and propensity for emergence and re-emergence make RNA viruses a formidable threat to human health.

While antiviral drugs provide powerful countermeasures to certain RNA viruses, there are limitations. For example, most existing antivirals exhibit narrow specificity, targeting only a limited range of viral strains or families [3]. This is an inherent limitation when pandemics or endemics emerge because the development of compounds effective at targeting specific strains of a virus only occurs after the virus has been identified and has already caused significant damage. In contrast to DNA viruses, most RNA viruses replicate exclusively in the cytoplasm of infected cells and use unique RNA-dependent-RNA polymerases for the amplification of their genomes. Importantly, the rapid mutation rates of RNA viruses can lead to the development of resistance to a given antiviral compound, necessitating the continuous development of new antiviral compounds [4]. These very real limitations of current antivirals underscore the need for ongoing research and development into broad-spectrum antivirals that could be used to respond to future RNA virus outbreaks.

**Figure 1:**
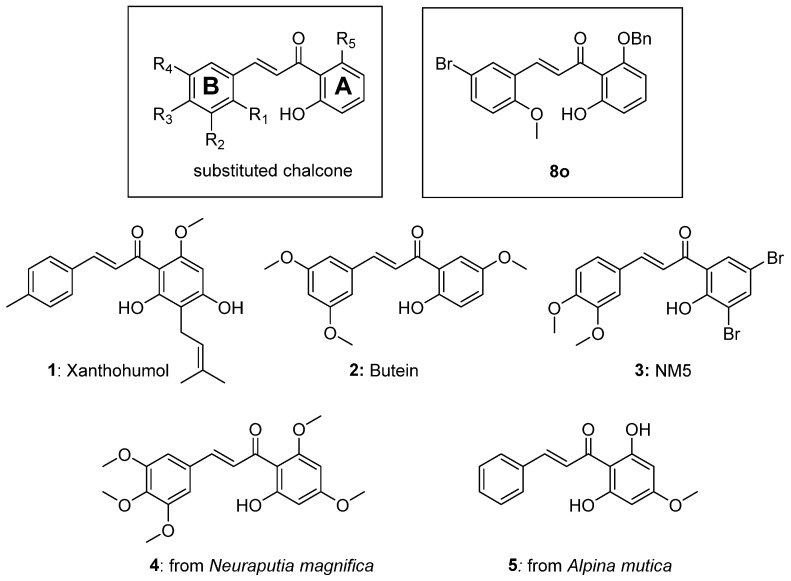
Chalcone scaffold with delineated **A** and **B** rings (left box), compound **8o** (right box), bioactive chalcone structures **1**–**3**, and related natural products **4**–**5.** Note: Xanthohumol **1** (found in hops) and butein **2** have anti-inflammatory properties [8,9], and NM5 **3** has been suggested to inhibit the mTOR pathway [6].

Chalcones are common precursors to flavones and flavonoid structures and have diverse biological effects [5]. For example, chalcones are known to inhibit key enzymes such as 15-hydroxyprostaglandin dehydrogenase, 5-lipoxygenase, cyclooxygenase, and protein tyrosine phosphatase 1B [5]. Chalcones were also reported to inhibit the phosphorylation of proteins involved in cell signaling cascades such as mammalian target of rapamycin (mTOR) [6]. In addition, chalcones can serve in the treatment of asthma, inflammation, type 2 diabetes, and obesity [7]. Not surprisingly, with such diverse medical applications, this propenone scaffold has received strong interest in the literature (Figure 1) [5].

Chalcones have also served as antiviral agents [7,10]. Prior work in our lab demonstrated that compound **8o** had efficacy as an antiviral agent against both human cytomegalovirus (HCMV, a double stranded DNA virus) [7] and human immunodeficiency virus (HIV, a positive sense, single-strand RNA virus) [10].

Here, we tested the antiviral potential of chalcones against prototypic members of four diverse RNA virus families, with some members of each virus family being identified as potential candidates for new emerging pathogens. Briefly described, parainfluenza virus 5 (PIV5) is a prototype member of the paramyxovirus family of viruses, some of which are important animal and human pathogens, such as mumps virus, measles virus, respiratory syncytial virus, and Nipah virus [11]. The negative-sense viral RNA genome is tightly bound into a ribo-nucleocapsid structure, which is enveloped by a lipid bilayer derived from the host cell. After binding to the cell surface, virions fuse with the plasma membrane to deposit the nucleocapsid into the cytoplasm. Similarly, bunyavirus La Crosse virus (LACV) also contains a negative sense RNA genome that is encapsidated by a lipid envelop, but here, the genome consists of three separate RNA segments, and infection of the host cell is initiated by the internalization of virus particles into cellular endosomes. LACV and other members of the bunyavirus family are important human pathogens, which are transmitted by insect bites [12]. OC43 is a prototypic member of the coronavirus family of human pathogens responsible for a large number of seasonal respiratory tract infections [13]. This enveloped positive stand RNA virus initiates infections by binding to the plasma membrane and the internalization of virions. Lastly, as member of the *Flaviviridae* family, Zika Virus (ZIKV) has emerged in recent years as a public health concern due to its broad tropism for different organs and spread by mosquitoes to new regions [14]. This enveloped positive-strand RNA virus initiates infections after binding to a range of potential receptors and internalization via clathrin-coated pits.

In search of general treatments for single-stranded RNA viruses, we investigated the antiviral activity of chalcones against PIV5, LACV, [15] OC43, and ZIKV. We also evaluated mammalian target of rapamycin (mTOR) as a possible chalcone target. Our chalcone library was predicated upon the lead compound **8o** and was synthesized through published methods using crossed aldol condensation and provided good yields of the target structures [7,10]. The modular synthetic approach allowed us to vary substituents on both the A and B rings (Figure 1) and expand the earlier structure–activity relationships (SAR) [7,10].

In this report, we show that chalcone treatment reduced the spread of PIV5 in fibroblast cell cultures when added to cultures as late as 24 h post-infection. The addition of select chalcones to cell cultures of PIV5-infected human fibroblasts resulted in a dose-dependent inhibition of PIV5 replication. Most importantly, we also demonstrate the inhibitory effects of chalcones in fibroblast cell cultures of the Bunyavirus La Crosse Virus (LACV, a negative sense single stranded RNA virus), as well as two other positive sense, single-stranded RNA viruses: the Coronavirus OC43 and the Flavivirus Zika Virus (ZIKV). These findings indicate the potential for broad spectrum anti-viral activity via specific chalcone designs. Collectively, these data provide a strong rationale for the further development of chalcone derivatives as potent, broad-spectrum antiviral treatments against diverse families of RNA viruses.

## 2. Materials and Methods

### 2.1. Cells Lines

Cultures of CHO-K1 (ATCC, catalog #CCL-61, Manassas, VA, USA) were grown at 37 °C in a humidified 5% CO_2_ atmosphere in Roswell Park Memorial Institute (RPMI 1640) medium in the presence of 10% fetal bovine serum (FBS) and 1% penicillin/streptomycin. Cultures of Hs27 (ATCC, catalog #CRL-1634) and Vero cells (ATCC, catalog #CCL-81) were grown at 37 °C in a humidified 5% CO_2_ atmosphere in Dulbecco Modified Eagle Medium (DMEM, catalog #11965118, Thermo Fisher Scientific, Waltham, MA, USA) supplemented with 10% heat-inactivated fetal bovine serum (HI FBS, Gibco, Thermo Fisher Scientific, Waltham, MA, USA). Cultures of PANC-1 (ATCC, catalog #CRL-1469) cells were grown at 37 °C in a humidified 5% CO_2_ atmosphere in DMEM with the addition of 10% fetal bovine serum and 1% penicillin/streptomycin.

### 2.2. Viruses and Infections

Parainfluenza virus 5 (PIV5) expressing green fluorescence protein (GFP) was derived from cDNA, grown at 37 °C in Madin–Darby bovine kidney (MDBK) cells and titered on CV-1 (African green monkey kidney) cells with a fibroblast morphology as previously described [16]. MDBK and CV-1 cells were the kind gift of Dr. Robert Lamb (Northwestern University). Cells were infected with virus diluted in DMEM supplemented with 10% bovine serum albumin (BSA) for 1 h or mock infected with media alone. Following incubation, cells were washed with phosphate-buffered saline (PBS) and cultured in DMEM supplemented with 2% heat-inactivated (HI) FBS.

La Crosse Virus (LACV) was kindly provided by Andrew Pekosz (Johns Hopkins Bloomberg School of Public Health, Baltimore, MD, USA), grown at 28 °C in C6/36 cells derived from the larvae of the Asian tiger mosquito (ATCC), and titered at 37 °C on Vero cells as previously described [17]. Infections were carried out in DMEM containing 2% HI FBS for 1 h or mock infected with media alone. Cells were washed with PBS and cultured in DMEM containing 2% HI FBS.

The MR766 strain of Zika Virus (ZIKV) (ATCC-VR84, Manassas, VA, USA) was grown and titered at 37 °C using Vero cells as previously described [18]. Infections were carried out in DMEM containing 10% BSA for 1 h. Cells were then washed with PBS and cultured in DMEM containing 10% HI FBS.

Human Coronavirus OC43 (ATCC, catalog number VR-1558) was grown in a human colorectal adenocarcinoma cell line (HCT-8; ATCC, Manassas, Virginia, catalog #CCL-244) at 33 °C as previously described [19]. OC43 titers were determined at 33 °C via 50% Tissue Culture Infectious Dose assays (TCID_50_) on confluent human rhabdomyosarcoma (RD; ATCC, Manassas, Virginia, catalog # CCL-136) cells in 96-well plates as previously described [19]. Infections were carried out in DMEM containing 10% BSA for 1 h at 33 °C. Cells were then washed with PBS, cultured in DMEM supplemented with 2% HI FBS, and incubated at 33 °C.

The starting titers of stocks of PIV5, OC43, ZIKV, and LACV were 5.6 × 10^7^ PFU/mL, 2 × 10^7^ TCID_50_/mL, 1 × 10^6^ PFU/mL, and 1.1 × 10^7^ PFU/mL, respectively. Unless otherwise stated for a particular experiment, all high and low MOI virus infections were performed at 10 and 0.01 infectious units per cell, respectively.

### 2.3. Chalcone Treatments

Chalcone treatments were performed in DMEM supplemented with 10% HI FBS at the indicated concentrations and for indicated periods of time. Chalcone stocks were reconstituted in 100% dimethyl sulfoxide (DMSO) at a concentration of 10 mM and were serially diluted to working concentrations in DMSO. DMSO control treatments to test the effect of the vehicle were performed at volumes corresponding to the volume of chalcone added during each experiment (i.e., 1 µL chalcone DMSO solution/1 mL DMEM [10 µM] utilized alongside the DMSO control of 1 µL/1 mL DMEM). For cytotoxicity assays, untreated controls were subtracted from DMSO and chalcone sample values to account for the background signal.

### 2.4. Flow Cytometry

For cytotoxicity assays, cells were stained with propidium iodide (PI) at a dilution of 1:100. Cell viability was determined via flow cytometry using the CytoFLEX (Beckman Coulter, Brea, CA, USA). CytExpert software (Beckman Coulter, version 2.4) was used to analyze independent events. PIV5 infection was monitored using flow cytometry via the FITC channel by analyzing GFP expression of individual cells. ZIKV infection was monitored utilizing Flavivirus group antigen–antibody (D1-4G2-4-15; Novus Biologicals, Littleton, CO, USA) staining for the ZIKV envelope protein. Cells were fixed and permeabilized using eBioscience Intracellular Fixation and Permeabilization Buffer (Invitrogen, Thermo Fisher Scientific, MA, USA) according to the manufacturer’s instructions. Secondary staining was performed using an anti-Alexa Fluor 488 (AF488) antibody (Invitrogen, Thermo Fisher Scientific, MA, USA). OC43 infection was monitored utilizing a primary antibody targeted toward the OC43 nucleocapsid protein (NP, MAB9013, Sigma-Aldrich, St. Louis, MO, USA). Secondary staining was performed using an AF488 antibody. Cells were fixed and permeabilized as previously described. LACV infection was monitored utilizing an anti-LACV Gc 807.31ab antibody kindly provided by Andrew Pekosv. Secondary staining was performed using an AF488 antibody. Cells were fixed and permeabilized as previously described [19].

### 2.5. RT-qPCR

Hs27 cells cultured in 6-well dishes were collected in TRIzol^®^ (Invitrogen) followed by RNA extraction. To produce cDNA, 1 μg of total RNA was used with TaqMan^®^ Reverse Transcription Reagents (Applied Biosystems, Foster City, CA, USA) as described in the manufacturer’s instructions. Quantitative real-time PCR was performed using Bio-Rad CFX Connect Real-Time (Bio-Rad, Hercules, CA, USA) and Fast SYBR^®^ FAST Green Master Mix (Applied Biosystems, Foster City, CA, USA). Relative gene expression was determined using CFX Manager Software (Bio-Rad, version 2.3) and the following primers (Table 1).

### 2.6. Western Blotting

Hs27 (human skin fibroblast) cells cultured in 6-well dishes were lysed using protein lysis buffer. Cell lysates were resolved on 12% sodium dodecyl sulfate-polyacrylamide gel electrophoresis (SDS-PAGE) gels and transferred to nitrocellulose membranes (Bio-Rad, Hercules, CA, USA). After normalizing samples via western blotting for β-actin (1:20,000 dilution, catalog number A5316, Sigma-Aldrich, St. Louis, MO, USA), samples were probed with rabbit polyclonal antibodies for the PIV5 NP (1:2000) and P proteins (1:2000) [20]. Blots were visualized using anti-mouse horseradish peroxidase (HRP)-conjugated antibodies (Sigma-Aldrich) and chemiluminescence (Thermo Fisher Scientific).

PANC-1 cells were cultured in 6-well dishes and were lysed using modified radioimmunoprecipitation assay (RIPA) buffer (20 mM HEPES, pH 7.0, 150 mM NaCl, 1 mM EDTA, 1% NP-40, 1% deoxycholate, and 0.1% sodium dodecyl sulfate [SDS]) containing a complete protease inhibitor cocktail (Roche, Mannheim, Germany) and PhosSTOP phosphatase inhibitor cocktail (Roche, Mannheim, Germany). Protein concentrations were determined using the bicinchoninic acid (BCA) protein assay kit (Pierce, Rockford, IL, USA) according to manufacturer’s instructions. Cell lysates with 25 µg of protein were resolved on 12% sodium dodecyl sulfate-polyacrylamide gel electrophoresis (SDS-PAGE) gels and electro-transferred onto polyvinylidene difluoride (PVDF) membranes (Trans-Blot Turbo Transfer System, BioRad, Hercules, CA, USA). The samples were probed with rabbit monoclonal antibodies for the P-rps6 (1:1000) protein (Cell Signaling, Danvers, MA, USA) and mouse monoclonal antibodies for rps6 (1:1000) (Cell Signaling) and β-actin (1:5000) (Abcam, Waltham, MA, USA) proteins. Blots were visualized using anti-rabbit or anti-mouse horseradish peroxidase (HRP)-conjugated antibodies (Cell Signaling) and chemiluminescence reagent (Thermo Fisher Scientific, Waltham, MA, USA).

### 2.7. Growth Inhibition Assay

A cell growth assay using the MTS reagent was conducted to assess the general toxicity of each chalcone compound. Briefly, cell growth was assayed in sterile 96-well microtiter plates (Costar 3599, Corning Inc., Corning, NY, USA) in the presence of each chalcone. Experiments were conducted in triplicate. In a typical experiment, there are two plates. The first is the control plate, which allows one to assess the starting cell population on the day of drug addition (Day 0). The second plate is the test plate where cells are plated and tested with different drug concentrations on Day 0. For example, either CHO cells or Hs27 cells were plated in a 200 µL volume at 1000 cells/well on both the control and test plates and incubated overnight to allow the cells to adhere. On Day 0, 1 μL of PBS was added to the control plate wells so that their total volume was 201 μL. MTS reagent (Promega Cell Titer 96 Aqueous non-radioactive cell proliferation reagent, Promega, Madison, WI, USA) was added (20 μL) to each well on the control plate, and that plate was incubated for 4 h in a 5% CO_2_ atmosphere at 37 °C and then read on the plate reader. This data provided the starting absorbance for each well on Day 0.

On Day 0, 1 μL of a 200× stock solution of each chalcone was added to the respected well on the test plate, and each condition was tested in triplicate. Drug solutions were prepared in advance in 100% DMSO and dosed so that the final DMSO concentration was 0.5%. For example, 1 μL of chalcone solution was added to the cells plated in each well in 200 μL of media. Of note, the respective chalcone addition occurred after an initial overnight incubation of each cell line in each well to reach Day 0 (the drugging day). After the chalcone was added, the cells were incubated in 5% CO_2_ for 48 h at 37 °C. After 48 h, the MTS reagent (Promega, Madison, WI, USA) was added (20 μL), and the test plates were incubated for an additional 4 h, and then, absorbance at 490 nm was measured on a BioTek Synergy MX plate reader. Controls run using ≤0.5% DMSO in the media showed no toxicity over the 48 h period compared to CHO or Hs27 cells grown in media only. IC_50_ values were determined from the corresponding plot of relative absorbance at 490 nm vs. the drug concentration. The data is tabulated in Table 2.

For highly colored materials like the chalcones, it is important to consider the contribution of the compound’s own absorbance to the MTS assay. To correct for the absorbance contribution of the chalcone itself, we measured each chalcone’s absorbance (Chalcone Abs) at 490 nm at the respective concentration (e.g., 10 µM) and subtracted the absorbance of the media background (blank Abs). This net value was then subtracted out of the Day 2 absorbance value to provide the % relative growth via the following equation:

(1) $$\%\;Relative\;Growth\;=\;\frac{\left(Day\;2\;Abs-Day\;2\;blank\;Abs)-\left(Chalcone\;Abs-blank\;Abs\right)-(Day\;0\;Abs-Day\;0\;blank\;Abs\right)}{\left(Day\;2\;Control\;Abs\;-Day\;2\;blank\;Abs)-(Day\;0\;Abs-Day\;0\;blank\;Abs\right)}\;\times 100\%$$

### 2.8. Statistics

Statistical analysis was performed using GraphPad’s Student’s *t*-test and ANOVA. In all figures, * indicates a *p*-value < 0.05, ** indicates a *p*-value < 0.01, *** indicates a *p*-value < 0.001, and **** indicates a *p*-value < 0.0001.

### 2.9. Synthetic Methods

Reagents were ordered from commercial sources and used without further purification. ^1^H and ^13^C NMR spectra were obtained on a Bruker AVANCE III NMR instrument at 400 MHz and 125 MHz, respectively. Proof of purity was confirmed either via Shimadzu Prominence HPLC system or elemental analyses (Atlantic Microlabs, Norcross, GA, USA). The purity of each tested compound was ≥95% pure. High-resolution mass spectrometry was performed at the University of Florida Chemistry Department as a fee for service. Condensation reactions were conducted using a water condenser containing an attached drying tube filled with CaCl_2_. Compounds **6a** and **6b** were reported previously [7,10]. For example, compound **6b** was synthesized as follows.

### 2.10. 5-Bromo-2-Ethoxybenzaldehyde (***6b***)

A mixture of 5-bromo-2-hydroxybenzaldehyde (1.07 g, 5.3 mmol), ethyl bromide (1.09 g, d = 1.46 g/mL, 745 µL, 10 mmol), and solid anhydrous potassium carbonate (1.39 g, 10 mmol) in *N*,*N*-dimethylformamide (6.7 mL) was stirred at room temperature for 1 day. TLC (15% EtOAc/hexane) was used to monitor the reaction. The workup included the evaporation of DMF under reduced pressure. The resultant oil was dissolved in dichloromethane (DCM), and the organic phase was washed with 1 M aq. HCl. The bottom organic layer was separated, dried over anhydrous Na_2_SO_4_, filtered, and evaporated under reduced pressure to obtain a light-yellow oil. The oil was heated in hexane and cooled to rt over 1 h. The product recrystallized and was collected using a Buchner funnel under a vacuum to give **6b** as a yellow solid. (0.92 g, 84%); R_f_ 0.46 (15% ethyl acetate:hexane). The ^1^H NMR matched the literature spectrum [7]. ^1^H NMR (CDCl_3_): δ 10.42 (s, 1H), 7.93 (d, 1H, *J* = 2.7 Hz), 7.61 (dd,1H, *J* = 9, 2.7 Hz), 6.88 (d, 1H, *J* = 9 Hz), 4.14 (q, 2H, *J* = 6.9 Hz), 1.48 (t, 3H, *J* = 7.1 Hz).

### 2.11. 1-[2-Hydroxy-6-(3-Methyl-Butoxy)-Phenyl]-Ethanone (***7a***)

To a solution containing 2,6-dihydroxyacetophenone (502 mg, 3.3 mmol) in acetone (5 mL), isopentyl bromide (465 mg, 394 µL, 3.0 mmol), KI (885 mg, 5.3 mmol), and solid anhydrous K_2_CO_3_ (1.46 g, 10.6 mmol) were added. The reaction mixture was heated to reflux overnight under anhydrous conditions and then cooled and filtered. The filtrate was concentrated under reduced pressure, and the residue was re-dissolved in ethyl acetate. The solution was acidified to pH 2 with 1 M HCl and washed with H_2_O (50 mL). The water layer was washed three times with ethyl acetate. The organic layers were combined, dried over anhydrous Na_2_SO_4_, filtered, and concentrated under reduced pressure to give a crude yellow oil (709 mg). The solid was purified via flash chromatography (4% ethyl acetate/hexane) on a silica gel column (60 g silica). Elution with 4% ethyl acetate and hexane gave the recovered desired product **7a** as a light-yellow oil (513 mg, 77%). R_f_ 0.34 (4% ethyl acetate/hexane). The ^1^H NMR matched the literature spectrum [21]. ^1^H NMR (CDCl_3_): δ 13.26 (s, 1H), 7.31 (t, 1H, *J* = 8.3 Hz), 6.54 (dd, 1H, *J* = 8.4, 0.6 Hz), 6.37 (d, 1H, *J* = 7.8 Hz), 4.06 (t, 2H, *J* = 6.6 Hz), 2.69 (s, 3H), 1.83 (m, 1H), 1.76 (m, 2H), 0.99 (d, 6H, *J* = 6.4 Hz). ^13^C NMR (CDCl_3_): δ 205.1, 164.7, 161.0, 136.0, 111.3, 110.4, 101.7, 67.4, 37.8, 33.8, 25.2, 22.5.

### 2.12. 1-(2-Benzyloxy-6-Hydroxy-Phenyl)-Ethanone (***7b***)

To a solution containing 2,6-dihydroxy-acetophenone (1.51 g, 9.9 mmol) in acetone (15 mL), benzyl bromide (1.54 g, 1.07 mL, 9 mmol), KI (2.64 g, 15.9 mmol), and solid anhydrous K_2_CO_3_ (4.4 g, 31.8 mmol) were added. The reaction mixture was heated to reflux overnight under an N_2_ atmosphere and then cooled and filtered. The filtrate was concentrated under reduced pressure, and the residue was re-dissolved in ethyl acetate. The solution was acidified to pH 2 with 4 M HCl and washed with H_2_O (50 mL). The water layer was washed three times with ethyl acetate. The organic layers were combined, dried over anhydrous Na_2_SO_4_, filtered, and concentrated under reduced pressure to give a crude yellow oil (2.51 g). The solid was purified via flash chromatography (10% ethyl acetate/hexane) on a silica gel column (260 g silica). Elution with 10% ethyl acetate and hexane gave the desired product **7b** as a pale-yellow oil (2.17 g, 75%). R_f_ 0.4 (10% ethyl acetate/hexane). ^1^H NMR (CDCl_3_): δ 13.24 (s, 1H), 7.43-7.31 (m, 6H), 6.59 (dd, 1H, *J* = 8.3, 0.7 Hz), 6.47 (d, 1H, *J* = 8.1 Hz), 5.13 (s, 2H), 2.62 (s, 3H). ^13^C NMR (CDCl_3_): δ 205.2, 164.7, 160.6, 136.1, 135.8, 128.8, 128.5, 128.0, 111.6, 111.0, 102.23, 71.1, 34.1.

### 2.13. 1-(2-Hydroxy-6-Phenethyloxy-Phenyl)-Ethanone (***7c***)

To a solution containing 2,6-dihydroxyacetophenone (502 mg, 3.3 mmol) in acetone (5 mL), phenethyl bromide (555 mg, 410 µL, 3.0 mmol), KI (885 mg, 5.3 mmol), and solid anhydrous K_2_CO_3_ (1.46 g, 10.6 mmol) were added. The reaction mixture was heated to reflux overnight under anhydrous conditions and then cooled and filtered. The filtrate was concentrated under reduced pressure, and the residue was re-dissolved in ethyl acetate. The solution was acidified to pH 2 with 1 M HCl and washed with H_2_O (50 mL). The water layer was washed three times with ethyl acetate. The organic layers were combined, dried over anhydrous Na_2_SO_4_, filtered, and concentrated under reduced pressure to give a crude orange solid (649 mg). The solid was purified via flash chromatography (4% ethyl acetate/hexane) on a silica gel column (52 g silica). Elution with 4% ethyl acetate and hexane gave the desired product **7c** as a pale-yellow solid (157 mg, 20%). R_f_ 0.23 (4% ethyl acetate/hexane). The ^1^H NMR matched the literature spectrum [22]. ^1^H NMR (CDCl_3_): δ 13.25 (s, 1H), 7.36 -7.23 (m, 6H), 6.56 (dd, 1H, *J* = 8.3, 1Hz), 6.38 (d, 1H, *J* = 8.3 Hz), 4.32 (t, 2H, *J* = 7 Hz), 3.19 (t, 2H, *J* = 6.8 Hz), 2.54 (s, 3H). ^13^C NMR (CDCl_3_): δ 205.2, 164.7, 160.6, 137.6, 136.0, 128.7, 128.7, 126.8, 111.3, 110.8, 101.8, 69.4, 35.5, 33.9.

### 2.14. 1-[2(Biphenyl-4-Ylmethyoxy)6-Hydroxy)-Phenyl]-Ethanone (***7d***)

To a solution containing 2,6-dihydroxyacetophenone (508 mg, 3.3 mmol) in acetone (5 mL), 4-bromomethyl-biphenyl (745 mg, 3.0 mmol), KI (888 mg, 5.3 mmol), and solid anhydrous K_2_CO_3_ (1.46 g, 10.6 mmol) were added. The reaction mixture was heated to reflux overnight under anhydrous conditions and then cooled and filtered. The filtrate was concentrated under reduced pressure, and the residue was re-dissolved in ethyl acetate. The solution was acidified to pH 2 with 4 M HCl and washed with H_2_O (50 mL). The water layer was washed three times with ethyl acetate. The organic layers were combined, dried over anhydrous Na_2_SO_4_, filtered, and concentrated under reduced pressure to give a crude orange solid (1.07 g). The solid was purified via flash chromatography (50% dichloromethane/hexane) on a silica gel column (107 g silica). Elution with (50% dichloromethane/hexane) gave the desired product **7d** as a pale-yellow solid (282 mg, 27%). R_f_ 0.29 (50% dichloromethane/hexane). ^1^H NMR (CDCl_3_): δ 13.26 (s, 1H), 7.62 (m, 4H), 7.47 (m, 4H), 7.35 (m, 2H), 6.60 (d, 1H, *J* = 8.6 Hz), 6.68 (d, 1H, *J* = 8.3 Hz) 5.16 (s, 2H), 2.64 (s, 3H). ^13^C NMR (CDCl_3_): δ 205.2, 164.8, 160.7, 141.4, 140.5, 136.1, 134.8, 128.9, 128.4, 127.6, 127.5, 127.1, 111.6, 111.1, 102.3, 70.9, 34.2. HRMS for C_21_H_18_O_3_ (M+H): theory 319.1329, found 319.1326.

Compounds **8a**, **8f**, **8l**, **8o**, and **8p** were synthesized previously [7,10].

### 2.15. (2E)-3-(5-Bromo-2-Ethoxy-Phenyl)-1-(2-Hydroxy-6-Methoxy-Phenyl)-Propenone (***8q***)

5-Bromo-2-ethoxybenzaldehyde (**6b**) (227 mg, 1 mmol), 1-(2-hydroxy-6-methoxyphenyl) ethanone (167 mg, 1 mmol), and MeOH (5 mL) were combined, and KOH in methanol (40% weight/volume, 5 mL) was added at rt and then heated to 60 °C and stirred overnight. The workup included evaporation of the solvent under reduced pressure and the dropwise addition of 12 N HCl until the solution was at pH 1. The solution was then extracted with ethyl acetate (twice). Each organic layer was separated, pooled together, dried over anhydrous Na_2_SO_4_, filtered, and concentrated to give a crude yellow oil (470 mg). Column chromatography (10% ethyl acetate:hexane, R_f_ 0.24) was performed and provided the pure product **8q** as an orange solid. (0.137 g, 36%). ^1^H NMR (CDCl_3_): δ 13.10 (s, 1H), 8.04 (d, 1H, *J* = 15.9 Hz), 7.82 (d, 1H, *J* = 15.6 Hz), 7.68 (d, 1H, *J* = 2.4 Hz), 7.39, (dd, 1H, *J* = 8.8, 2.4 Hz), 7.34 (t, 1H, *J* 8.4 Hz), 6.77 (d, 1H, *J* = 9 Hz), 6.60 (dd, 1H, *J* = 8.4, 0.9 Hz), 6.41 (d, 1H, *J* = 7.8 Hz), 4.07 (q, 2H, *J* = 6.8 Hz), 3.93 (s, 3H), 1.47 (t, 3H, *J* = 7 Hz). ^13^C NMR (CDCl_3_): δ 194.5, 164.7, 160.9, 157.0, 136.6, 135.8, 133.7, 130.9, 128.7, 126.3, 113.8, 112.7, 112.0, 110.8, 101.5, 64.3, 56.0, 14.7. HRMS for C_18_H_17_BrO_4_ (M+H): theory 377.0399, found 377.0388. mp 84–87 °C.

### 2.16. (2E)-3-(5-Bromo-2-Ethoxy-Phenyl)-1-[2-Hydroxy-6-(3-Methyl-Butoxy)-Phenyl]-Propenone (***8r***)

5-Bromo-2-ethoxybenzaldehyde (**6b**) (115 mg, 0.5 mmol) and 1-[2-Hydroxy-6-(3-methyl-butoxy)-phenyl]-ethanone (**7a**) [21] (111 mg, 0.5 mmol), and MeOH (2.5 mL) were combined, and KOH in methanol (40% weight/volume, 2.5 mL) was added at rt and then heated to 60 °C. The reaction mixture was stirred at 60 °C overnight. Workup included evaporation of the solvent under reduced pressure, dropwise addition of 4N HCl until solution was at pH 1. The solution was then extracted with ethyl acetate (thrice). Each organic layer was separated, pooled together, dried over anhydrous Na_2_SO_4_, filtered and concentrated to give a crude orange solid (212 mg). Column chromatography (6% ethyl acetate:hexane, R_f_ 0.28) was performed and provided the pure product **8r** as an orange solid. (141 mg, 71%). ^1^H NMR (CDCl_3_): δ 13.10 (s, 1H), 8.11 (d, 1H, *J* = 15.9 Hz), 7.87 (d, 1H, *J* = 15.9 Hz), 7.74 (d, 1H, *J* = 2.4 Hz), 7.42 (dd, 1H, *J* = 8.8, 2.4 Hz), 7.34 (t, 1H), 6.80 (d, 1H, *J* = 9 Hz), 6.60 (dd, 1H, *J* = 8.3, 1 Hz), 6.42 (d, 1H, *J* = 7.6 Hz), 4.09 (m, 4H), 1.79 (m, 3H), 1.48 (t, 3H, *J* = 7.1 Hz), 0.92 (m, 6H). ^13^C NMR (CDCl_3_): δ 194.6, 164.8, 160.6, 158.9, 136.0, 135.7, 133.8, 129.9, 128.8, 126.5, 114.0, 112.9, 112.1, 110.7, 102.2, 67.5, 64.5, 38.1, 25.1, 22.5, 14.7. HRMS for C_22_H_25_BrO_4_ (M+H): theory 433.1014, found 433.0990. mp 84–86 °C.

### 2.17. (2E)-3-(5-Bromo-2-Methoxy-Phenyl)-1-[2-Hydroxy-6-(3-Methyl-Butoxy)-Phenyl]-Propenone (***8s***)

5-Bromo-2-methoxybenzaldehyde (108 mg, 0.5 mmol) and 1-[2-Hydroxy-6-(3-methyl-butoxy)-phenyl]-ethanone (**7a**) [21] (112 mg, 0.5 mmol), and MeOH (2.5 mL) were combined, and KOH in methanol (40% weight/volume, 2.5 mL) was added at rt and then heated to 60 °C. The reaction mixture was stirred at 60 °C overnight. Workup included evaporation of the solvent under reduced pressure, dropwise addition of 4N HCl until solution was at pH 2. The solution was then extracted with ethyl acetate (thrice). Each organic layer was separated, pooled together, dried over anhydrous Na_2_SO_4_, filtered and concentrated to give a crude orange solid (195 mg). Column chromatography (6% ethyl acetate:hexane, R_f_ 0.23) was performed and provided the pure product **8s** as an orange solid (122 mg, 58%). ^1^H NMR (CDCl_3_): δ 13.11 (s, 1H), 8.09 (d, 1H, *J* = 15.6 Hz), 7.88 (d, 1H, *J* = 15.7 Hz), 7.73 (d, 1H, *J* = 2.4 Hz), 7.44 (dd, *J* = 8.8, 2.4 Hz) 7.34 (t, 1H, *J* = 8.3 Hz), 6.82 (d, 1H, *J* = 8.8 Hz), 6.60 (d, 1H, *J* = 8.3 Hz), 6.42 (d, 1H, *J* = 6.42 Hz), 4.10 (t, 2H, *J* = 6.4 Hz), 3.89 (s, 3H), 1.80 (m, 3H) 0.93 (d, 6H). ^13^C NMR (CDCl_3_): δ 194.6, 160.6, 157.5, 136.0, 135.7, 133.8, 130.1, 129.0, 126.4, 113.1, 113.0, 112.1, 110.7, 102.2, 77.2, 67.5, 55.9, 38.1, 25.1, 22.5. HRMS for C_21_H_23_BrO_4_ (M+H): theory 419.0689, found 419.0693. mp 94–98 °C.

### 2.18. (2E)-3-(5-Bromo-2-Ethoxy-Phenyl)-1-[2-Hydroxy-6-Phenethyloxy]-Propenone (***8t***)

5-Bromo-2-ethoxybenzaldehyde (**6b**) (47 mg, 0.2 mmol) and 1-[2-Hydroxy-6-phenethyloxy-phenyl]-ethanone (**7c**) [22] (51 mg, 0.2 mmol), and MeOH (2.5 mL) were combined, and KOH in methanol (40% weight/volume, 2.5 mL) was added at rt and then heated to 60 °C. The reaction mixture was stirred at 60 °C overnight. Workup included evaporation of the solvent under reduced pressure, dropwise addition of 4N HCl until solution was at pH 1. The solution was then extracted with ethyl acetate (thrice). Each organic layer was separated, pooled together, dried over anhydrous Na_2_SO_4_, filtered and concentrated to give a crude orange solid (101 mg). Column chromatography (10% ethyl acetate:hexane, R_f_ 0.25) was performed and provided the pure product **8t** as an orange solid. (55 mg, 59%). ^1^H NMR (CDCl_3_): δ 13.03 (s, 1H), 8.07 (d, 1H, *J* = 15.9 Hz), 7.84 (d, 1H, *J* = 15.7 Hz), 7.69 (d, 1H, 2.4 Hz), 7.41 (dd, 1H, *J* = 8.8, 2.4 Hz), 7.33 (t, 1H, *J* = 8.4 Hz), 7.23 (m, 5H), 6.79 (d, 1H, *J* = 8.8 Hz), 6.62 (dd, 1H, *J* = 8.3, 0.7 Hz), 4.31 (t, 2H, *J* = 7.2 Hz), 4.10 (m, 2H, 7.1 Hz), 3.20 (t, 2H, 7.2 Hz), 1.48 (t, 3H, 7 Hz). ^13^C NMR (CDCl_3_): δ 193.6, 163.7, 159.1, 155.9, 136.2, 135.0, 134.9, 132.8, 129.3, 127.9, 127.8, 127.6, 125.7, 125.5, 112.9, 111.9, 111.2, 109.9, 101.5, 68.7, 63.4, 34.7, 13.7. HRMS for C_25_H_23_BrO_4_ (M+H): theory 467.0858, found 467.0857. mp 105–110 °C

### 2.19. (2E)-3-(5-Bromo-2-Methoxy-Phenyl)-1-[2-Hydroxy-6-Phenethyloxy]-Propenone (***8u***)

5-Bromo-2-methoxy-benzaldehyde (38 mg, 0.18 mmol), 1-[2-hydroxy-6-phenethyloxy-phenyl]-ethanone (**7c**) [22] (46 mg, 0.18 mmol), and MeOH (2.5 mL) were combined, and KOH in methanol (40% weight/volume, 2.5 mL) was added at rt and then heated to 60 °C. The reaction mixture was stirred at 60 °C overnight. The workup included evaporation of the solvent under reduced pressure and the dropwise addition of 4 N HCl until the solution was at pH 1. The solution was then extracted with ethyl acetate (thrice). Each organic layer was separated, pooled together, dried over anhydrous Na_2_SO_4_, filtered and concentrated to give a crude orange solid (88 mg). Column chromatography (20% ethyl acetate:hexane, R_f_ 0.4) was performed and provided the pure product **8u** as an orange solid. (47 mg, 59%). ^1^H NMR (CDCl_3_): δ 13.05 (s, 1H), 8.04 (d, 1H, *J* = 15.7 Hz), 7.84 (d, 1H, *J* = 15.9 Hz), 7.67 (d, 1H, *J* = 2.4 Hz), 7.43 (dd, 1H, *J* = 8.8, 2.4 Hz), 7.32 (t, 1H, *J* = 8.3 Hz), 7.23 (m, 5H), 6.80 (d, 1H, *J* = 8.8 Hz), 6.60 (d, 1H, *J* = 8.3 Hz), 6.41 (d, 1H, *J* = 8.3 Hz), 4.31 (t, 2H, *J* = 7.3), 3.33 (s, 3H), 3.20 (t, 2H, *J* = 7.2 Hz). ^13^C NMR (CDCl_3_): δ 194.6. 164.8, 160.1, 157.6, 137.3, 136.1, 135.9, 133.8, 130.6, 129.1, 128.8, 128.7, 126.8, 126.4, 113.1, 113.0, 112.2, 111.0, 102.5, 69.8, 55.9, 35.7. HRMS for C_24_H_21_BrO_4_ (M+H): theory 453.0701, found 453.0704. mp 95–100 °C.

### 2.20. (2E)-1-[2-(Biphenyl-4-Ylmethoxy)-6-Hydroxy-Phenyl]3-(5-Bromo-2-Ethoxy-Phenyl)-Propenone (***8v***)

5-Bromo-2-ethoxybenzaldehyde (**6b**, 72 mg, 0.3 mmol), 1-[2(biphenyl-4-ylmethyoxy)6-hydroxy)-phenyl]-ethanone (**7d**, 100 mg, 0.3 mmol), and MeOH (2.5 mL) were combined, and KOH in methanol (40% weight/volume, 2.5 mL) was added at rt, and then, the reaction mixture was heated to 60 °C and stirred at 60 °C overnight. The workup included evaporation of the solvent under reduced pressure and the dropwise addition of 4 N HCl until the solution was at pH 1. The solution was then extracted with ethyl acetate (thrice). Each organic layer was separated, pooled together, dried over anhydrous Na_2_SO_4_, filtered, and concentrated to give a crude orange solid (187 mg). Column chromatography (1% acetone, 39% dichloromethane, 60% hexane, R_f_ 0.25) was performed and provided the pure product **8v** as an orange solid. (45 mg, 27%). ^1^H NMR (CDCl_3_): δ 13.03 (s, 1H), 8.04 (d, 1H, *J* = 16 Hz), 7.85 (d, 1H, *J* = 16 Hz), 7.40 (m, 13H), 6.67 (m, 2H), 6.55 (d, 1H, *J* = 8.1 Hz), 5.18 (s, 2H), 3.96 (q, 2H) 1.40 (t, 3H). ^13^C NMR (CDCl_3_): δ 194.8, 164.7, 160.1, 156.9, 141.1, 140.5, 136.2, 135.9, 134.6, 133.8, 130.6, 129.2, 128.7, 127.9, 127.4, 127.1, 126.2, 113.7, 112.8, 112.4, 111.2, 102.8, 77.2, 71.1, 64.3, 14.7. HRMS for C_30_H_25_BrO_4_ (M+H): theory 531.0993, found 531.0979. mp 155–157 °C.

### 2.21. (2E)-1-[2-(Biphenyl-4-Ylmethoxy)-6-Hydroxy-Phenyl]3-(5-Bromo-2-Methoxy-Phenyl)-Propenone (***8w***)

5-Bromo-2-methoxybenzaldehyde (61 mg, 0.29 mmol), 1-[2(biphenyl-4-ylmethyoxy)6-hydroxy)-phenyl]ethanone (**7d**) (91 mg, 0.29 mmol), and MeOH (2.5 mL) were combined, and KOH in methanol (40% weight/volume, 2.5 mL) was added at rt and then heated to 60 °C. The reaction mixture was stirred at 60 °C overnight. The workup included evaporation of the solvent under reduced pressure and the dropwise addition of 4 N HCl until the solution was at pH 1. The solution was then extracted with ethyl acetate (thrice). Each organic layer was separated, pooled together, dried over anhydrous Na_2_SO_4_, filtered, and concentrated to give a crude orange solid (137 mg). Column chromatography (1% acetone, 49.5% dichloromethane, 50% hexane, R_f_ 0.31) was performed and provided the pure product **8w** as an orange solid. (33 mg, 23%). ^1^H NMR (CDCl_3_): δ 13.04 (s, 1H), 8.03 (d, 1H, *J* = 16 Hz) 7.86 (d, 1H, *J* = 16 Hz), 7.43 (m, 13H), 6.70 (d, 1H, *J* = 8.8 Hz), 6.65 (d, 1H, *J* = 8.6 Hz), 6.55 (d, 1H, *J* = 8.3), 5.19 (s, 2H), 3.75 (s, 3H). ^13^C NMR (CDCl_3_): δ 194.7, 164.7, 160.1, 157.5, 141.1, 140.5, 136.2, 135.9, 134.6, 133.8, 130.7, 129.2, 128.7, 127.9, 127.4, 127.4, 127.1, 126.1, 112.9, 112.7, 112.4, 111.2, 102.7, 77.2, 71.0, 55.7. HRMS for C_29_H_23_BrO_4_ (M+H): theory 517.0837, found 517.0827. mp 164–167 °C.

### 2.22. (2E)-1-(2-Benzloxy-6-Hydroxy-Phenyl)-5-(5-Hydroxy-2-Methoxy-Phenyl)-Propenone (***8x***)

5-Hydroxy-2-methoxybenzaldehyde (81 mg, 0.53 mmol), 1-(2-benzyloxy-6-hydroxy-phenyl)-ethanone [7] (**7b**, 130 mg, 0.54 mmol), and MeOH (2.5 mL) were combined, and KOH in methanol (40% weight/volume, 2.5 mL) was added at rt, and the reaction mixture was stirred at 60 °C overnight. The workup included evaporation of the solvent under reduced pressure and the dropwise addition of 4 N HCl until the solution was at pH 2. The solution was then extracted with ethyl acetate (thrice). Each organic layer was separated, pooled together, dried over anhydrous Na_2_SO_4_, filtered, and concentrated to give a crude orange solid (252 mg). Column chromatography (20% ethyl acetate:hexane, R_f_ 0.3) was performed and provided the pure product **8x** as an orange solid. (92 mg, 46%). ^1^H NMR (CDCl_3_): δ 13.59 (s, 1H), 8.15 (d,1H, *J* = 15.9 Hz), 7.82 (d, 1H, *J* = 15.9 Hz), 7.55 (m, 2H), 7.43 (m, 3H), 7.38 (t, 1H, *J* = 8.3 Hz), 6.77 (m 2H), 6.66 (dd, 2H, *J* = 8.3, 0.7 Hz), 6.54 (d, 1H, *J* = 8.1 Hz), 6.14 (d, 1H, *J* = 2.9 Hz), 5.13 (s, 2H), 4.08 (s, 1H), 3.77 (s, 3H). ^13^C NMR (CDCl_3_): δ 194.5, 165.5, 160.2, 153.1, 148.9, 137.7, 136.4, 135.9, 129.0,128.9, 128.8, 128.2, 127.5, 124.6, 118.5, 113.1, 112.7, 111.9, 111.5, 102.3, 71.2, 56.1. HRMS for C_23_H_20_O_3_: (M+H): theory 377.1384, found 377.1391. mp 164–167 °C.

### 2.23. (2E)-1-(2-Benzloxy-6-Hydroxy-Phenyl)-3-(2,3,4-Trimethoxy-Phenyl)-Propenone (***8y***)

2,3,4-Trimethoxybenzyaldehyde (98 mg, 0.5 mmol), 1-(2-benzyloxy-6-hydroxy-phenyl)-ethanone [7] (**7b**, 121 mg, 0.5 mmol), and MeOH (2.5 mL) were combined, and KOH in methanol (40% weight/volume, 2.5 mL) was added at rt, and the reaction mixture was stirred at 60 °C overnight. The workup included evaporation of the solvent under reduced pressure and the dropwise addition of 4 N HCl until the solution was at pH 2. The solution was then extracted with ethyl acetate (thrice). Each organic layer was separated, pooled together, dried over anhydrous Na_2_SO_4_, filtered, and concentrated to give a crude orange solid (225 mg). Column chromatography (20% ethyl acetate:hexane, R_f_ 0.27) was performed and provided the pure product **8y** as an orange solid. (148 mg, 70%). ^1^H NMR (CDCl_3_): δ 13.49 (s, 1H), 8.06 (d, 1H, *J* = 15.9 Hz), 7.81 (d, 1H, *J* = 15.9 Hz), 7.48 (m, 2H), 7.36 (m, 4H), 6.66 (m, 2H), 6.52 (d, 1H, *J* = 8.3 Hz), 6.43 (d, 1H, *J* = 8.8 Hz), 5.14 (s, 2H), 3.89 (d, 6H, *J* = 5.1 Hz), 3.85 (s, 3H). ^13^C NMR (CDCl_3_): δ 194.5, 165.3, 160.1, 155.6, 153.7, 142.2, 138.1, 136.0, 135.6, 128.8, 128.3, 128.2, 126.4, 122.8, 122.3, 112.1, 111.4, 107.52, 102.5, 71.3, 61.7, 60.9, 56.1. HRMS for C_25_H_24_O_6_ (M+H): theory 421.1646, found 421.1659. 130–138 °C.

### 2.24. (2E)-1-(2-Benzloxy-6-Hydroxy-Phenyl)-3-(3,4,5-Trimethoxy-Phenyl)-Propenone (***8z***)

3,4,5-Trimethoxybenzyaldehyde (98 mg, 0.5 mmol), 1-(2-benzyloxy-6-hydroxy-phenyl)ethanone [7] (**7b**, 121 mg, 0.5 mmol), and MeOH (2.5 mL) were combined, and KOH in methanol (40% weight/volume, 2.5 mL) was added at rt, and then, the reaction mixture was stirred at 60 °C overnight. The workup included evaporation of the solvent under reduced pressure and the dropwise addition of 4 N HCl until the solution was at pH 2 The solution was then extracted with ethyl acetate (thrice). Each organic layer was separated, pooled together, dried over anhydrous Na_2_SO_4_, filtered, and concentrated to give a crude orange oil (222 mg). Column chromatography (20% ethyl acetate:hexane, R_f_ 0.27) was performed and provided the pure product **8z** as an orange solid. (116 mg, 55%). ^1^H NMR (CDCl_3_): δ 13.09 (s, 1H), 7.79 (d, 1H, *J* = 15.4 Hz), 7.72 (d, 1H, *J* = 15.7 Hz), 7. 40 (m, 3H), 7.25 (m, 3H), 6.66 (dd, 2H, *J* = 8.4 Hz, *J* = 0.9 Hz), 6.62 (s, 2H,), 6.54 (d, 1H, *J* = 7.3 Hz), 5.17 (s, 2H), 3.88 (s, 3H), 3.67 (s, 6H). ^13^C NMR (CDCl_3_): δ 194.3, 164.8, 160.0, 153.2, 143.1, 140.2, 135.9, 135.9, 130.5, 128.7, 128.2, 127.1, 112.4, 111.4, 105.8, 102.8, 71.1, 60.9, 56.0. HRMS for C_25_H_24_O_6_ (M+H): theory 421.1646, found 421.1652. mp 103-105 °C.

### 2.25. (2E)-1-(3,5-Dibromo-2-Hydroxyphenyl)-3-(3,4-Dimethoxyphenyl)-2-Propen-1-One (***NM5***, ***3***)

(2E)-1-(3,5-Dibromo-2-hydroxyphenyl)-3-(3,4-dimethoxyphenyl)-2-propen-1-one (**3**: **NM5**, see Figure 1) was prepared as described by Mateeva et al. [6] with a few changes. 3,4-Dimethoxybenzyaldehyde (166 mg, 1 mmol), 1-(3,5-dibromo-2-hydroxyphenyl)ethanone (294 mg, 1 mmol), and MeOH (5 mL) were combined, and KOH in methanol (40% weight/volume, 5 mL) was added at rt, and then, the mixture was heated to 60 °C and stirred at 60 °C for 3h. The workup included cooling the mixture to rt, evaporation of the solvent under reduced pressure, and the dropwise addition of 4 N HCl until the solution was at pH 2. The solution was then extracted with ethyl acetate (thrice). Each organic layer was separated, pooled together, dried over anhydrous Na_2_SO_4_, filtered, and concentrated to give a crude orange solid (478 mg). Column chromatography (15% ethyl acetate:hexane, R_f_ 0.23) was performed and provided the pure product **3** (**NM5**) as an orange solid. (356 mg, 81%). ^1^H-NMR matched the literature spectrum [23]. ^1^H NMR (CDCl_3_): δ 13.69 (s, 1H), 8.00–7.94 (m, 2H), 7.87 (d, 1H, *J* = 2.2 Hz), 7.39 (d, 1H, *J* = 15.2 Hz), 7.30 (dd, 1H, *J* = 8.3, 2 Hz), 7.18 (d, 1H, *J* = 2 Hz), 6.93 (d, 1H, *J* = 8.3 Hz), 3.99 (s, 3H), 3.96 (s, 3H). ^13^C NMR (CDCl_3_): δ 192.2, 159.3, 152.5, 149.5, 148.0, 141.1, 131.0, 127.1, 124.4, 121.7, 116.4, 113.4, 111.2, 110.5, 110.2, 56.2, 56.1. HRMS for C_17_H_14_Br_2_O_4_ (M-H)^−^: theory 438.9186 found 438.9174.

## 3. Results

### 3.1. Synthesis

**Figure 2:**
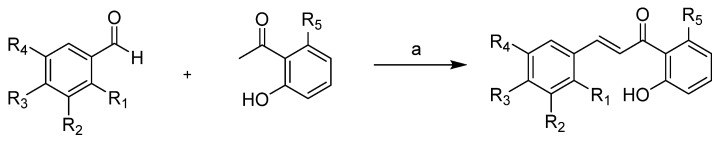
Reagents: (a) 40% KOH/MeOH, 60 °C.

As shown in Figure 2, the chalcones were synthesized using crossed aldol condensation between a substituted benzaldehyde and a substituted acetophenone derivative in KOH in methanol (40% *wt*/*v*) at 60 °C.

**Figure 3:**
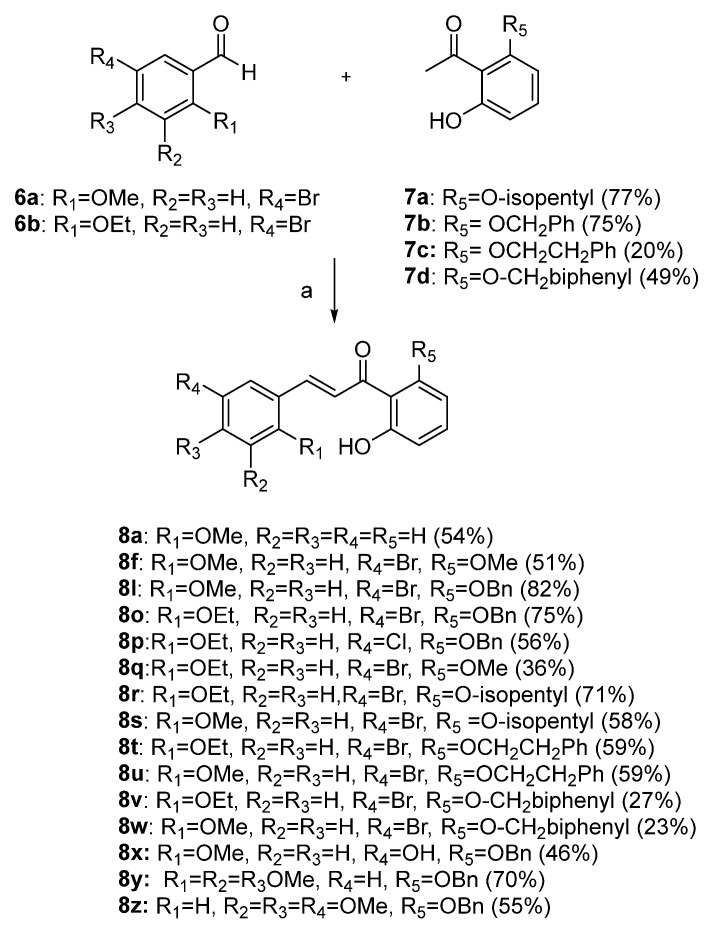
Synthesis of a chalcone library **8a**–**8z** using crossed aldol condensation. Note: compounds **7a** and **7c** were previously reported [21,22]. Compounds **8a**, **8f**, **8l**, **8o**, and **8p** were reported previously as were the missing entries: **8b**–**e**, **8g**–**k**, **8m**, and **8n** [7,10].

Specific chalcones were synthesized to expand upon the earlier chalcone library as shown in Figure 3. Earlier work had identified the importance of the R_1_, R_4_, and R_5_ substituents, and we sought to expand the library to better understand the structure–function relationships. While many of the benzaldehydes were commercially available, several acetophenone derivatives had to be synthesized via *O*-alkylation of the dihydroxy precursor (**7a**–**7d**). Chalcones **8a**, **8f**, **8l**, **8o**, **8p**, and **8q**–**8z** were synthesized via Figure 3 in good yield [7,10]. In the synthesis of **8v** and **8w**, the bulky O-CH_2_-biphenyl groups imparted reduced solubility and gave lower yields (27% and 23%, respectively) during the condensation step. All final compounds were characterized via ^1^H, ^13^C NMR, mass spectrometry and elemental analysis or HPLC (purity check; ≥95% pure) prior to conducting the biological experiments (see Experimental).

With the chalcones in hand, we then evaluated their growth-inhibition properties in Chinese hamster ovary (CHO-K1) cells and human Hs27 fibroblast cells. These studies provided insight into the maximum tolerated concentration of each chalcone before affecting growth by determining the EC_10_ value, which is the highest concentration of chalcone that results in 90% relative new cell growth after 48 h of incubation at 37 °C (5% CO_2_) compared to the untreated control in mammalian (CHO-K1) or human (Hs27) cells. The EC_50_ value was also determined in each cell line and is the effective concentration of chalcone needed to inhibit 50% of the relative new cell growth after 48 h of incubation at 37 °C (5% CO_2_). The results are shown in Table 2 along with a SAR summary of our findings in the host Hs27 cells.

As shown in Table 2, the EC_10_ values of the series ranged from 1 to 9.3 µM in CHO-K1 cells and 0.6 to 9.4 µM in Hs27 cells. The EC_50_ values ranged from 2.6 to >50 µM in CHO-K1 cells and 1.8 to 20.3 µM in Hs27 cells. The parent scaffold **8a** was typically the least growth-inhibitory as indicated by the higher EC_50_ value in both cell lines. Compounds **8p** (CHO-K1: 25.8 µM; Hs27: 19.3 µM) and **8v** (CHO-K1: >50 µM; Hs27: 18.4 µM) were also relatively the least growth-inhibitory as indicated by their higher EC_50_ values in both cell lines. Most other members of the series had 48 h EC_50_ values ≤10 µM in CHO-K1 cells. In Hs27 cells, most other members of the series had 48 h EC_50_ values ≤17 µM. We noted that compounds **8f** and NM5 were much more growth-inhibitory with EC_50_ values of 2.3 µM and 1.8 µM in Hs27 cells, respectively. Table 2 data provided concentrations that were well tolerated by the non-virally infected host cells (Hs27) and concentrations that began to affect cell growth of the non-infected host cells. It is important to note that the 48 h EC_50_ values here simply reflect the concentration of chalcone needed to inhibit 50% of the new cell growth and that the originally plated cells are still viable. In sum, these studies provided working concentration ranges for our subsequent viral studies.

### 3.2. Comparison of Cytotoxicity and Anti-Viral Effect of Chalcones ***8a***, ***8f***, ***8l***, ***8o***, ***8p***, and ***8q***–***8z*** in a Fibroblast Cell Culture

**Figure 4:**
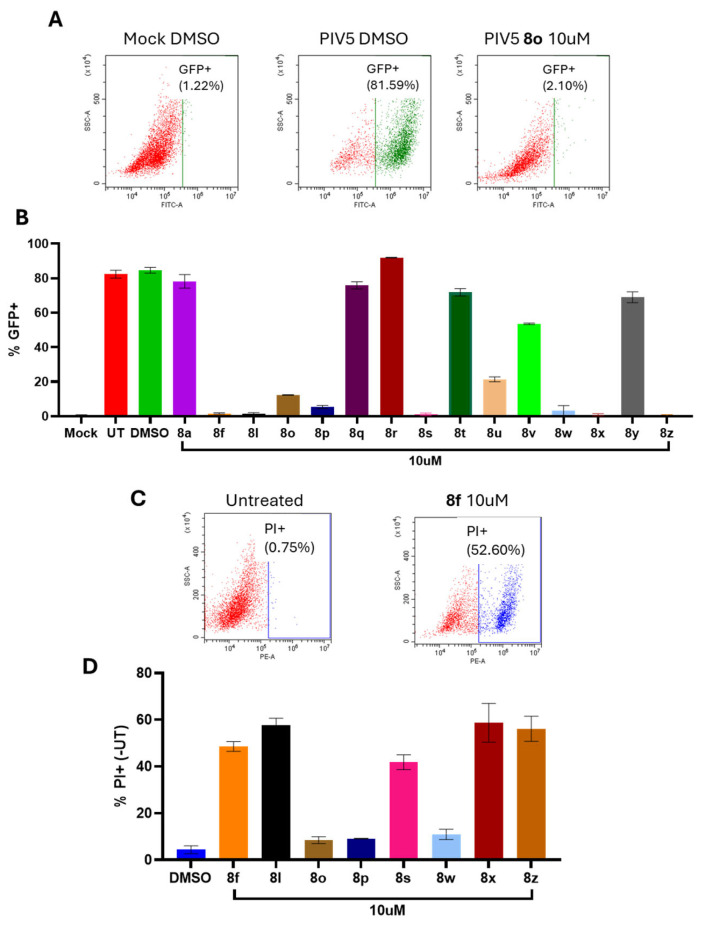
Comparison of cytotoxicity and anti-viral effect of chalcones **8a**, **8f**, **8l**, **8o**, **8p**, and **8q**–**8z** in a fibroblast cell culture. (**A**,**B**) Hs27 cells were treated with DMSO or with each chalcone at a concentration of 10 µM. After 24 h, cells were mock-infected or infected with PIV5-GFP at an MOI of 10 and cultured with chalcone-supplemented media at a concentration of 10 µM. At 24 hpi, cells were analyzed via flow cytometry for GFP expression. The loss of GFP expression with chalcone treatment is consistent with the inhibition of PIV5 replication. (**A**) Representative GFP flow cytometry scatter plots are shown. (**C**,**D**) Hs27 cells were treated with DMSO or with the indicated chalcone at a concentration of 10 μM for 24 h. After staining with propidium iodide (PI), cells were analyzed via flow cytometry. (**C**) Representative flow cytometry scatter plots for the control and **8f** are shown. (**D**) The percentage of the cell population that was PI+ is shown for each chalcone.

To measure the effect of chalcone treatment on PIV5 virus growth, Hs27 cells were infected with PIV5-GFP. This engineered negative sense single-stranded RNA virus expresses GFP, which serves as a genome-encoded protein readout. After entering a cell, PIV5 must first undergo primary transcription to convert its genome into mRNA. As genome replication begins, nascent genomes then become templates for further conversion to mRNA and viral gene expression becomes amplified in a process called secondary transcription. For viral proteins to be detectable in cell culture, this amplification must first take place, making the expression of GFP a direct indicator of whether secondary transcription has successfully occurred [24]. Hs27 cells were treated with 10 μM of each chalcone for 24 h and then mock infected or infected at a high multiplicity of infection (MOI) with PIV5-GFP. Cells were then cultured in media supplemented with the same chalcone before being processed for flow cytometry at 24 h post-infection (hpi). As shown in the representative data in Figure 4A, ~82% of DMSO-treated PIV5-GFP infected cells were positive for GFP expression. This was reduced to ~2% GFP-positive cells after treatment with **8o**. The ability of each chalcone to reduce viral GFP expression is shown in Figure 4B, with chalcones **8f**, **8l**, **8o**, **8p**, **8s**, **8w**, **8x**, and **8z** exhibiting the most notable performance against PIV5 infection. These chalcones were therefore selected for further screening for cytotoxicity.

To determine the actual cytotoxic effects of select chalcones in the series, human fibroblast Hs27 cells were treated with DMSO as a control or treated with each selected chalcone at a concentration of 10 µM. At 24 h post-treatment (hpt), the Hs27 cells were stained with propidium iodide (PI), a stain for cells with permeable plasma membranes, and then processed for flow cytometry (see Figure 4). An untreated control was subtracted from all samples to account for background staining, and the PI staining of untreated Hs27 cells showed minimal positive PI staining (Figure 4C). Similarly, DMSO-treated samples showed minimal increases in PI staining when compared to untreated controls (Figure 4D). As shown in Figure 4D, the treatment of Hs27 cells with compounds **8o** and **8p** each resulted in <10% of cells being PI+, with **8w** treatment resulting in ~11% of cells being PI+. By contrast, treatment with **8f**, **8l**, **8s**, **8x**, and **8z** resulted in ~40–60% of cells being positive for PI staining. These cytotoxicity findings reflected the relative growth-inhibition properties of **8f**, **8l**, **8o**, **8p**, **8s**, **8w**, **8x**, and **8z** observed in Table 2 (see Hs27 cell data). The significant difference in toxicity (as measured based on the % PI+ cells) between **8l** (R_1_ = OMe, 57.7% PI+) and **8o** (R_1_ = OEt; 8.5% PI+) at 10 µM revealed the importance of the R_1_ substituent in contributing to the toxicity of the compound, when the other chalcone substituents are identical (e.g., Br, OBn).

### 3.3. Chalcone Treatment of Hs27 Cells Inhibits PIV5-GFP Replication

**Figure 5:**
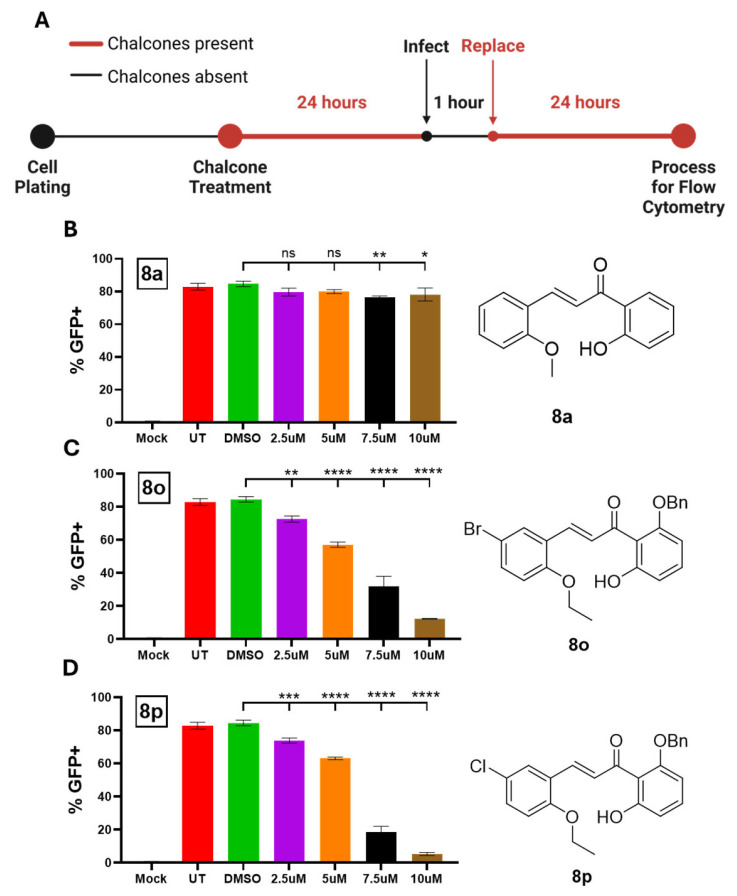
Chalcone treatment of Hs27 cells inhibits PIV5-GFP replication. (**A**) Schematic experimental design is shown for testing the effect of chalcones on infection and cell processing. Hs27 cells were treated with DMSO or treated with **8a** (**B**), **8o** (**C**), or **8p** (**D**) at a concentration of 2.5, 5, 7.5, or 10 µM. After 24 h, cells were mock infected or infected with PIV5-GFP at an MOI of 10 and then cultured with chalcone-supplemented media at a concentration of 2.5, 5, 7.5, or 10 µM. At 24 hpi, the Hs27 cells were analyzed via flow cytometry for GFP expression. ns = not significant, * indicates a *p*-value < 0.05, ** indicates a *p*-value < 0.01, *** indicates a *p*-value < 0.001, and **** indicates a *p*-value < 0.0001.

To determine the ability of **8a** (control), **8o**, and **8p** to inhibit PIV5-GFP protein expression at various concentrations, Hs27 cells were left untreated, DMSO-treated as a control, or treated with each chalcone at 2.5, 5, 7.5, or 10 µM. At 24 hpt, cells were mock infected or infected with PIV-GFP at a high MOI; then, media were immediately replaced with chalcone-supplemented media. At 24 hpi, GFP-positive cells were quantified using flow cytometry (see experimental design shown in Figure 5A). As shown in Figure 5B, ~80% of PIV5-GFP-infected Hs27 cells were GFP-positive when left untreated or treated with DMSO as a control. There was a minimal effect of increasing the concentration of **8a** on GFP expression, with GFP-positive cells remaining near 80%. In contrast, **8o** (Figure 5C) and **8p** (Figure 5D) showed a dose-dependent decrease in GFP expression with potent GFP expression inhibition seen at 10 μM (<10% GFP+). In combination with the minimal cytotoxic effects of **8o** and **8p** seen in Figure 4D, these results supported the further characterization of **8o** and **8p** as antivirals.

### 3.4. Chalcone Antiviral Effects Occur at an Early Stage of the PIV5-GFP Replication Cycle

**Figure 6:**
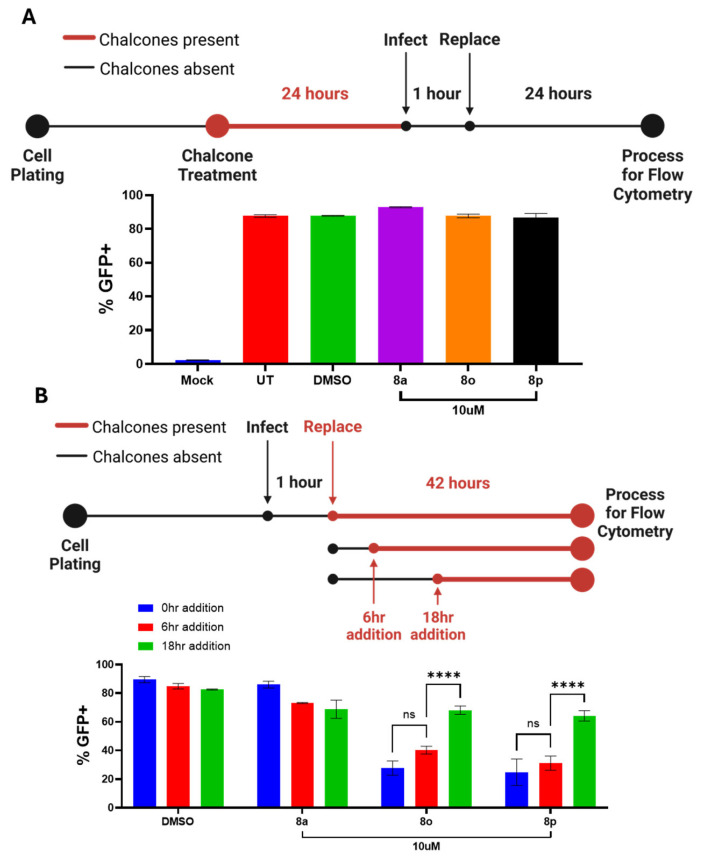
Chalcone antiviral effects occur at an early stage of the PIV5-GFP replication cycle. (**A**) Experimental design for testing chalcone presence in media at the pre-infection stage only. Hs27 cells were treated with DMSO or with the indicated chalcone at a concentration of 10 µM. After 24 h, cells were mock infected or infected with PIV5-GFP at an MOI of 10 then cultured in media lacking chalcones. At 24 hpi, cells were analyzed via flow cytometry. (**B**) Experimental design for testing time-of-addition of chalcones following infection. Hs27 cells were mock infected or infected with PIV5-GFP at an MOI of 10. Media containing the indicated chalcones were added at time 0, 6, or 18 hpi. Cells were analyzed for GFP expression via flow cytometry at 42 hpi. ns = not significant, and **** indicates a *p*-value < 0.0001.

To determine how the time of addition of chalcones affected their ability to inhibit viral protein expression, Hs27 cells were treated with 10 μM of each chalcone overnight, mock infected or infected with PIV5-GFP at a high MOI, and then incubated in media lacking chalcone. At 24 hpi, cells were analyzed for GFP expression using flow cytometry (experimental design shown in Figure 6A). As shown in Figure 6A, ~80% of Hs27 cells were GFP-positive when left untreated or treated with DMSO. The pre-infection treatment of cells with **8a**, **8o**, and **8p** resulted in no significant inhibition of GFP expression. These results demonstrated that when the chalcones were only present during the pre-infection period they were not effective at inhibiting the post-infection viral GFP expression in Hs27 cells.

A time-of-addition experiment was then carried out to determine the time post-infection at which the chalcones inhibited viral protein expression. Hs27 cells were mock infected or infected with PIV5-GFP at a high MOI. Chalcones were then added at 10 μM to cell cultures at 0, 6, or 18 hpi. At 42 hpi, GFP expression was quantified using flow cytometry (see the design shown in Figure 6B). As shown in Figure 6B, ~80% of Hs27 cells were GFP-positive regardless of the time of addition in DMSO-treated controls or with treatment with the control chalcone **8a**. The addition of chalcones **8o** and **8p** at 0 (or 6 hpi) significantly reduced the number of GFP-positive cells (to near 25%). By 18 hpi, the addition of chalcones **8o** and **8p** had no significant effect on the number of GFP-positive cells. These results indicate that the treatment of cells immediately following infection (i.e., 0–6 hpi) is sufficient to inhibit viral protein expression. However, this inhibition is diminished when chalcone addition occurs at later time points. Together, these data suggest the chalcones target an early stage of the viral replication cycle.

### 3.5. Chalcones Inhibit Spread of PIV5-GFP Through a Population of Fibroblast Cells

**Figure 7:**
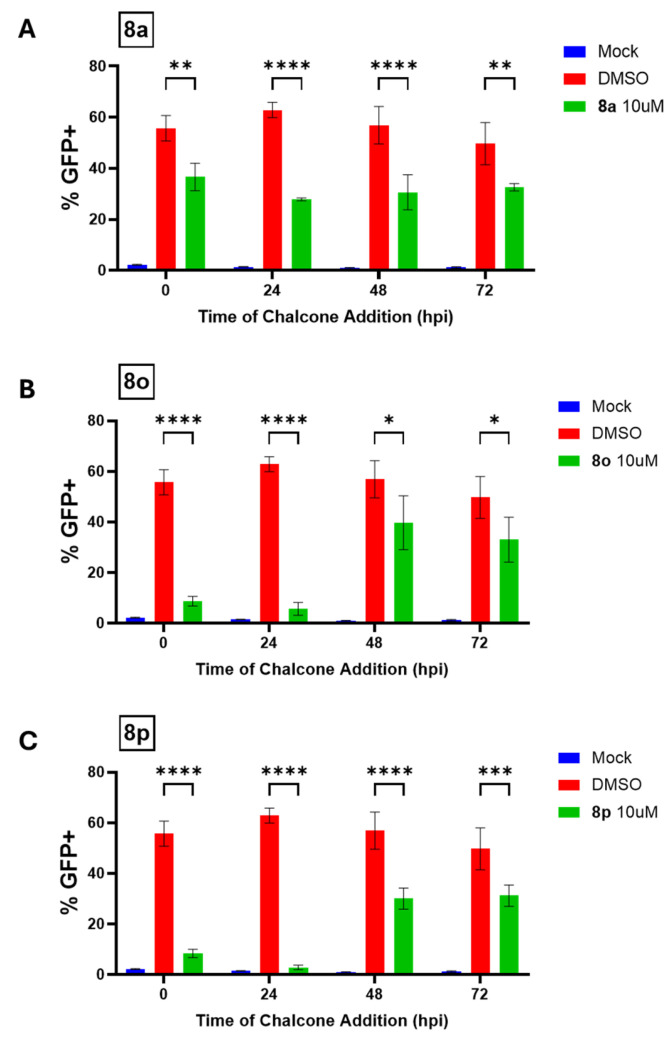
Chalcones inhibit spread of PIV5-GFP through a population of fibroblast cells. Hs27 cells were mock infected or infected with PIV5-GFP at an MOI of 0.1. At 0, 24, 48, or 72 hpi, cells were treated with DMSO or chalcone **8a** (**A**), **8o** (**B**), or **8p** (**C**) at a concentration of 10 µM. Cells were analyzed for GFP expression at 96 hpi. * indicates a *p*-value < 0.05, ** indicates a *p*-value < 0.01, *** indicates a *p*-value < 0.001, and **** indicates a *p*-value < 0.0001.

The above experiments were all performed at a high MOI in which the infection is synchronized across the cell population. To determine whether chalcones could reduce virus spread through a population of naïve cells, Hs27 cells were mock infected or infected with PIV5-GFP at a low MOI. Chalcones were then added to cell cultures at a concentration of 10 μM at 0, 24, 48, or 72 hpi, and GFP expression was quantified via flow cytometry at 96 hpi. As shown in Figure 7A, the number of GFP-positive Hs27 cells in DMSO-treated cultures reached nearly 60% by 96 hpi. Addition of the control **8a** modestly reduced the number of GFP-positive cells at all times post-infection (Figure 7A). Most importantly, the addition of **8o** or **8p** at 0 and 24 hpi significantly reduced GFP-positive cells to less than 10%, but this inhibition was not seen when compounds were added at the later time points 48 and 72 hpi (Figure 7B,C). These data indicate that the addition of chalcone **8o** or **8p** as late as 24 hpi can dramatically reduce the spread of the virus to the remaining naïve cells.

### 3.6. Chalcones Inhibit Expression of PIV5-GFP Genes and Proteins in Infected Fibroblasts

To better understand how the chalcones were inhibiting virus growth, RT-qPCR was utilized to determine the effect of chalcone treatment on viral gene expression. Hs27 cells were treated with DMSO as a control or treated with **8o** at a concentration of 10 µM. At 24 hpt, cells were mock infected or infected with PIV5-GFP at a high MOI and immediately replaced with media supplemented with **8o**. Total RNA was collected at 2, 5, and 10 hpi and used in RT-qPCR assays to measure levels of the PIV5 genes: fusion (F) protein, hemagglutinin-neuraminidase (HN) protein, and nucleocapsid (NP) protein. Collection at various time points allows one to kinetically distinguish between the chalcone-mediated inhibition of early and late viral gene expression. Values were normalized to actin and were set relative to the DMSO-treated control of each corresponding time point.

**Figure 8:**
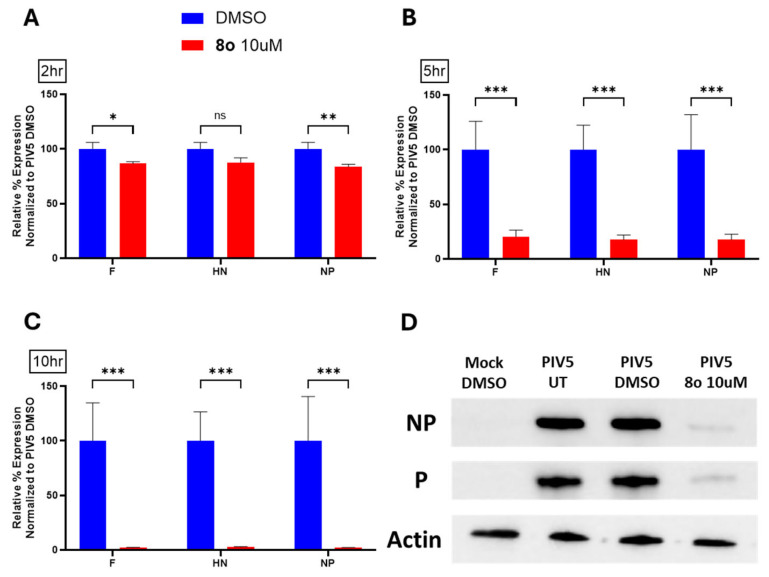
Chalcone **8o** inhibits expression of PIV5-GFP genes and proteins in infected fibroblasts. Hs27 cells were treated for 24 h with DMSO or with the chalcone **8o** at a concentration of 10 µM. Cells were then mock-infected or infected with PIV5-GFP at an MOI of 10 and cultured in chalcone-supplemented media at a concentration of 10 µM. (**A**–**C**) Total RNA was then harvested at 2 hpi (**A**), 5 hpi (**B**), or 10 hpi (**C**) and analyzed via RT-qPCR. Values are shown as relative percent expression normalized to the DMSO-treated infected controls for each corresponding time point. (**D**) Hs27 cells were cultured for 24 h in chalcone-supplemented media at a concentration of 10 μM and then mock infected or infected with PIV5-GFP. Cell lysates were collected 24 hpi and analyzed via western blotting for levels of PIV5 NP or P viral proteins. Lysates were normalized using actin. * indicates a *p*-value < 0.05, ** indicates a *p*-value < 0.01, *** indicates a *p*-value < 0.001, and ns = not significant. See Supplementary Materials for original blots.

As shown in Figure 8A, RNA collected 2 hpi shows minimal differences in viral gene expression when comparing the DMSO-treated control to infected cells treated with **8o**. Beginning at 5 hpi, however, there is a clear dramatic (~5 fold) reduction in viral gene expression in **8o**-treated infected cells. This effect is intensified by 10 hpi with a greater than 100-fold reduction in viral gene expression in **8o**-treated samples. These data indicate that while the number of viral gene transcripts begins similarly immediately following infection, treatment with **8o** can reduce the amplification of viral gene expression in infected cells as time progresses.

The above experiments utilized GFP expression and flow cytometry as an assay for observing the effects of chalcones on viral protein expression. To extend this analysis to bona fide viral proteins, cells were left untreated, DMSO-treated, or treated with the chalcone **8o** at 10 μM for 24 h. Cells were then infected with PIV5-GFP at a high MOI and then cultured in control media or media supplemented with **8o.** At 24 hpi, cell lysates were analyzed via western blotting for levels of cellular actin as a load control or for PIV5 proteins NP or P. As shown in Figure 8D, untreated and DMSO-treated PIV5-GFP infected cells show intense bands for both viral proteins. In contrast, lysates from **8o**-treated cells showed very little viral protein. Together, these data suggested that chalcone **8o** inhibits PIV5-GFP growth at an early stage (e.g., by 5 hpi) and results in reduced viral mRNA and protein expression.

### 3.7. ***8o*** Shows Optimal Balance Between the Maximum Tolerated Concentration and Half-Maximal Inhibitory Concentration

To compare these selected chalcones quantitatively, the respective IC_50_ values for inhibiting viral GFP expression and the 24 h maximum tolerated concentration, as determined through propidium iodide (PI) staining (24 h PI MTC) values, were determined. The GFP IC_50_ was defined as the concentration of chalcone required to inhibit the GFP expression (as measured via flow cytometry) by 50% when compared to DMSO-treated cells. The 24 h PI MTC was defined as the concentration of chalcone that resulted in 10% cell death as determined via PI staining and flow cytometry. To determine these PI MTCs, Hs27 cells were treated with each chalcone at various concentrations for 24 h and then analyzed via flow cytometry for positive PI staining. Untreated controls were subtracted to account for background staining. To determine GFP IC_50_ values, cells were treated with each chalcone at various concentrations for 24 h and then infected with PIV5-GFP. Immediately following infection, media were replaced with chalcone-supplemented media at specified concentrations. GFP expression was analyzed via flow cytometry at 24 hpi.

**Figure 9:**
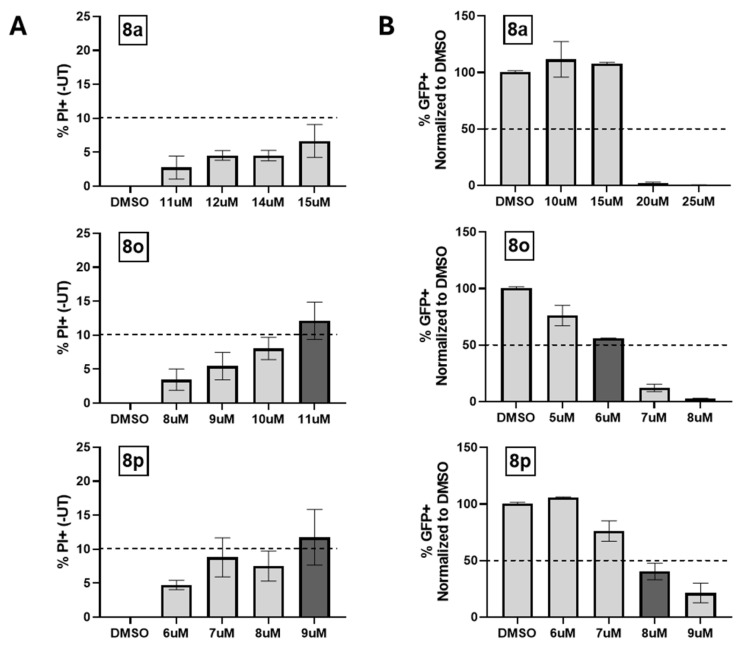
**8o** shows optimal balance between the maximum tolerated concentration and half-maximal inhibitory concentration. (**A**) Hs27 cells were DMSO-treated or treated with the indicated chalcone at varying concentrations. At 24 hpt, cells were stained with PI and then analyzed via flow cytometry. PI MTC is defined as 10% positive PI staining. Untreated controls were subtracted to remove background staining (<5% PI positive). (**B**) Hs27 cells were DMSO-treated or treated with each chalcone at varying concentrations to estimate the 24 h GFP IC_50_ value. After 24 h, cells were mock infected or infected with PIV5-GFP at an MOI of 10 and then cultured in chalcone-supplemented media at varying concentrations. At 24 hpi, cells were analyzed via flow cytometry for GFP expression. Values are expressed relative to DMSO-treated infected cells, set equal to 100%.

A potent antiviral would give a low GFP IC_50_ value indicating that the compound was able to inhibit viral GFP expression at a low concentration. Ideally, the antiviral should not affect host cell viability and should give a high PI MTC value. The quotient of these values (PI MTC/GFP IC_50_) was then calculated for the selected chalcones (Table 3). The higher this ratio (>1), the larger the implied therapeutic window where the antiviral benefits would not come with significant host cell toxicity. Chalcone **8l** displayed the lowest GFP IC_50_ at 5.0 μM (see Table 3), and **8o** displayed the highest 24 h PI MTC in fibroblasts with a concentration of 10.5 μM, as determined based on the generation of a standard curve using data in Figure 9. When one accounts for both the PI MTC and GFP IC_50_ (as determined based on the quotient value PI MTC/GFP IC_50_), **8o** displayed the best balance between cytotoxicity (as measured via % PI staining) and efficacy against viral GFP expression (as determined via flow cytometry for GFP+ cells).

These data provide a quantitative comparison between the compounds and indicate that **8o** is the compound with the greatest inhibitory effects on viral replication while maintaining the least cytotoxicity.

### 3.8. Chalcones Inhibit LACV, ZIKV, and Coronavirus Protein Expression in Fibroblast Cell Cultures

**Figure 10:**
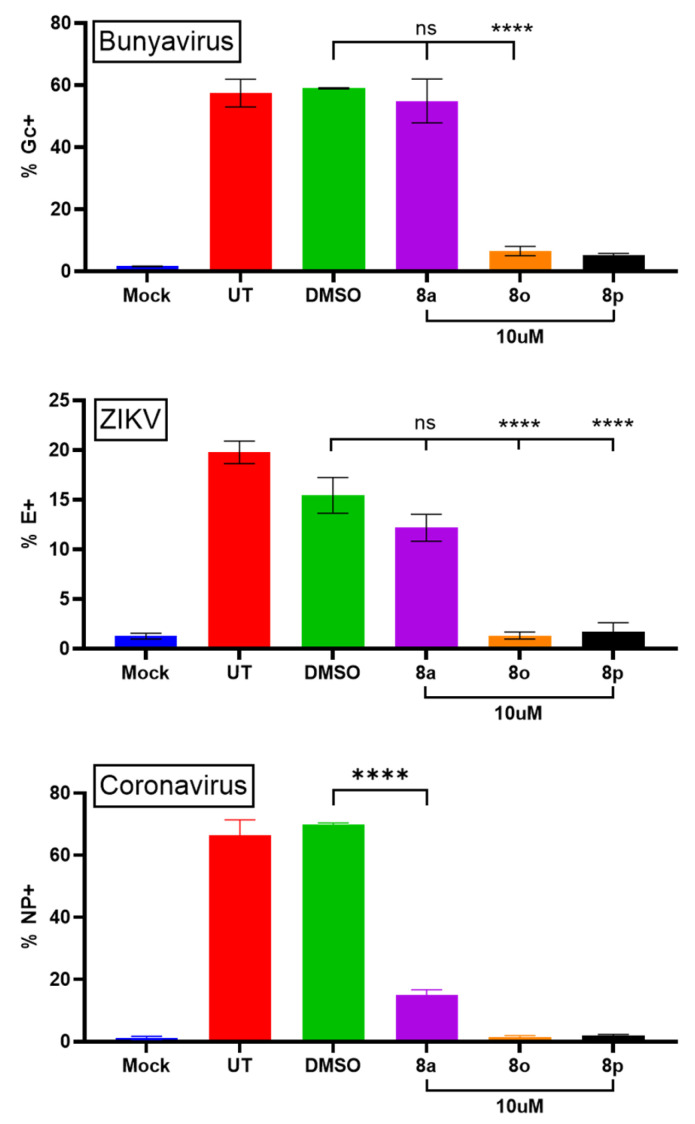
Chalcones inhibit LACV, ZIKV, and Coronavirus protein expression in fibroblast cell cultures. Hs27 cells were left untreated, treated for 24 h with DMSO, or treated with **8a**, **8o**, or **8p** at a concentration of 10 µM. Cells were then mock infected or infected with LACV (MOI 10), OC43 (MOI 50), or ZIKV (MOI 5) and cultured in chalcone-supplemented media at a concentration of 10 µM. Cells were analyzed at 24 hpi via flow cytometry using anti-Gc (LACV), anti-NP (OC43), or anti-E (ZIKV) antibodies. ns = not significant and **** indicates a *p*-value < 0.0001.

To expand these findings, we evaluated whether these top-performing chalcones inhibited the replication of other RNA viruses. Briefly described, Hs27 cells were left untreated, treated with DMSO as a control, or treated with the chalcones **8a**, **8o**, or **8p** at 10 μM for 24 h. Cells were then infected with the Bunyavirus LACV, the Flavivirus ZIKV, or the Coronavirus OC43 at high MOIs, as listed in Figure 10. Cells were then cultured for 24 h in media supplemented with the same chalcones before being processed for flow cytometry using antibodies specific for the viral proteins (Gc for LACV, E for ZIKV, NP for coronavirus OC43). As shown in Figure 10, the treatment of cells with **8o** and **8p** resulted in a dramatic reduction in the expression of the viral proteins for all three viruses when compared to the high-level expression in DMSO-treated samples. For LACV, expression of the glycoprotein Gc was reduced from ~60% of cells to below 10%. For ZIKV, envelope (E) protein expression was similarly dramatically reduced from 15% of cells in DMSO-treated controls to ~2% with **8o** and **8p** treatment. For OC43, NP protein expression was dramatically reduced from ~65% of cells in DMSO controls to ~1-2%. Interestingly, while compound **8a** did not inhibit viral gene expression for PIV5, LACV, or ZIKV, this chalcone showed significant inhibitory effects on OC43 NP expression. Together, these data indicate that specific chalcone designs can work as broad-spectrum anti-viral compounds that successfully inhibit prototype viruses from the Bunyavirus, Flavivirus, and Coronavirus families.

### 3.9. Chalcones Inhibit PIV5-GFP Protein Expression in Vero Cell Cultures

We also extended the analysis of chalcone anti-viral effects to the widely used Vero cell line. Select chalcones were screened in Vero cells to determine their 24 h PI MTC and 24 h GFP IC_50_ values following the methods outlined in Figure 9. The quotients of these values were determined to compare the compounds directly while accounting for both cytotoxicity and efficacy against PIV5. As shown in Table 4, the quotient values displayed similar results for each compound (PI MTC/GFP IC_50_ ratio ranged from 1.1 to 1.3 µM). Most notably, most of the values are markedly decreased when compared to Hs27 cells (Table 3), indicating a greater sensitivity of Vero cells to the chalcones. These data show the ability of chalcones to inhibit viral replication in different cell types and that the efficacy and cytotoxicity of the various chalcones can depend on the cell type.

### 3.10. ***8o*** Derivatives Show Greater Potency but Reduced PI MTC/GFP IC50 Quotients

**Figure 11:**
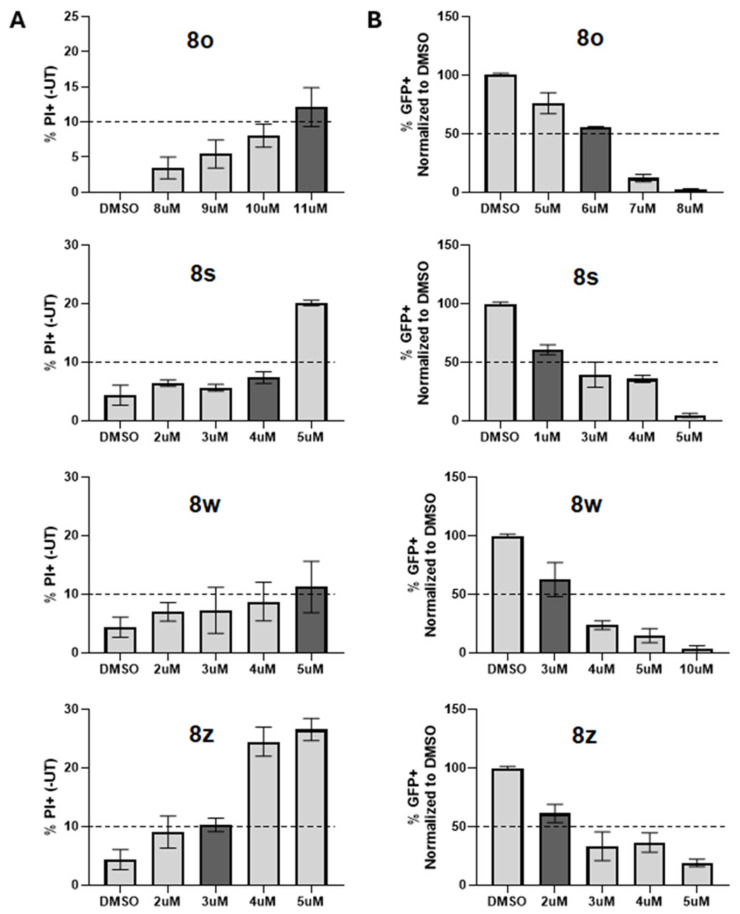
Derivatives of **8o** show greater potency but reduced PI MTC/GFP IC_50_ quotients compared to **8o**. Hs27 cells were treated with the indicated chalcones for 24 h as described in the legend of Figure 9. The percentages of PI+ cells are indicated in (**A**) data. Likewise, to determine the GFP IC_50_ values ((**B**) data), Hs27 cells were treated with the indicated chalcones for 24 h, and the percent GFP+ cells in the population were determined as described in the legend of Figure 9. Values are expressed relative to DMSO-treated infected cells set equal to 100%. The PI MTC is estimated as the concentration of chalcone needed to give a 10% PI+ readout. The 24 h GFP IC_50_ value is estimated as the concentration of chalcone needed to inhibit 50% of the GFP signal of the untreated DMSO containing control.

We compared how selected derivatives of **8o** also performed in the PIV5 assays. For example, compounds **8s**, **8w**, and **8z** were evaluated for their cytotoxicity. Briefly described, Hs27 cells were DMSO-treated or treated with each chalcone at various concentrations for 24 h (Figure 11A). PI MTC (10% PI+) values were determined via a standard curve and are summarized in Table 5. To determine 24 h GFP IC_50_ values, Hs27 cells were DMSO-treated or treated with each chalcone at various concentrations for 24 h and then infected with PIV5-GFP at a high MOI. Following infection, media were immediately replaced with chalcone-supplemented media and then processed for flow cytometry 24 hpi (Figure 11B).

The data is summarized in Table 5. While the new derivatives were more potent (lower GFP IC_50_ values), they were also more toxic and had significantly lower PI MTC values. We noted that **8w** and **8z** also had lower growth EC_50_ values in Table 2 consistent with their lower PI MTC values.

### 3.11. Changing the O-Benzyl Group in 8o Results in Decreased Ability to Inhibit PIV5-GFP

We investigated the role of the *O*-benzyl group in the design of **8o** (i.e., R_5_ substituent in Figure 1). As shown in Table 6, both the *O*-isopentyl (**8r**) and *O*-(biphenyl)methyl (**8v**) designs had higher EC_50_ values indicating that they were less growth-inhibitory to the host cells. The phenethyl derivative **8t** was more growth-inhibitory to the Hs27 host cells as evidenced by its lower EC_50_ value. In terms of their antiviral effects at 10 µM, their GFP IC_50_ values indicated that **8r**, **8t**, and **8v** had poor inhibition of viral growth compared to **8o**. Therefore, with this limited study, the *O*-benzyl group outperformed the other R_5_ substituents (Figure 1).

### 3.12. Chalcones ***8a***, ***8o***, ***8s***, ***8v***, and ***8w*** Do Not Inhibit Downstream mTOR Signaling

Since chalcones modulate cell growth as shown in Table 2, we investigated their effect on mTOR signaling. Indeed, the related structure NM5 (Figure 1) has been reported to inhibit mTOR signaling [6]. To explore this possibility, we measured the downstream ribosomal protein S6 (rps6) and phosphorylated ribosomal protein S6 (prps6) as readouts of mTOR signaling. Thus, the relative protein expression of rps6 and prps6 in the presence and absence of selected chalcones was determined.

**Figure 12:**
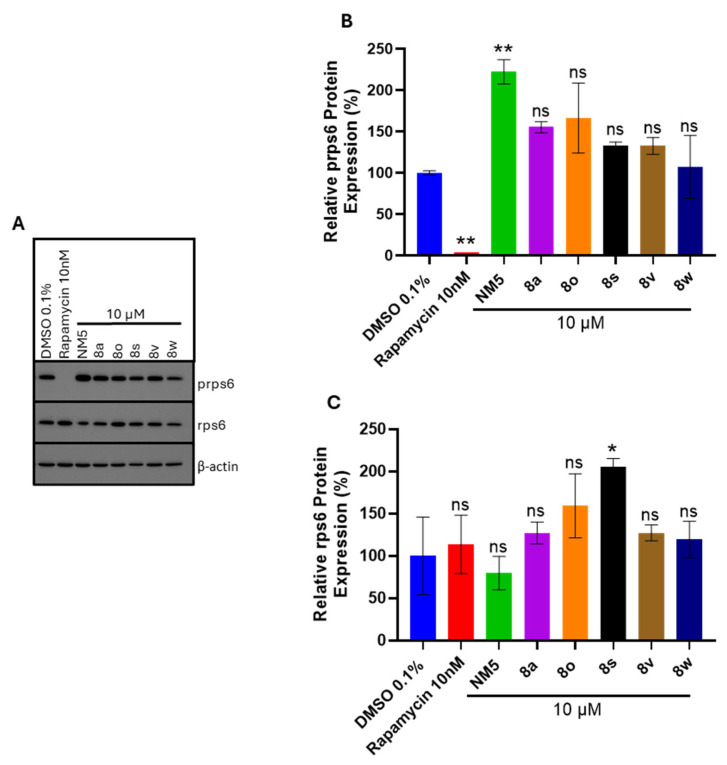
Chalcones at 10 µM do not modulate prps6 phosphorylation and do not inhibit mTOR signaling in PANC-1 cells. PANC-1 cells were treated with DMSO (0.1%), rapamycin (10 nM), or chalcones **NM5**, **8a**, **8o**, **8s**, **8v**, and **8w** (10 μM) for 72 h, and then, lysates were collected for protein analysis. The data shown was calculated from the average protein expression levels of two biological replicates. (**A**) Cell lysates were collected and analyzed via western blotting for the protein expression levels of prps6, total rps6, and β-actin. (**B**) Relative protein expression of prps6 in PANC-1 cells treated with rapamycin (10 nM) and chalcones **NM5**, **8a**, **8o**, **8s**, **8v**, and **8w** (10 μM) for 72 h. (**C**) Relative protein expression of rps6 in PANC-1 cells treated with rapamycin (10 nM) and chalcones **NM5**, **8a**, **8o**, **8s**, **8v**, and **8w** (10 μM) for 72 h. β-Actin was used as a protein loading control. * indicates a *p*-value < 0.05, ** indicates a *p*-value < 0.01, and ns indicates no significant differences. See Supplementary Materials for original blots.

PANC-1 cells were chosen for this study as they are highly dependent on mTOR for their growth [25]. PANC-1 cells were cultured and treated with the positive control rapamycin (10 nM) and chalcones NM5, **8a**, **8o**, **8s**, **8v**, and **8w** (10 μM) for 72 h. Rapamycin and the chalcones were dissolved in 100% DMSO and dosed so that the final DMSO concentration was 0.1%. Cell lysates were collected and analyzed to determine the relative protein expression levels of rps6, prps6, and β-actin as a loading control (see Figure 12).

Western blot analysis showed no significant changes for most of the chalcones except NM5 and **8s**, suggesting that mTOR signaling is not the target of these compounds. As expected, treatment with the known mTOR inhibitor rapamycin resulted in the loss of prps6 expression verifying rapamycin as a positive control of mTOR inhibition in PANC-1 cells. The effect of chalcone treatment on the relative protein expression of prps6 and rps6 is shown in Figure 12B,C. Chalcone **NM5** unexpectedly resulted in the largest difference in the expression of prps6 relative to the DMSO control and was found to stimulate this prps6 mTOR readout.

This surprising result contradicted the earlier report with **NM5** [6] and may be due to cell line differences. For example, the original observations with **NM5** were performed in the SGR2 and Huh7.5 cell lines, which are highly susceptible to HCV infection, whereas our study was performed in PANC-1 cancer cells, where compound **NM5** behaved like a mTOR stimulant [6]. The remaining chalcones **8a**, **8o**, **8s**, **8v**, and **8w** showed no significant change in prps6 expression compared to the DMSO control.

In terms of the other mTOR readout, rapamycin and the chalcones **8a**, **8o**, **8v**, and **8w** resulted in no significant difference in total rps6 protein expression. However, chalcone **8s** significantly increased rps6 expression suggesting a potential stimulating effect on overall total protein translation within the cell.

## 4. Discussion

The most striking finding from our virology studies was the broad-spectrum antiviral effects that our chalcones showed on prototype members of four different virus families, each of which is considered a prime candidate family for an emerging pandemic virus: Paramyxo-, Bunya-, Flavi-, and Corona-viruses. This broad inhibition of multiple divergent virus families is the major discovery of this report.

The observed pan anti-viral activities of **8o** and **8p** are intriguing. Each of these four viral families employ different strategies for replication, indicating that chalcones may target conserved viral replication mechanisms. How then do these compounds work?

We speculate that the chalcone-mediated inhibition of viral entry is not likely because all four viruses tested here bind to different cellular receptors to initiate the infection: sialic acid for PIV5, DC-SIGN for LACV, AXL for ZIKV, and TMPRSS2 for OC43 [26,27,28,29]. Similarly, some of these viruses enter cells via fusion at the plasma membrane (e.g., PIV5), while others are internalized through clathrin-mediated (e.g., ZIKV) or caveolin-1-dependent (e.g., OC43) endocytosis and fusion from internal vesicles [30,31,32]. However, as mentioned above, all four viruses share essential steps downstream of cell entry.

A common feature of these four viruses is that they are completely dependent on the production of a viral RNA-dependent-RNA polymerase (RDRP) from incoming genomes. Thus, a common mechanism of action of our chalcones may include inhibition of the viral RDRP. Our initial studies focused on the effect of chalcones on the replication of PIV5, a prototype member of the *Paramyxovirus* family of negative-strand RNA viruses. The replication cycle of these viruses depends on initial transcription by the virion-associated RDRP to produce low levels of mRNA, followed by genome replication, and finally secondary transcription to produce detectable levels of the GFP protein encoded in the PIV5-GFP reporter virus used here [24]. Time-of-addition studies showed that our chalcones inhibit viral RNA synthesis at an early stage of the virus growth cycle within 5-6 h post infection, which is within the timeframe when primary transcription is occurring. Since switching viral RDRP from carrying out primary transcription to genome replication requires the synthesis of new viral proteins, we speculate that the chalcones may target the production of viral genomes via the RDRP or act at the level of the translation of viral mRNAs.

Recent reports have suggested that chalcones can bind to the RDRP of SARS-CoV2, for example [33,34]. As such, RDRPs are excellent drug targets due to their typical low-level expression and specific enzymatic activities that are limited only to viral templates [35]. Future work will determine if our chalcone derivatives are interacting with the RDRPs of various RNA viruses to explain their mechanism of action.

Another possible mechanism of action of our chalcones could be independent of the viral machinery. We speculate that host cells with inhibited growth are poor viral hosts. In one scenario, chalcone disruption of host cell growth and proliferation pathways may create unfavorable intracellular environments for viral replication. Some reports suggest that chalcones can induce cell cycle arrest [36]. Our initial efforts to understand the mechanism of action of the lead chalcone **8o** in host cell signaling pathways essentially ruled out mTOR inhibition as a mechanism of action. Surprisingly, the published mTOR inhibitor (chalcone NM5) was the only chalcone to affect the activation of mTOR, as evidenced by a significant increase in rps6 phosphorylation (see Figure 12). Future work will analyze prps6 and rps6 protein expression in mock and infected Hs27 cells treated with the chalcone series to completely rule out mTOR signaling as the targeted pathway of infected cells.

We note that compound **8o** meets several criteria for Lipinski’s rule of 5 [37,38]. It has an MW <500 (453 g/mol) and has <5 H bond donors and <10 H bond acceptors. However, **8o** has a clog P of 5.84, which violates Lipinski’s Rule (clogP < 5). While there are documented violations to this rule of 5 [38], future designs should aim to install more water-soluble substituents to improve the drug-like properties and oral availability of these constructs for clinical applications. 

Our work represents an important contribution to this evolving research area by suggesting chalcones as a core scaffold for broad spectrum antivirals [39,40]. Other scaffolds have been suggested. For example, Patel et al. recognized structural similarity in the binding pocket of positive single stranded RNA viruses and the SARS-CoV2 3CL^pro^ viral protease and showed broad spectrum antiviral activity of SARS-CoV2 3CL^pro^ viral protease inhibitors [39]. Luong et al. recently highlighted the role of viral attachment inhibitors, fusion inhibitors, viral biosynthesis inhibitors and viral assembly and release inhibitors as potential broad-spectrum antivirals [40]. In addition, drug repurposing was recently used to develop pan-flavivirus compounds [41]. These collective efforts bring us closer to pan antivirals. Moving forward, however, important caveats must be addressed including host cell toxicity and viral resistance.

## 5. Conclusions

In closing, RNA viruses continue to pose a threat to global human health. In the absence of an effective vaccine, research into novel antiviral compounds is warranted due to drug resistance and a lack of broad-spectrum antivirals. Our preliminary mechanistic studies suggest that chalcones like **8o** inhibit an early step in the replication of these four important pathogenic viral families (paramyxovirus, bunyavirus, flavivirus, and coronavirus) each with a different replication strategy. While more effort is needed to fully understand the observations here and to improve the therapeutic range, this discovery is an important first step on the road to developing pan antivirals to address future global viral outbreaks. 

## 6. Patents

Phanstiel IV, O., Tschammer, N. Flavonoid based antiviral targets. US Patent 9629822, issued on 25 April 2017.

## Figures and Tables

**Table 1:** Actin and PIV5 gene primers utilized for RT-qPCR data.

Gene	Forward Primer	Reverse Primer
β-Actin	5′ GATCATTCGTCCTCCTGAGC 3′	5′ ACTCCTGCTTGCTGATCCAC 3′
F	5′ ACGTGTTATGGTGACTGGCA 3′	5′ GAACAGCACGAATCGAGTGA 3′
HN	5′ TGACCAACCCTTCGTCTACC 3′	5′ CTTGACCGCTTGATCCAAAT 3′
NP	5′ TGACCAGTCACCAGAAGCTG 3′	5′ CGGAATCAACGAAAGGTGTT 3′

**Table 2:** Forty-eight-hour growth inhibition data for chalcones in CHO-K1 and Hs27 cells ^a^.

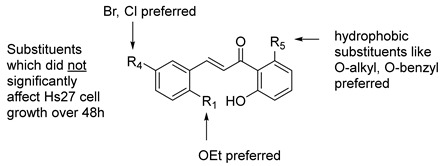				
Chalcone	CHOK1 48 h EC10 (µM)	CHOK1 48 h EC50 (µM)	Hs27 48 h EC10 (µM)	Hs27 48 h EC50 (µM)
**8a**	9.3 ± 0.6	25.5 ± 2.7	0.6 ± 0.04	20.3 ± 2.1
**8f**	2.5 ± 0.1	6.1 ± 0.8	1.4 ± 0.1	2.3 ± 0.2
**8l**	5.2 ± 0.3	8.1 ± 1.0	6.1 ± 0.5	7.8 ± 0.6
**8o**	7.0 ± 0.4	9.9 ± 0.7	8.3 ± 0.5	13.3 ± 1.1
**8p**	8.2 ± 0.3	25.8 ± 1.1	8.7 ± 0.7	19.3 ± 1.9
**8q**	4.3 ± 0.4	6.0 ± 0.3	2.7 ± 0.04	9.9 ± 1.0
**8r**	4.7 ± 0.4	6.8 ± 0.7	9.4 ± 0.7	16.9 ± 2.0
**8s**	5.4 ± 0.3	8.6 ± 0.3	6.9 ± 0.6	13.9 ± 0.5
**8t**	1.1 ± 0.1	2.6 ± 0.2	6.4 ± 0.5	10.3 ± 0.5
**8u**	1.0 ± 0.1	4.3 ± 0.3	3.4 ± 0.5	4.9 ± 0.6
**8v**	3.7 ± 0.3	>50	3.4 ± 0.2	18.4 ± 0.9
**8w**	6.8 ± 0.3	10.1 ± 1.0	6.5 ± 0.5	8.2 ± 0.5
**8x**	3.8 ± 0.4	6.3 ± 0.3	1.1 ± 0.07	3.4 ± 0.3
**8y**	7.2 ± 0.6	15.8 ± 1.0	6.3 ± 0.2	7.7 ± 0.2
**8z**	3.8 ± 0.4	5.8 ± 0.4	4.0 ± 0.2	5.0 ± 0.2
**NM5**	1.1 ± 0.1	6.2 ± 0.3	1.0 ± 0.1	1.8 ± 0.1

^a^ Each compound was dosed as a DMSO solution, and the final DMSO level in each well was 0.5%. Control experiments indicated that this level of DMSO did not affect cell growth. The SAR summary is based on Hs27 cell data, wherein certain substituents gave rise to higher EC_50_ values. The graphical summary above shows the preferred designs that had limited growth inhibition of the uninfected host cell.

**Table 3:** 24h PI MTC and 24 h GFP IC_50_ values in Hs27 cells ^a^.

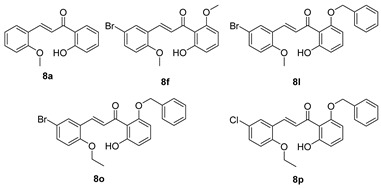			
Chalcone	PI MTC (µM)	GFP IC_50_ (µM)	PI MTC/GFP IC_50_
**8a**	>15	17.5	ND
**8f**	4.9	5.2	0.9
**8l**	7.0	5.0	1.4
**8o**	10.5	6.0	1.8
**8p**	8.4	7.9	1.1

^a^ The respective 24 h PI MTC and 24 h GFP IC_50_ values were calculated utilizing a standard curve from data in Figure 9, and the data is shown in Table 3. We estimate that the error in the PI MTC value is ~3% and ~5% for the GFP value.

**Table 4:** 24h PI MTC and 24 h GFP IC_50_ values in Vero cells ^a^.

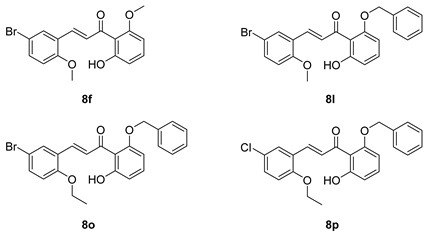			
Chalcone	PI MTC (µM)	GFP IC_50_ (µM)	PI MTC/GFP IC_50_
**8f**	6.8	5.7	1.2
**8l**	4.0	3.1	1.3
**8o**	2.5	2.2	1.1
**8p**	3.2	2.8	1.1

^a^ To determine PI MTC values, Vero cells were DMSO-treated or treated with each chalcone at varying concentrations. Cells were then stained with PI and processed for flow cytometry 24 hpt, and PI MTC values were determined using a standard curve. To determine GFP IC_50_ values, Vero cells were DMSO-treated or treated with each chalcone at varying concentrations. At 24 hpt, cells were mock infected or infected with PIV5-GFP at an MOI of 10. Cells were processed for flow cytometry and analyzed for GFP expression 24 hpi, and GFP IC_50_ values were determined using a standard curve. A summary of the PI MTC, GFP IC_50_, and PI MTC/GFP IC_50_ quotient values is shown in the table.

**Table 5:** Relative PI MTC and GFP IC_50_ values of **8o** derivatives in Hs27 cells.

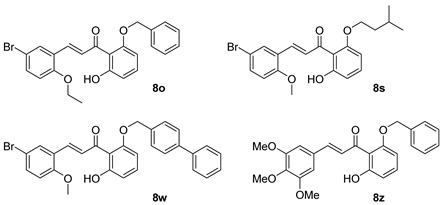				
Chalcone	Hs27 EC_50_ (µM)	PI MTC (µM)	GFP IC_50_ (µM)	PI MTC/GFP IC_50_
**8o**	13.3	10.5	6.0	1.8
**8s**	13.9	3.5	2.1	1.7
**8w**	8.2	4.5	3.3	1.4
**8z**	5.0	2.4	2.5	1.0

**Table 6:** Side chain comparisons in the design of **8o** via the determination of the EC_50_ and % GFP at 10 µM relative to DMSO-treated Hs27 cells.

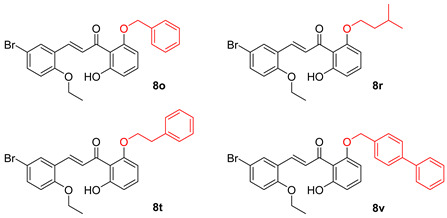		
Chalcone	Hs27 EC_50_ (µM)	% GFP relative to DMSO treated (10 µM)
**8o**	13.3	14.6
**8r**	16.9	108.7
**8t**	10.3	84.9
**8v**	18.4	63.3

## Data Availability

The data is available via the attached Supplementary Materials provided above.

## Supplementary Materials

The following supporting information can be downloaded at: https://www.mdpi.com/article/10.3390/biom15091285/s1, ^1^H NMR for compound **6b** and ^1^H NMR and ^13^C NMR data for compounds, **7a**–**7d**, **8q**–**8z**, and **NM5**, mass spectral data for **7d**, **8q**–**8z**, and **NM5**, HPLC purity checks for **8x**–**8z**, and Table S1. Elemental Analyses for **7d**, **8q**–**8w**, and **NM5.** Original Western blot images (see S32–S46 in Supplementary Materials).

## Abbreviations

The following abbreviations are used in this manuscript:

ATCC	American Type Culture Collection
AXL	tyrosine protein kinase receptor
BCA	bicinchoninic acid assay
BSA	bovine serum albumin
CDC	Center for Disease Control
CHO-K1	Chinese hamster ovary cells
DCM	dichloromethane
DC-SIGN	C-type lectin protein
DMEM	Dulbecco Modified Eagle Medium
DMF	N,N’-dimethylformamide
DMSO	dimethyl sulfoxide
DNA	deoxyribonucleic acid
EC50	concentration of compound needed to inhibit 50% of relative new growth compared to an untreated control
EtOAc	ethyl acetate
F	fusion protein
FBS	fetal bovine serum
GFP	green fluorescence protein
GFP IC_50_	the concentration of chalcone required to inhibit the GFP expression by 50% when compared to DMSO-treated cells.
HCMV	human cytomegalovirus
HCV	hepatitis C virus
HI FBS	heat-inactivated fetal bovine serum
HIV	human immunodeficiency virus
HN	hemagglutinin-neuraminidase protein
hpi	hours post infection
HPLC	high performance liquid chromatography
hpt	hours post treatment
HRP	horseradish peroxidase
LACV	LaCrosse virus
MDBK	Madin-Darby bovine kidney
MHz	megahertz
MOI	multiplicity of infection
mRNA	messenger RNA
mTOR	mammalian target of rapamycin
MTS	Promega Cell Titer 96 Aqueous non-radioactive cell proliferation reagent
NMR	nuclear magnetic resonance
NP	nucleocapsid protein
P	P viral protein
PBS	phosphate buffered saline
PI	propidium iodide
PI MTC	the concentration of chalcone that resulted in 10% cell death as determined by PI staining and flow cytometry.
PIV5	paramyxovirus parainfluenza virus 5
PIV5-GFP	paramyxovirus parainfluenza virus 5 expressing GFP
PVDF	polyvinylidene difluoride
RDRP	RNA-dependent-RNA polymerase
RIPA	radioimmunoprecipitation assay (buffer)
RNA	ribonucleic acid
RPMI	Roswell Park Memorial Institute
SDS	sodium dodecyl sulfate
SDS-PAGE	sodium dodecyl sulfate-polyacrylamide gel electrophoresis
TLC	thin layer chromatography
TMPRSS2	transmembrane protease serine 2
ZIKV	Zika virus

## References

[1] World Health Organization COVID-19 Cases, World. https://data.who.int/dashboards/covid19/cases?m49=001&n=c.

[2] Deschamps A.M., DeRocco A.J., Bok K., Patterson L.J. (2023). Prototype Pathogens for Vaccine and Monoclonal Antibody Countermeasure Development: NIAID Workshop Process and Outcomes for Viral Families of Pandemic Potential. J. Infect. Dis..

[3] Karim M., Lo C.W., Einav S. (2023). Preparing for the next viral threat with broad-spectrum antivirals. J. Clin. Investig..

[4] Irwin K.K., Renzette N., Kowalik T.F., Jensen J.D. (2016). Antiviral drug resistance as an adaptive process. Virus Evol..

[5] Rajendran G., Bhanu D., Aruchamy B., Ramani P., Pandurangan N., Bobba K.N., Oh E.J., Chung H.Y., Gangadaran P., Ahn B.C. (2022). Chalcone: A Promising Bioactive Scaffold in Medicinal Chemistry. Pharmaceuticals.

[6] Mateeva N., Eyunni S.V.K., Redda K.K., Ononuju U., Hansberry T.D., Aikens C., Nag A. (2017). Functional evaluation of synthetic flavonoids and chalcones for potential antiviral and anticancer properties. Bioorg. Med. Chem. Lett..

[7] Kralj A., Nguyen M.T., Tschammer N., Ocampo N., Gesiotto Q., Heinrich M.R., Phanstiel O. (2013). Development of flavonoid-based inverse agonists of the key signaling receptor US28 of human cytomegalovirus. J. Med. Chem..

[8] Gerhauser C. (2005). Beer constituents as potential cancer chemopreventive agents. Eur. J. Cancer.

[9] Padmavathi G., Roy N.K., Bordoloi D., Arfuso F., Mishra S., Sethi G., Bishayee A., Kunnumakkara A.B. (2017). Butein in health and disease: A comprehensive review. Phytomedicine.

[10] Cole A.L., Hossain S., Cole A.M., Phanstiel O. (2016). Synthesis and bioevaluation of substituted chalcones, coumaranones and other flavonoids as anti-HIV agents. Bioorg. Med. Chem..

[11] Lamb R.A., Parks G.D., Knipe D.M., Howley P.M., Griffin D.E., Lamb R.A., Martin M.A., Roizman B., Straus S.E. (2007). Paramyxoviridae: The viruses and their replication. Fields Virology.

[12] Elliott R.M. (1990). Molecular biology of the Bunyaviridae. J. Gen. Virol..

[13] Rucinski S.L., Binnicker M.J., Thomas A.S., Patel R. (2020). Seasonality of Coronavirus 229E, HKU1, NL63, and OC43 From 2014 to 2020. Mayo Clin. Proc..

[14] Miner J.J., Diamond M.S. (2017). Zika Virus Pathogenesis and Tissue Tropism. Cell Host Microbe.

[15] Matthews E., Chauhan L., Piquet A.L., Tyler K.L., Pastula D.M. (2022). An Overview of La Crosse Virus Disease. Neurohospitalist.

[16] Wansley E.K., Parks G.D. (2002). Naturally occurring substitutions in the P/V gene convert the noncytopathic paramyxovirus simian virus 5 into a virus that induces alpha/beta interferon synthesis and cell death. J. Virol..

[17] Cruz M.A., Parks G.D. (2020). La Crosse Virus Infection of Human Keratinocytes Leads to Interferon-Dependent Apoptosis of Bystander Non-Infected Cells In Vitro. Viruses.

[18] Kedarinath K., Fox C.R., Crowgey E., Mazar J., Phelan P., Westmoreland T.J., Alexander K.A., Parks G.D. (2022). CD24 Expression Dampens the Basal Antiviral State in Human Neuroblastoma Cells and Enhances Permissivity to Zika Virus Infection. Viruses.

[19] Fox C.R., Parks G.D. (2021). Complement Inhibitors Vitronectin and Clusterin Are Recruited from Human Serum to the Surface of Coronavirus OC43-Infected Lung Cells through Antibody-Dependent Mechanisms. Viruses.

[20] Parks G.D., Ward K.R., Rassa J.C. (2001). Increased readthrough transcription across the simian virus 5 M-F gene junction leads to growth defects and a global inhibition of viral mRNA synthesis. J. Virol..

[21] Yang H.-M., Shin H.-R., Cho S.-H., Song G.-Y., Lee I.-J., Kim M.-K., Lee S.-H., Ryu J.-C., Kim Y., Jung S.-H. (2006). The role of the hydrophobic group on ring A of chalcones in the inhibition of interleukin-5. Arch. Pharmacal Res..

[22] Valdameri G., Genoux-Bastide E., Peres B., Gauthier C., Guitton J., Terreux R., Winnischofer S.M., Rocha M.E., Boumendjel A., Di Pietro A. (2012). Substituted chromones as highly potent nontoxic inhibitors, specific for the breast cancer resistance protein. J. Med. Chem..

[23] Shih T., Chou C., Liao W., Hsiao C. (2014). Copper-mediated trimethylsilyl azide in amination of bromoflavonoids to synthesize unique aminoflavonoids. Tetrahedron.

[24] Robert A., Lamb G.D.P., Bernard N., Fields D.N.K., Howley P.M. (2013). Paramyxoviridae: The viruses and their replication. Fields Virology.

[25] Nguyen T.U., Hector H., Pederson E.N., Lin J., Ouyang Z., Wendel H.-G., Singh K. (2023). Rapamycin-Induced Feedback Activation of eIF4E-EIF4A Dependent mRNA Translation in Pancreatic Cancer. Cancers.

[26] Welch B.D., Yuan P., Bose S., Kors C.A., Lamb R.A., Jardetzky T.S. (2013). Structure of the parainfluenza virus 5 (PIV5) hemagglutinin-neuraminidase (HN) ectodomain. PLoS Pathog..

[27] Windhaber S., Xin Q., Lozach P.Y. (2021). Orthobunyaviruses: From Virus Binding to Penetration into Mammalian Host Cells. Viruses.

[28] Lee I., Bos S., Li G., Wang S., Gadea G., Despres P., Zhao R.Y. (2018). Probing Molecular Insights into Zika Virus–Host Interactions. Viruses.

[29] Saunders N., Fernandez I., Planchais C., Michel V., Rajah M.M., Baquero Salazar E., Postal J., Porrot F., Guivel-Benhassine F., Blanc C. (2023). TMPRSS2 is a functional receptor for human coronavirus HKU1. Nature.

[30] Hollidge B.S., Nedelsky N.B., Salzano M.V., Fraser J.W., Gonzalez-Scarano F., Soldan S.S. (2012). Orthobunyavirus entry into neurons and other mammalian cells occurs via clathrin-mediated endocytosis and requires trafficking into early endosomes. J. Virol..

[31] Owczarek K., Szczepanski A., Milewska A., Baster Z., Rajfur Z., Sarna M., Pyrc K. (2018). Early events during human coronavirus OC43 entry to the cell. Sci. Rep..

[32] Zokarkar A., Lamb R.A. (2012). The paramyxovirus fusion protein C-terminal region: Mutagenesis indicates an indivisible protein unit. J. Virol..

[33] da Silva F.M.A., da Silva K.P.A., de Oliveira L.P.M., Costa E.V., Koolen H.H., Pinheiro M.L.B., de Souza A.Q.L., de Souza A.D.L. (2020). Flavonoid glycosides and their putative human metabolites as potential inhibitors of the SARS-CoV-2 main protease (Mpro) and RNA-dependent RNA polymerase (RdRp). Mem. Inst. Oswaldo Cruz.

[34] Duran N., Polat M.F., Aktas D.A., Alagoz M.A., Ay E., Cimen F., Tek E., Anil B., Burmaoglu S., Algul O. (2021). New chalcone derivatives as effective against SARS-CoV-2 agent. Int. J. Clin. Pract..

[35] Venkataraman S., Prasad B., Selvarajan R. (2018). RNA Dependent RNA Polymerases: Insights from Structure, Function and Evolution. Viruses.

[36] Xiao X.-Y., Hao M., Yang X.-Y., Ba Q., Li M., Ni S.-J., Wang L.-S., Du X. (2011). Licochalcone A inhibits growth of gastric cancer cells by arresting cell cycle progression and inducing apoptosis. Cancer Lett..

[37] Lipinski C.A., Lombardo F., Dominy B.W., Feeney P.J. (2001). Experimental and computational approaches to estimate solubility and permeability in drug discovery and development settings. Adv. Drug Deliv. Rev..

[38] Benet L.Z., Hosey C.M., Ursu O., Oprea T.I. (2016). BDDCS, the Rule of 5 and drugability. Adv. Drug Deliv. Rev..

[39] Patel D., De R., Azadi N., Lee S., Shooter S., Amichai S., Zhou S., Monroe D., Mahanke C., McBrayer T.R. (2025). Discovery of broad-spectrum antivirals targeting viral proteases using in silico structural modeling and cellular analysis. Antivir. Res..

[40] Luong Q.X.T., Hoang P.T., Ho P.T., Ayun R.Q., Lee T.K., Lee S. (2025). Potential Broad-Spectrum Antiviral Agents: A Key Arsenal Against Newly Emerging and Reemerging Respiratory RNA Viruses. Int. J. Mol. Sci..

[41] Eddine F.Z.L., Mathez G., Carlen V., Dolci I., Fernandes R.S., Godoy A.S., Laleu B., Cagno V. (2025). Identification of pan-flavivirus compounds from drug repurposing. Antivir. Res..

