# Supplementary material for: Synthesis and Bioevaluation of Chalcones as Broad-Spectrum Antiviral Compounds Against Single-Stranded RNA Viruses
Source: Biomolecules. 2025 Sep 5;15(9):1285. doi: 10.3390/biom15091285 (PMC12467382; doi:10.3390/biom15091285)
Supplement: Supplementary file 1 [file biomolecules-15-01285-s001.zip › biomolecules-3820480-supplementary.pdf]

Supplementary Materials

Synthesis and bioevaluation of chalcones as broad-spectrum antiviral compounds against single-stranded RNA viruses

Lorael K.M. Kirton [1, †], Nasser N. Yousef [2, †], Griffith D. Parks [2] and Otto Phanstiel IV [1, *]

[1]12722 Research Parkway, College of Medicine, University of Central Florida, Orlando, Florida 32826, United States

[2]6900 Lake Nona Blvd, College of Medicine, Burnett School of Biomedical Sciences, University of Central Florida, Orlando, FL 32827, United States

[†] Joint first authors

* Communicating Author
Contact Information:
12722 Research Parkway
College of Medicine
University of Central Florida,
Orlando, FL 32826
(407) 823-6545
Otto.Phanstiel@ucf.edu

Table of Contents **page**

^{1}H NMR spectrum for compound 6b	S3
^{1}H NMR and ^{13}C NMR spectra for compound 7a	S4
^{1}H NMR and ^{13}C NMR spectra for compound 7b	S5
^{1}H NMR and ^{13}C NMR spectra for compound 7c	S6
^{1}H NMR and ^{13}C NMR spectra for compound 7d	S7
Mass spectrum for compound 7d	S8
^{1}H NMR and ^{13}C NMR spectra for compound 8q	S9
Mass spectrum for compound 8q	S10
^{1}H NMR and ^{13}C NMR spectra for compound 8r	S11
Mass spectrum for compound 8r	S12
^{1}H NMR and ^{13}C NMR spectra for compound 8s	S13
Mass spectrum for compound 8s	S14
^{1}H NMR and ^{13}C NMR spectra for compound 8t	S15
Mass spectrum for compound 8t	S16
^{1}H NMR and ^{13}C NMR spectra for compound 8u	S17
Mass spectrum for compound 8u	S18
^{1}H NMR and ^{13}C NMR spectra for compound 8v	S19
Mass spectrum for compound 8v	S20
^{1}H NMR and ^{13}C NMR spectra for compound 8w	S21
Mass spectrum for compound 8w	S22
^{1}H NMR and ^{13}C NMR spectra for compound 8x	S23
Mass spectrum and HPLC for compound 8x	S24
^{1}H NMR and ^{13}C NMR spectra for compound 8y	S25
Mass spectrum and HPLC for compound 8y	S26
^{1}H NMR and ^{13}C NMR spectra for compound 8z	S27
Mass spectrum and HPLC for compound 8z	S28
^{1}H NMR and ^{13}C NMR spectra for compound NM5	S29
Mass spectrum for compound NM5	S30
Table S1. Elemental Analyses for 7d, 8q-8w and NM5	S31

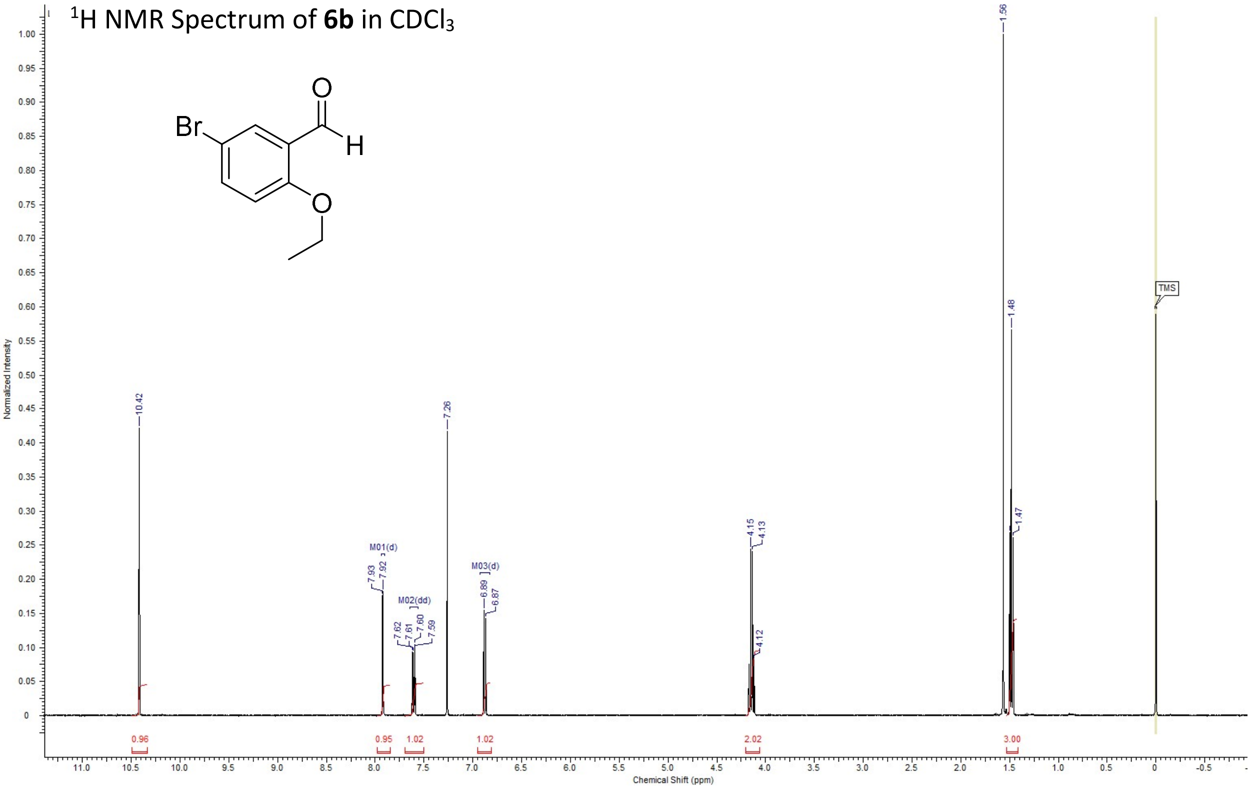
1H NMR Spectrum of 6b in CDCl3
Br
O
H
O
Normalized Intensity
Chemical Shift (ppm)
10.42
7.93
7.92
M01(d)
M02(dd)
7.62
7.61
7.60
7.59
7.26
M03(d)
6.89
6.87
4.15
4.13
4.12
1.56
1.48
1.47
TMS
0.96
0.95
1.02
1.02
2.02
3.00

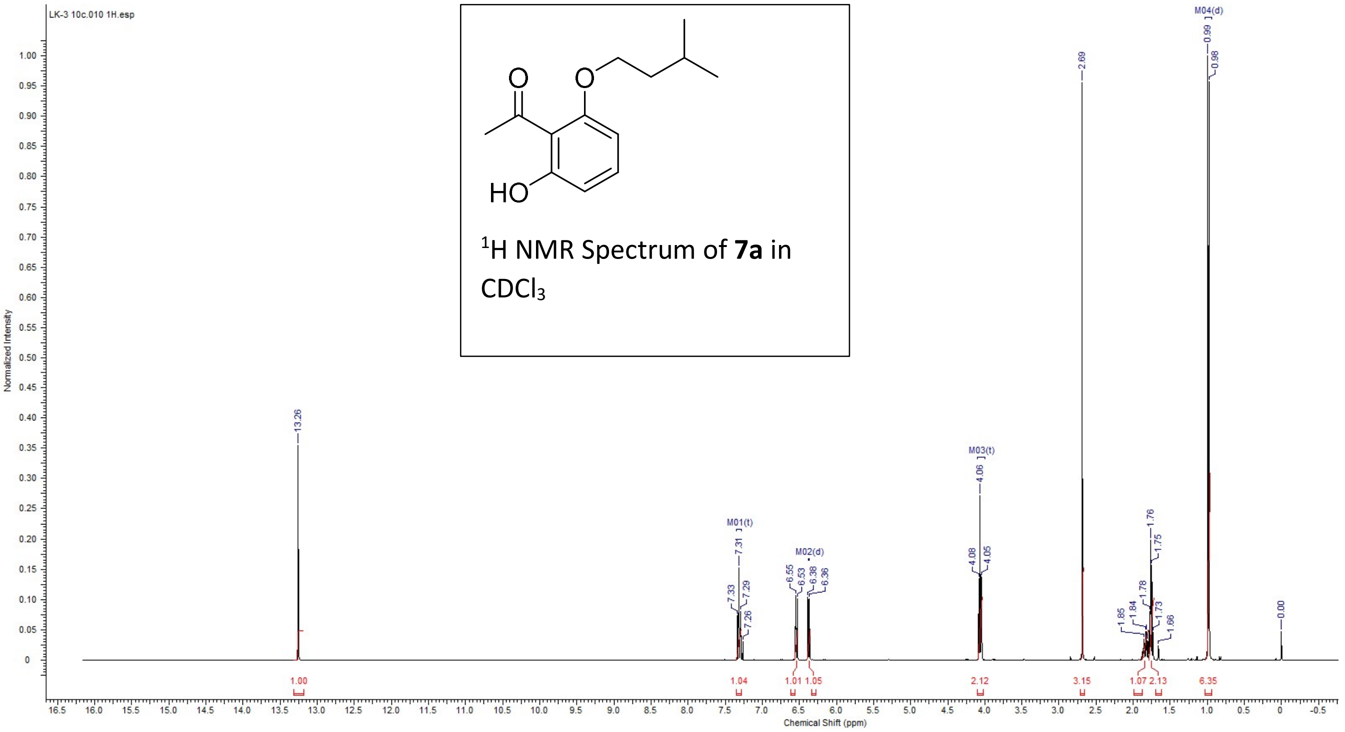
LK-3 10c.010 1H.esp
^{1}H NMR Spectrum of **7a** in CDCl$_3$
O
O
HO
Normalized Intensity
Chemical Shift (ppm)
M01(t)
M02(d)
M03(t)
M04(d)

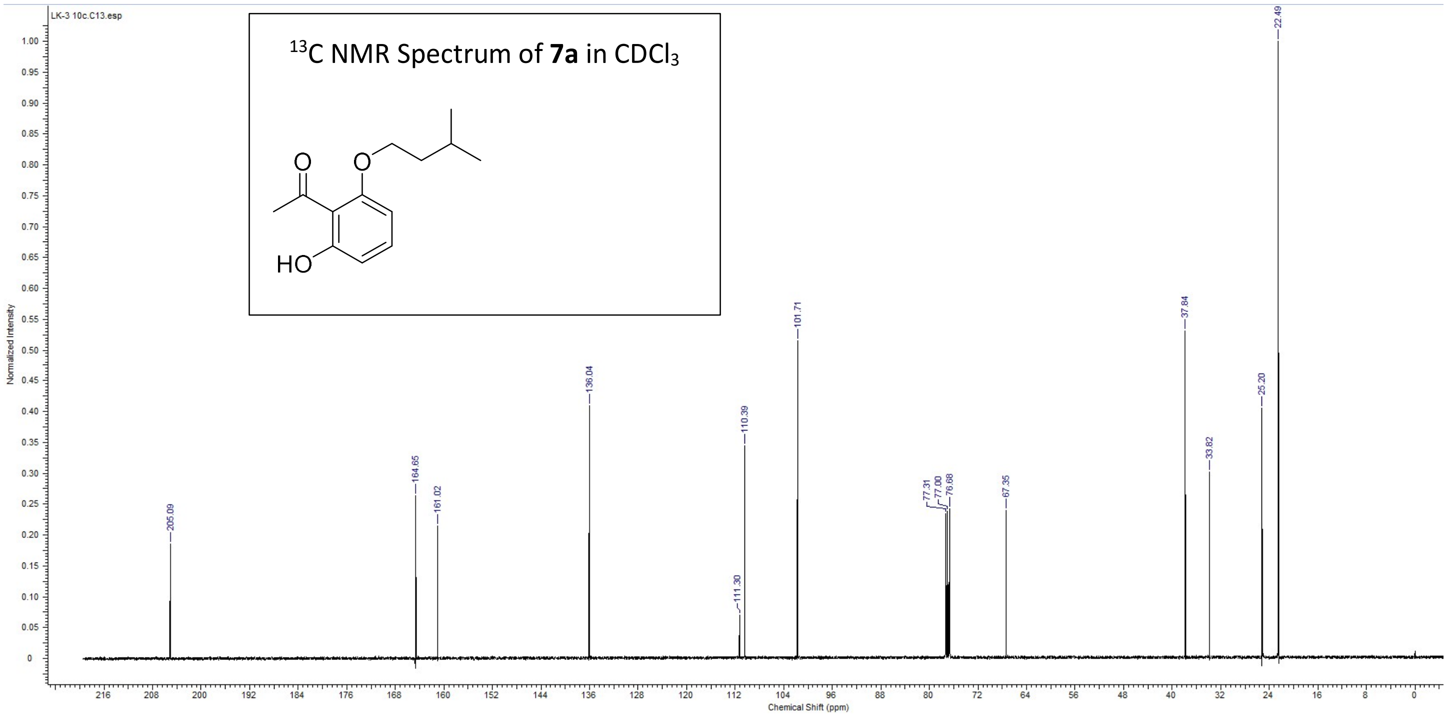
LK-3 10c.C13.esp
^{13}C NMR Spectrum of **7a** in CDCl$_3$
O
O
HO
Normalized Intensity
Chemical Shift (ppm)

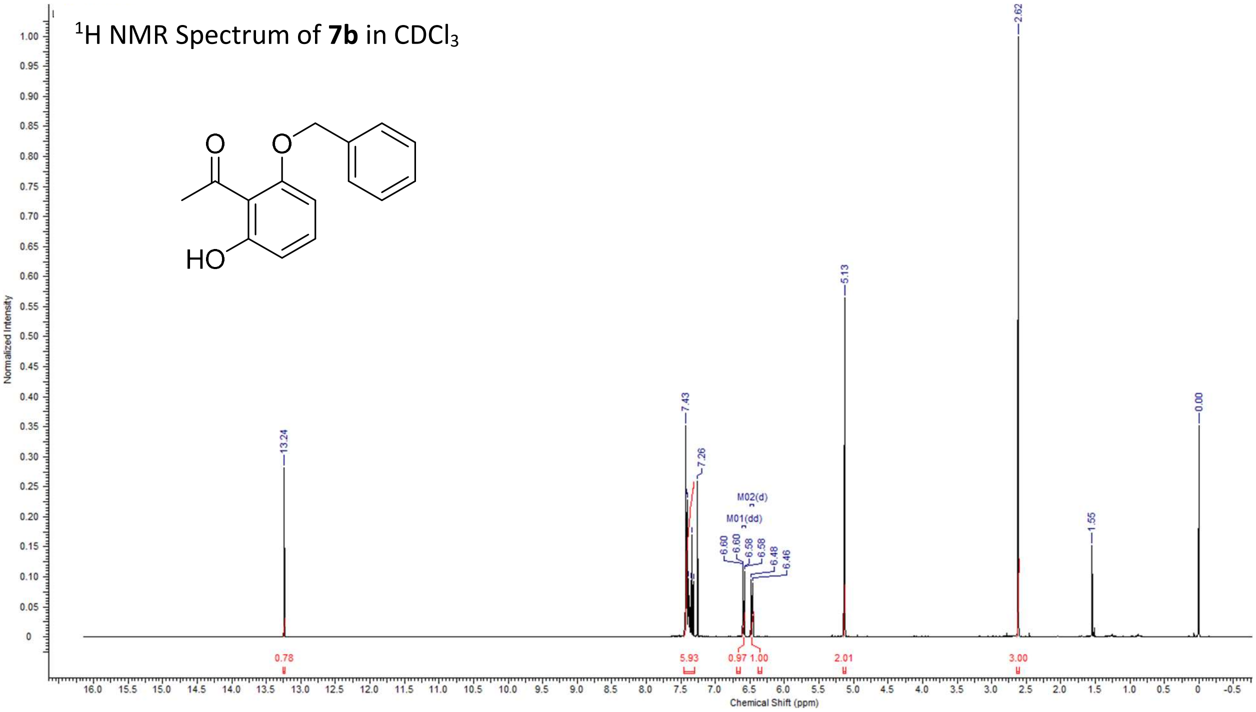

^{1}H NMR Spectrum of **7b** in $CDCl_3$
HO
O
O
13.24
7.43
7.26
M02(d)
M01(dd)
6.60
6.60
6.58
6.58
6.48
6.46
5.13
2.62
1.55
0.00
0.78
5.93
0.97
1.00
2.01
3.00
Normalized Intensity
Chemical Shift (ppm)

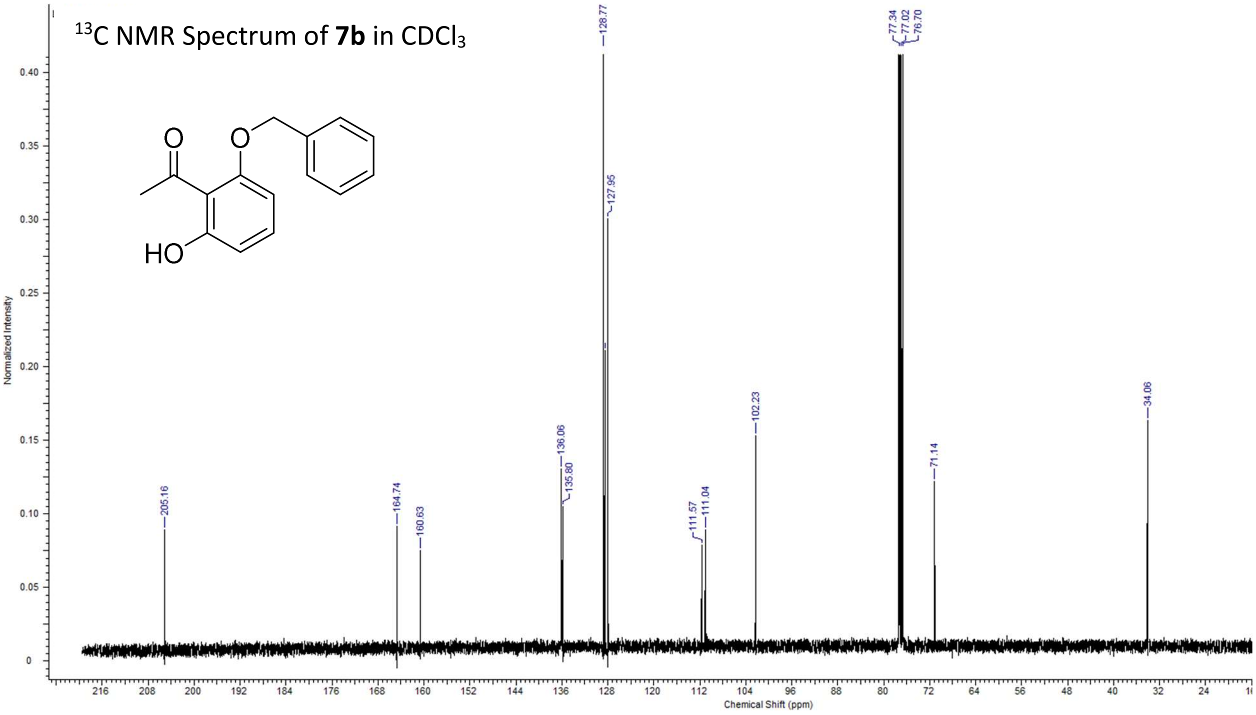

^{13}C NMR Spectrum of **7b** in $CDCl_3$
HO
O
O
205.16
164.74
160.63
136.06
135.80
128.77
127.95
111.57
111.04
102.23
77.34
77.02
76.70
71.14
34.06
Normalized Intensity
Chemical Shift (ppm)

^{1}H NMR Spectrum of **7c** in $CDCl_3$

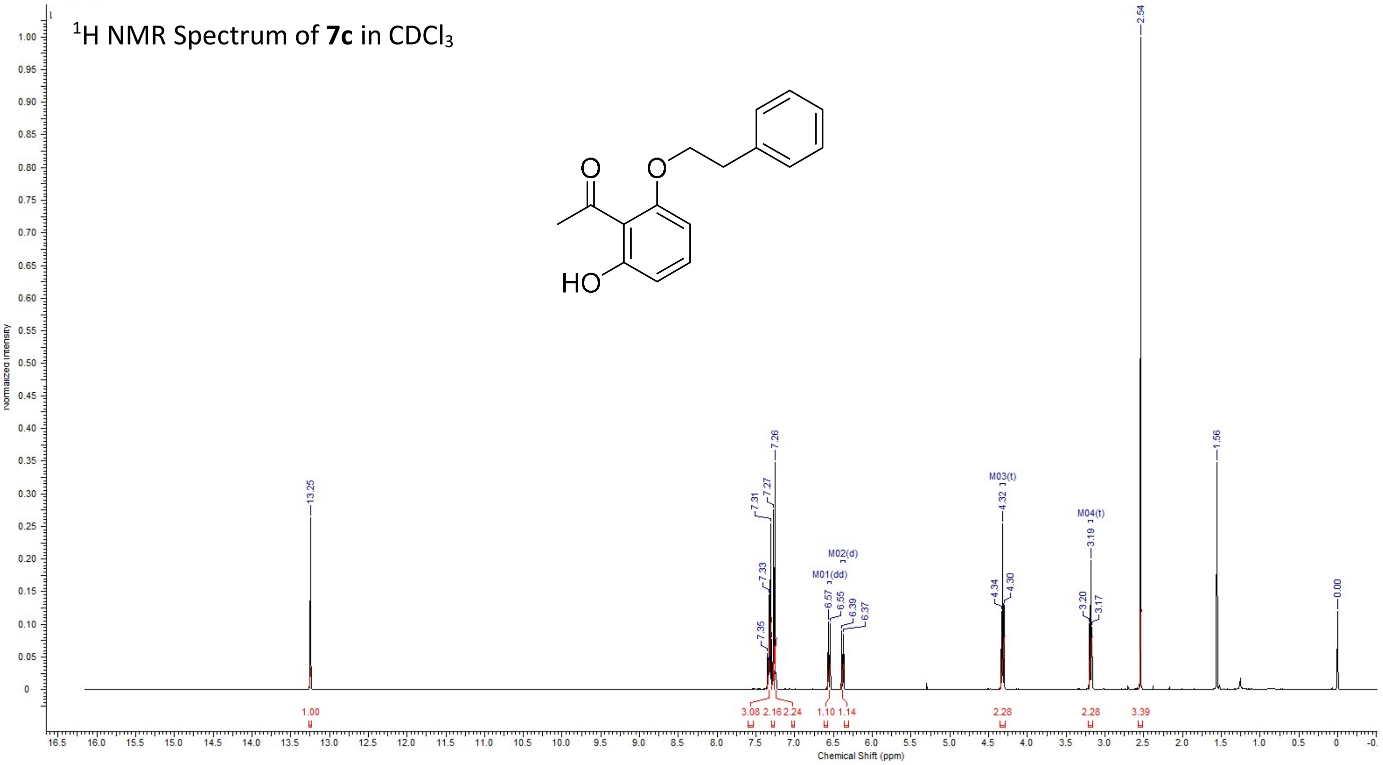

^{13}C NMR Spectrum of **7c** in $CDCl_3$

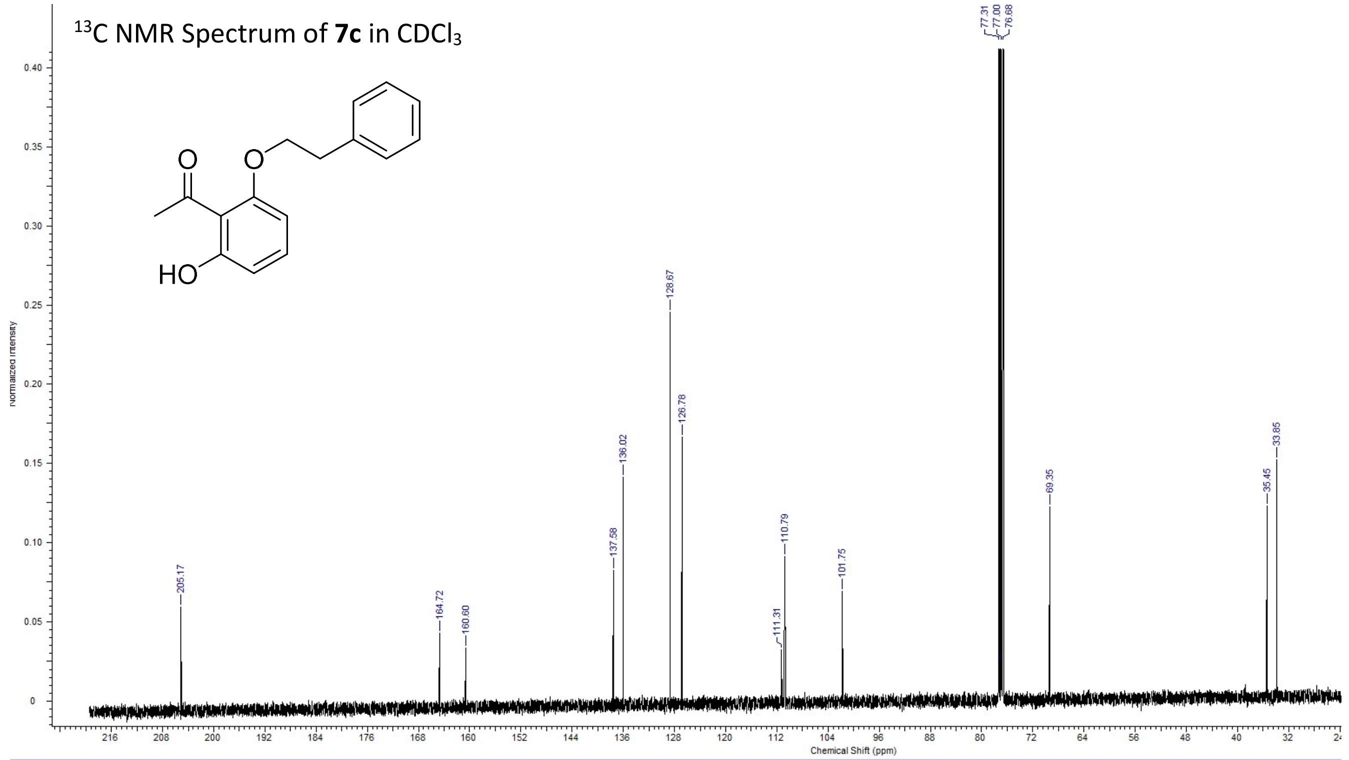

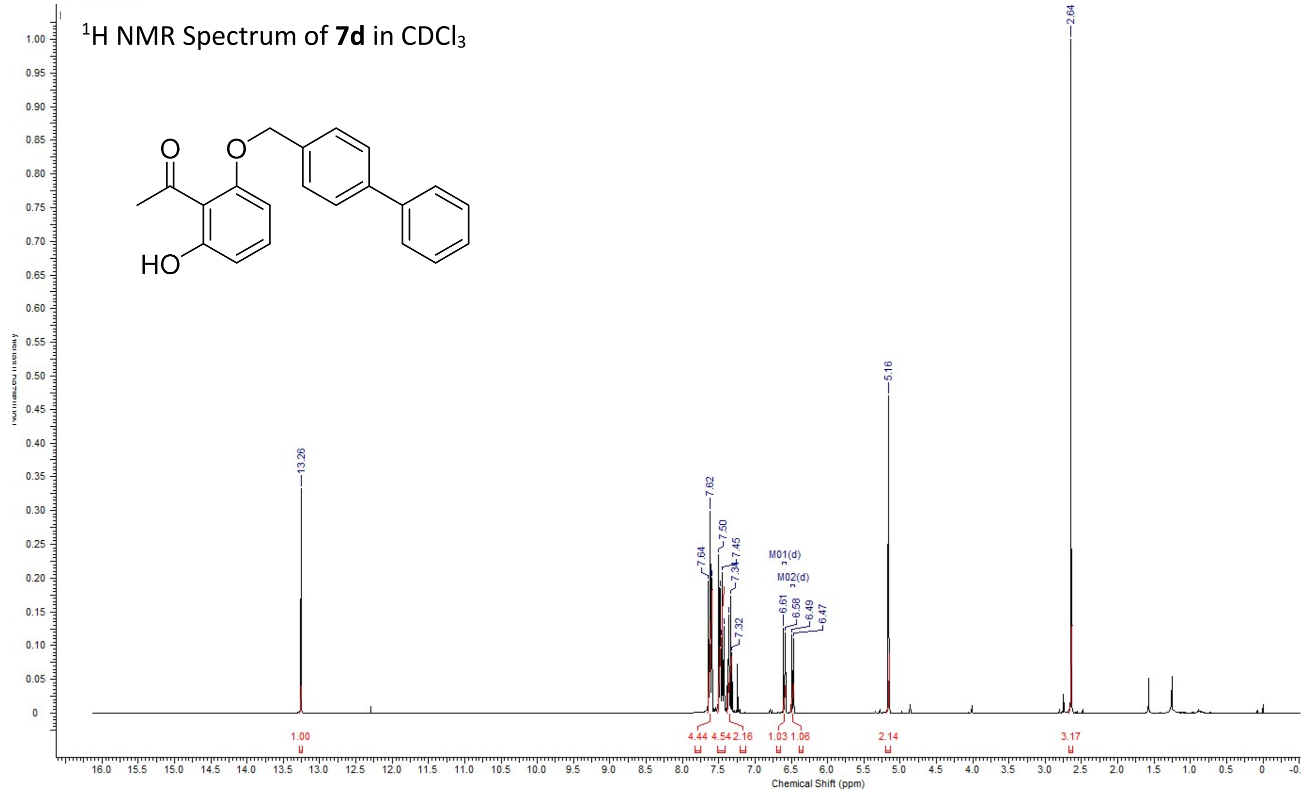

^{1}H NMR Spectrum of **7d** in $CDCl_3$
HO
O
O
13.26
7.64
7.62
7.50
7.45
7.34
7.32
M01(d)
M02(d)
6.61
6.58
6.49
6.47
5.16
2.64
1.00
4.44
4.54
2.16
1.03
1.06
2.14
3.17
Chemical Shift (ppm)

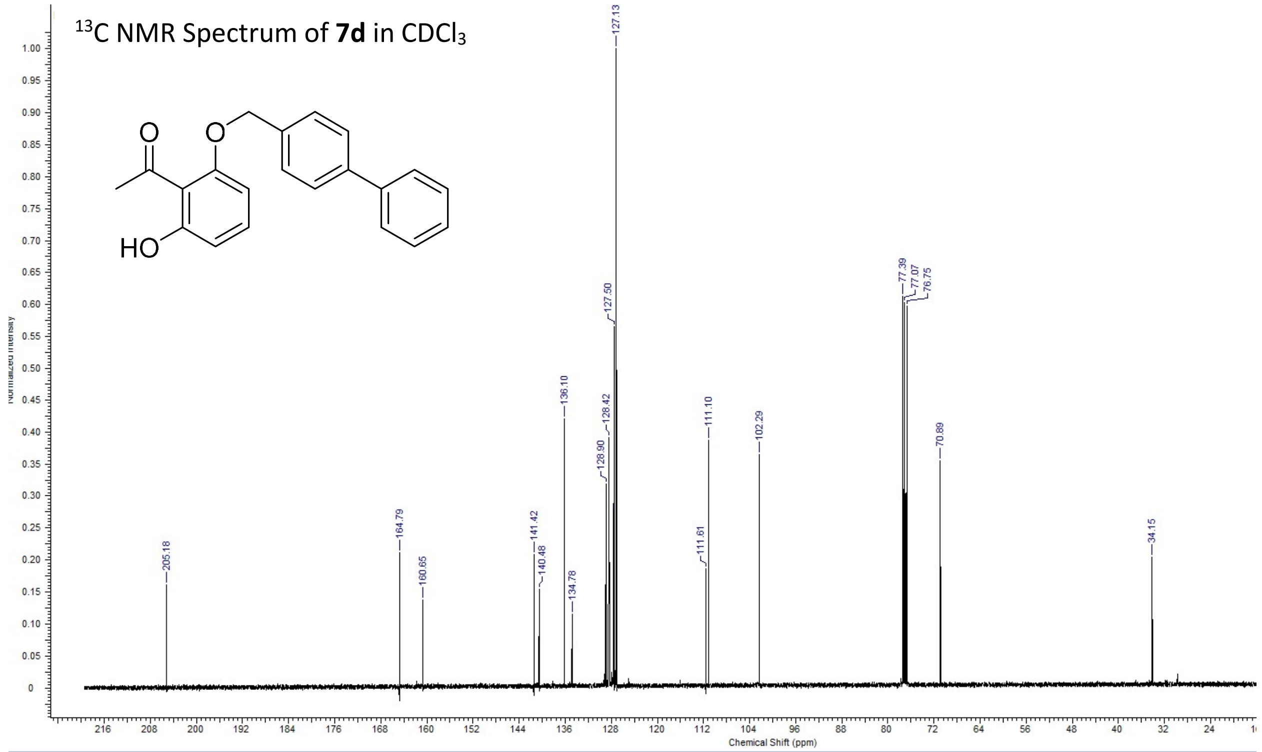

^{13}C NMR Spectrum of **7d** in $CDCl_3$
HO
O
O
205.18
164.79
160.65
141.42
140.48
136.10
134.78
128.90
128.42
127.50
127.13
111.61
111.10
102.29
77.39
77.07
76.75
70.89
34.15
Chemical Shift (ppm)

Mass Spectrum of **7d**

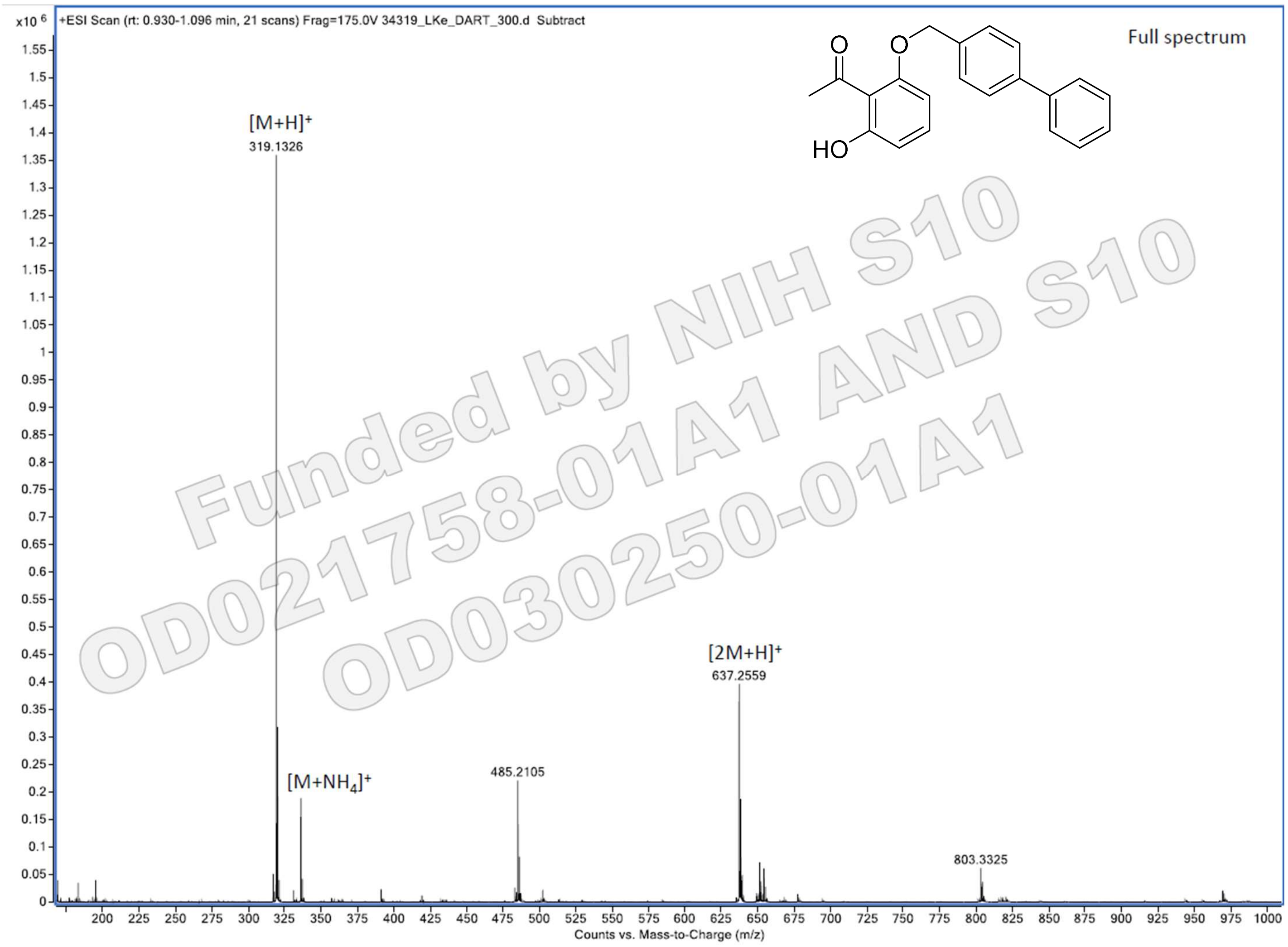

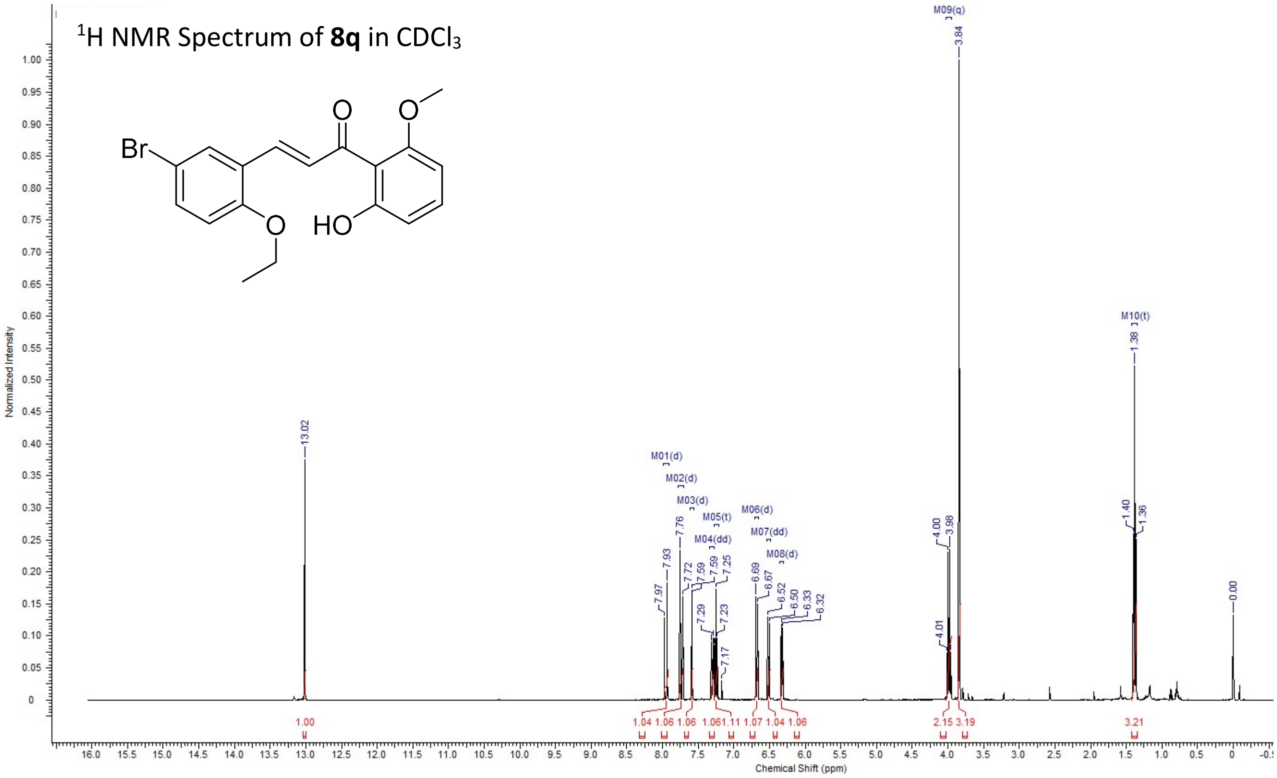

^{1}H NMR Spectrum of **8q** in $CDCl_3$
Br
O
O
O
HO
Normalized Intensity
Chemical Shift (ppm)
M01(d)
M02(d)
M03(d)
M04(dd)
M05(t)
M06(d)
M07(dd)
M08(d)
M09(q)
M10(t)
13.02
7.97
7.93
7.76
7.72
7.59
7.59
7.29
7.25
7.23
7.17
6.69
6.67
6.52
6.50
6.33
6.32
4.01
4.00
3.98
3.84
1.40
1.38
1.36
0.00
1.00
1.04
1.06
1.06
1.06
1.11
1.07
1.04
1.06
2.15
3.19
3.21

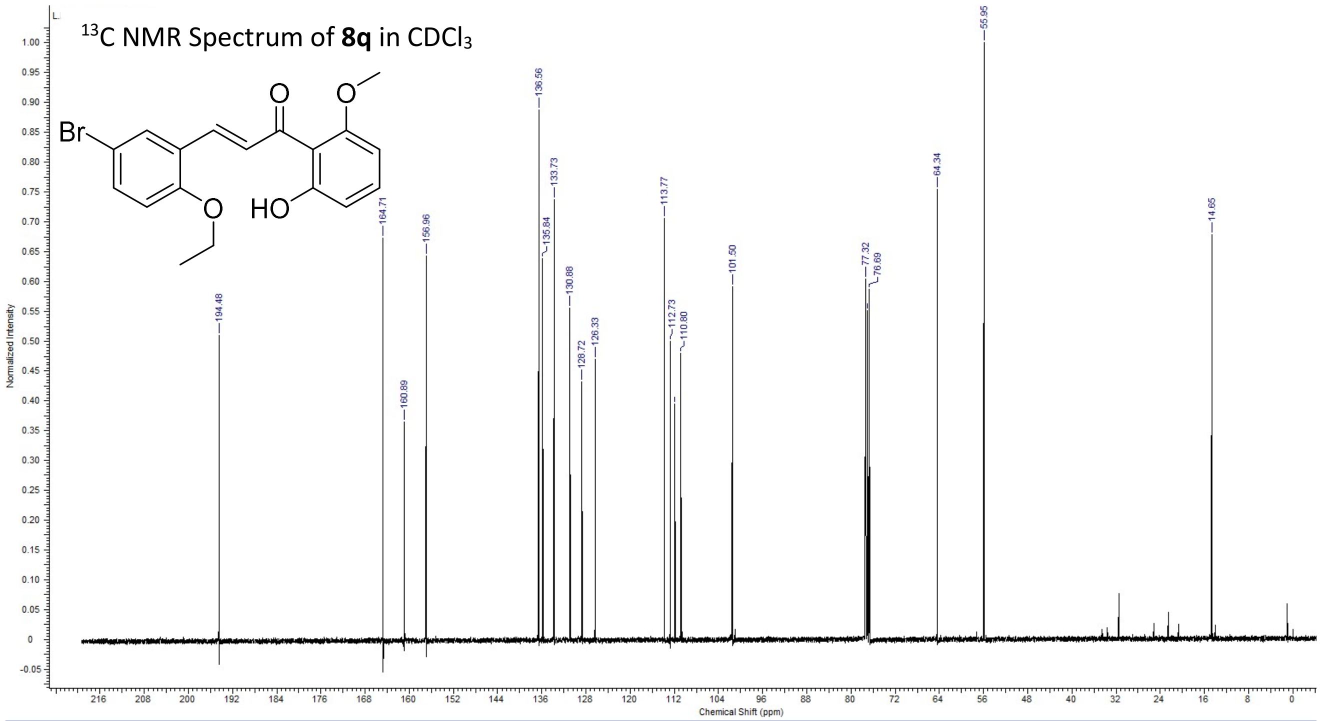

^{13}C NMR Spectrum of **8q** in $CDCl_3$
Br
O
O
O
HO
Normalized Intensity
Chemical Shift (ppm)
194.48
164.71
160.89
156.96
136.56
135.84
133.73
130.88
128.72
126.33
113.77
112.73
110.80
101.50
77.32
76.69
64.34
55.95
14.65

Mass Spectrum of **8q**

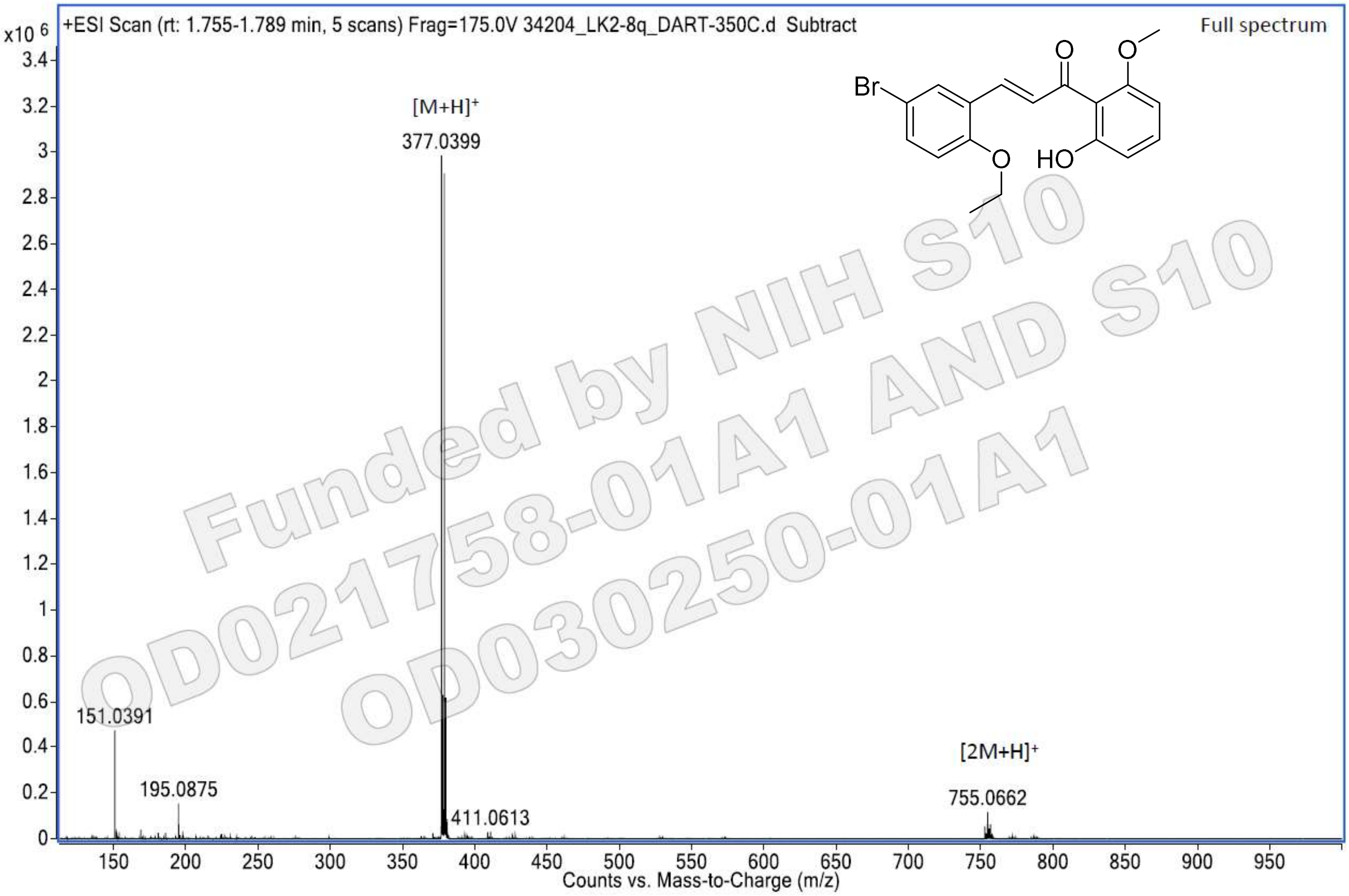

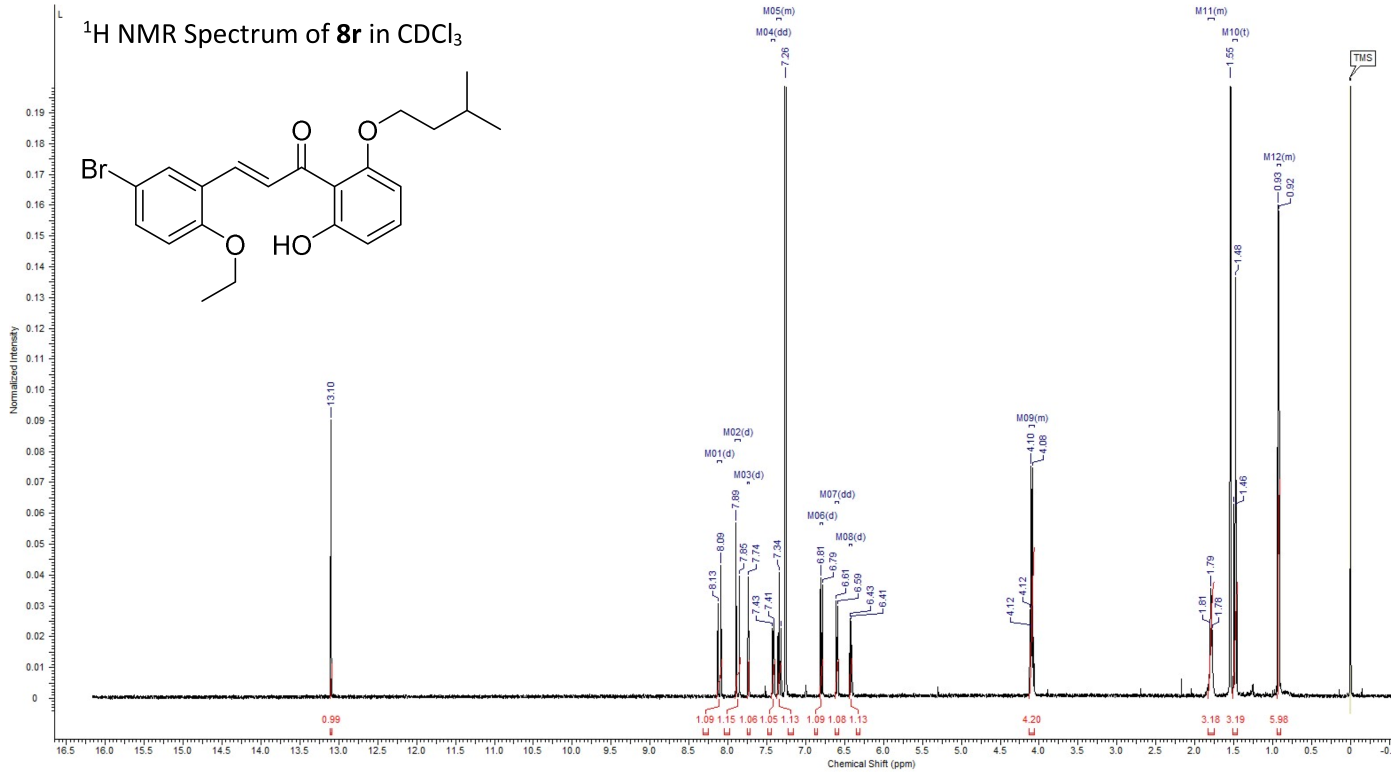

1H NMR Spectrum of 8r in CDCl3
Br
O
HO
Normalized Intensity
Chemical Shift (ppm)
TMS

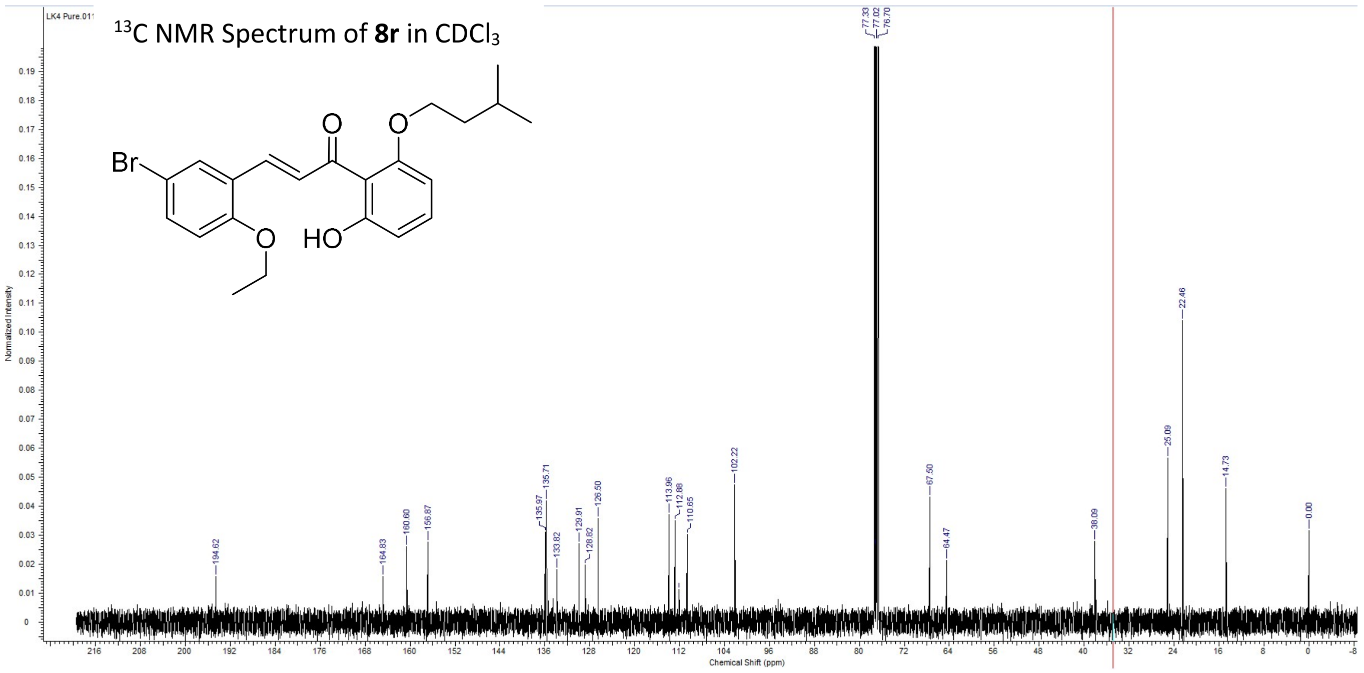

13C NMR Spectrum of 8r in CDCl3
LK4 Pure.011
Br
O
HO
Normalized Intensity
Chemical Shift (ppm)

Mass Spectrum of **8r**

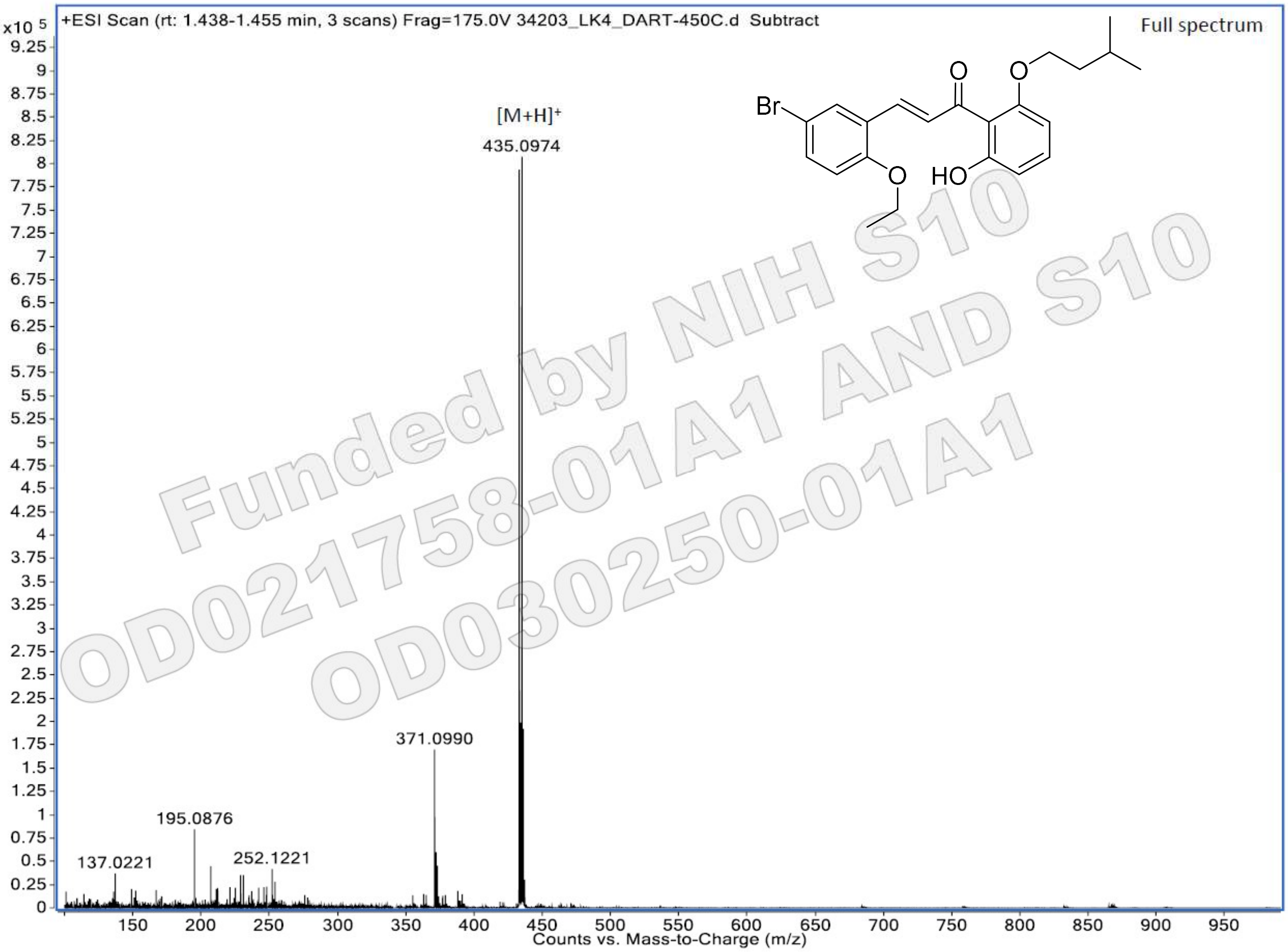

Funded by NIH S10 OD021758-01A1 AND S10 OD030250-01A1

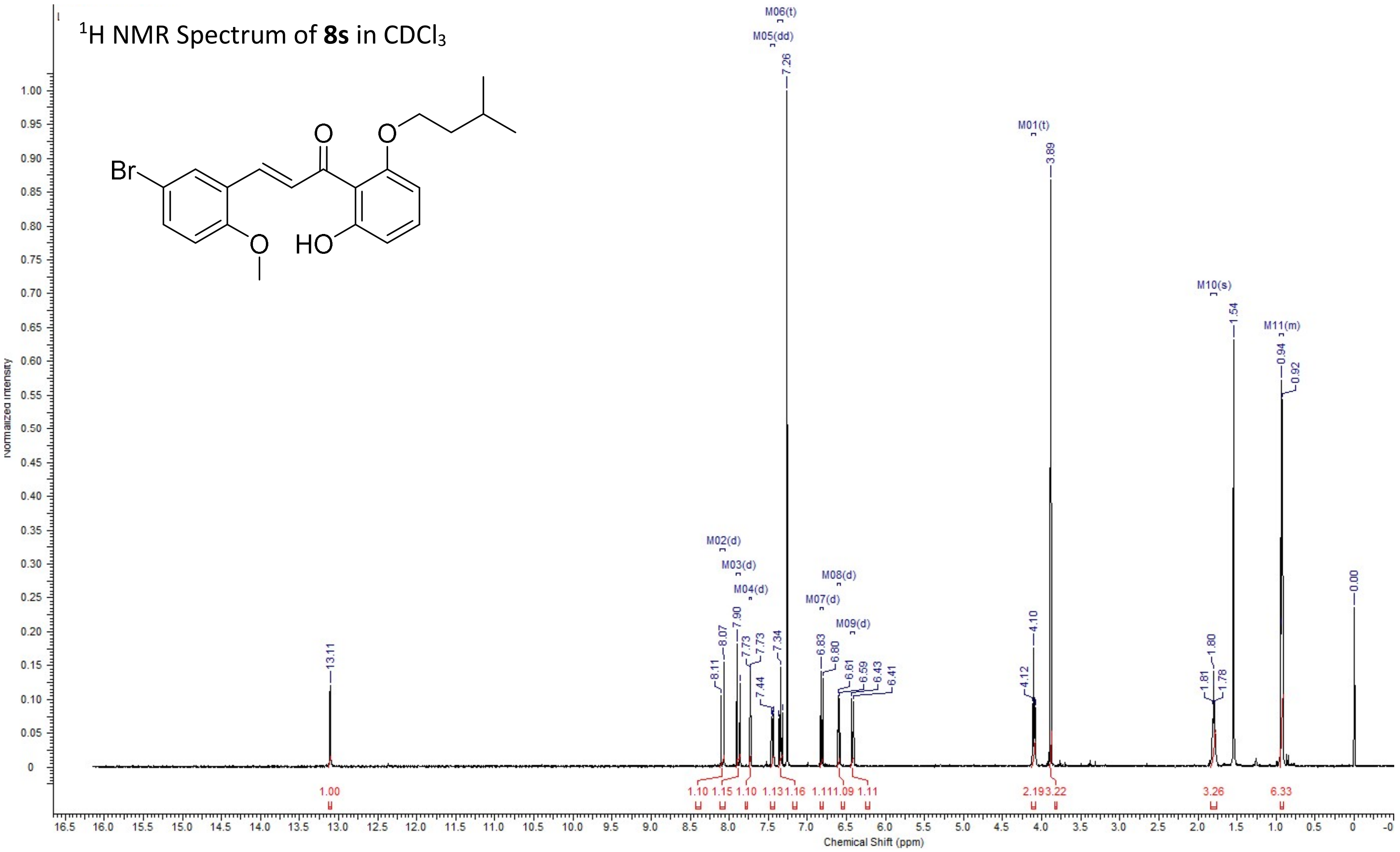

^{1}H NMR Spectrum of **8s** in CDCl$_3$
Br
O
O
HO
O
Normalized Intensity
Chemical Shift (ppm)

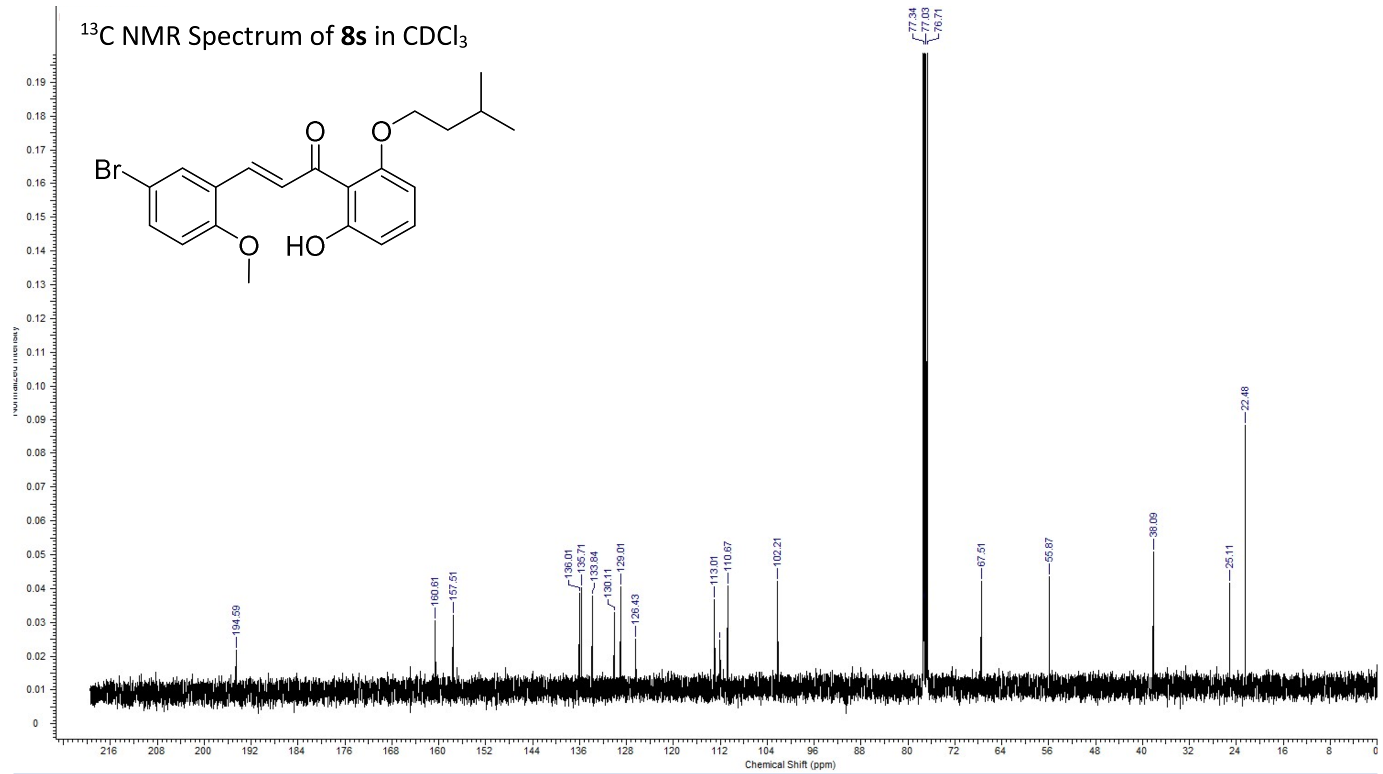

^{13}C NMR Spectrum of **8s** in CDCl$_3$
Br
O
O
HO
O
Chemical Shift (ppm)

Mass Spectrum of **8s**

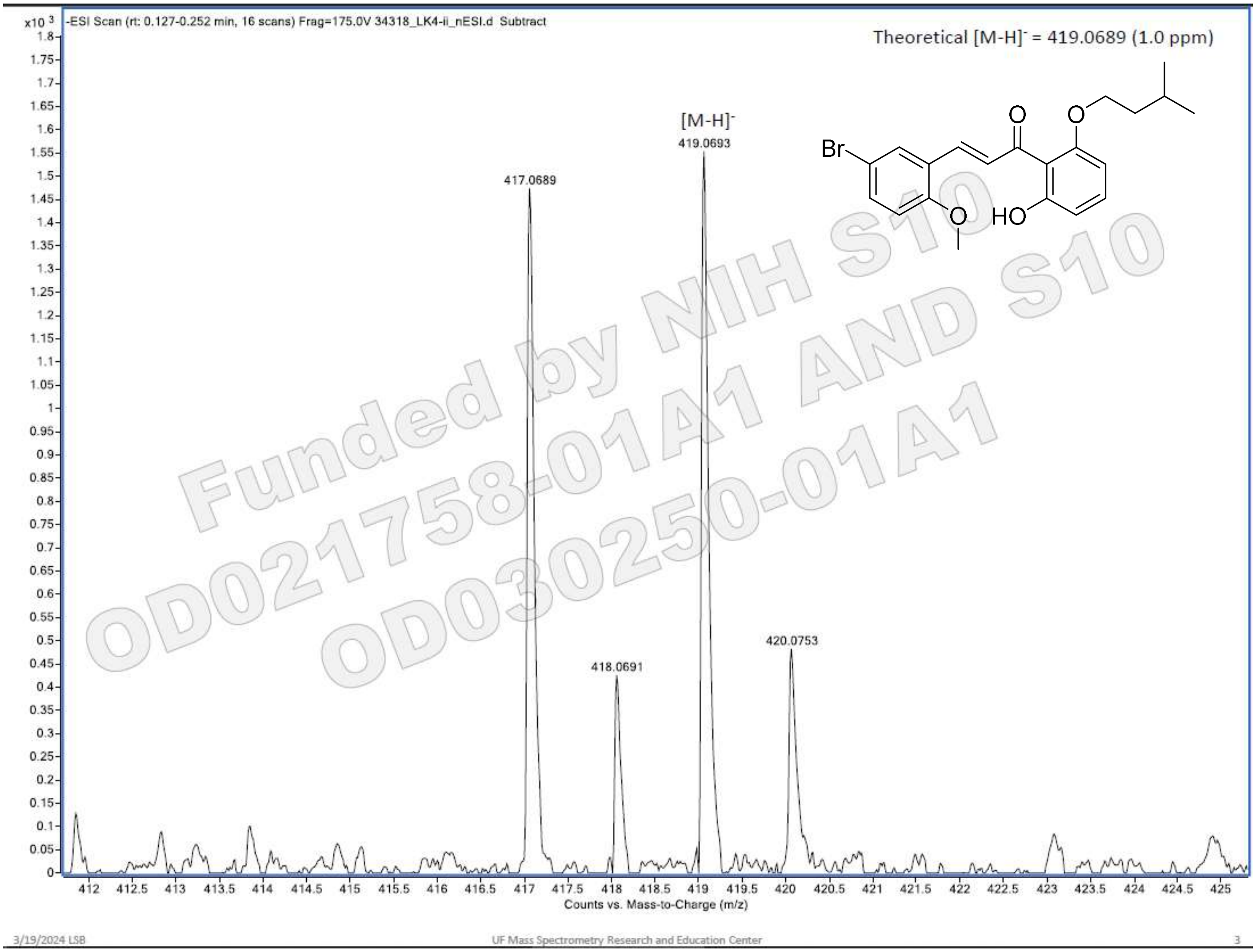

Funded by NIH S10 OD021758-01A1 AND S10 OD030250-01A1

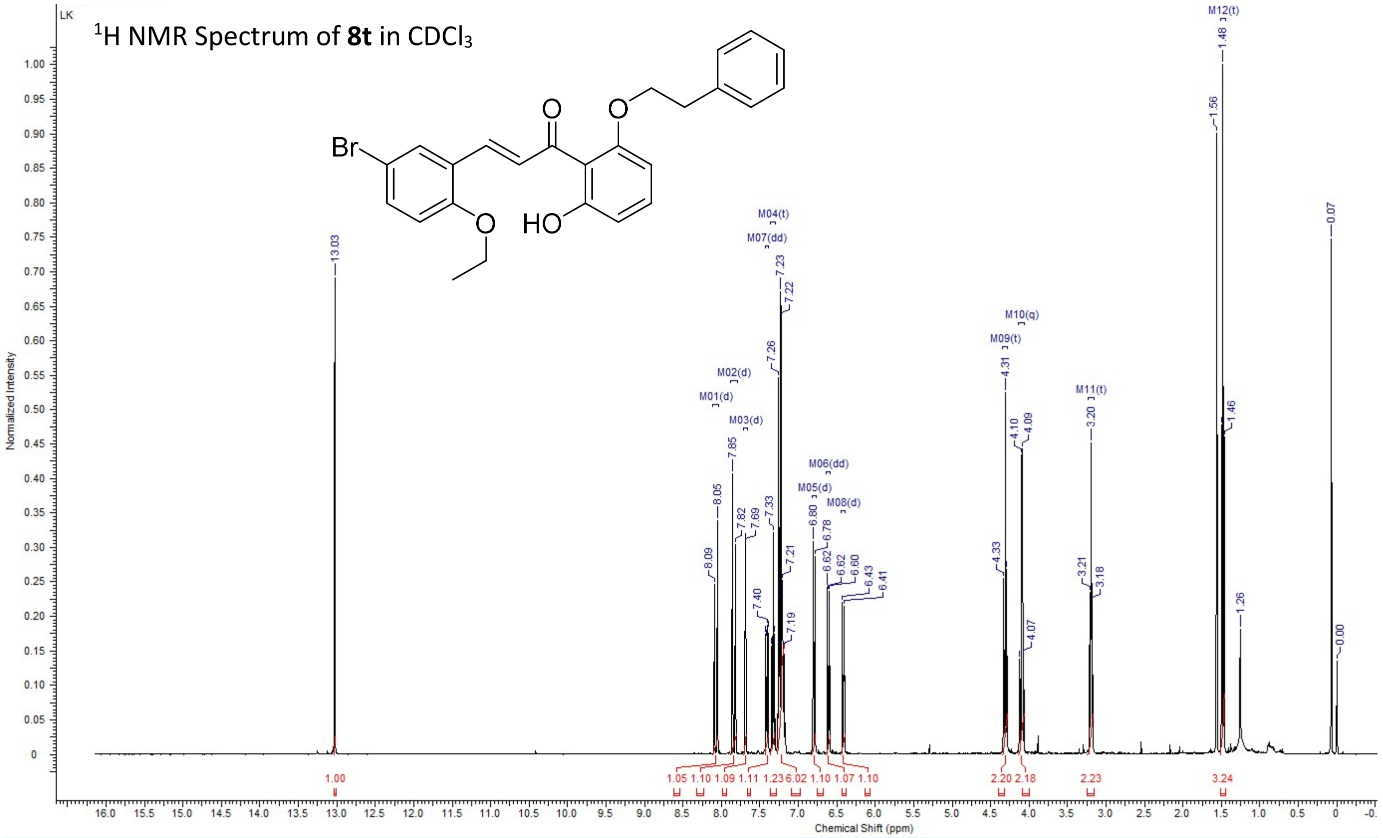

^{1}H NMR Spectrum of **8t** in $CDCl_3$
Br
O
O
HO
O
Chemical Shift (ppm)
Normalized Intensity

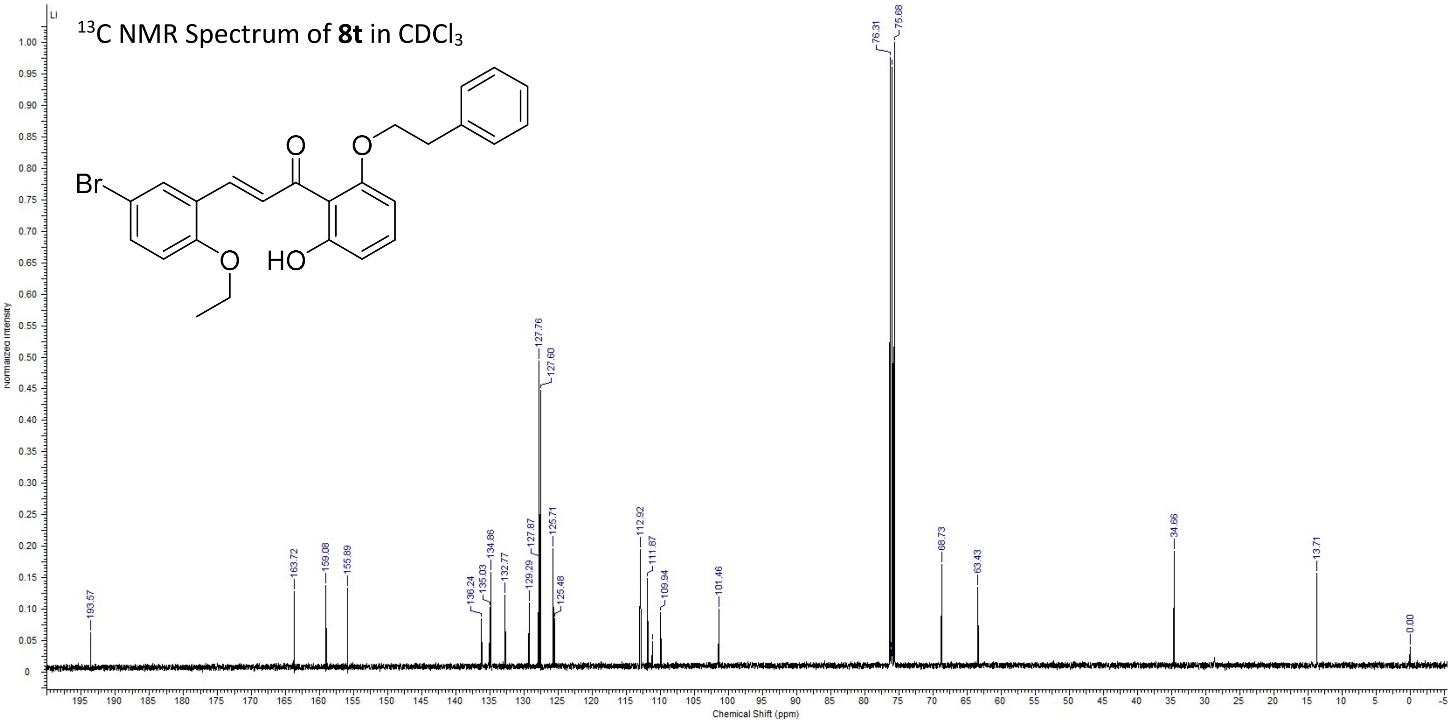

^{13}C NMR Spectrum of **8t** in $CDCl_3$
Br
O
O
HO
O
Chemical Shift (ppm)

Mass Spectrum of **8t**

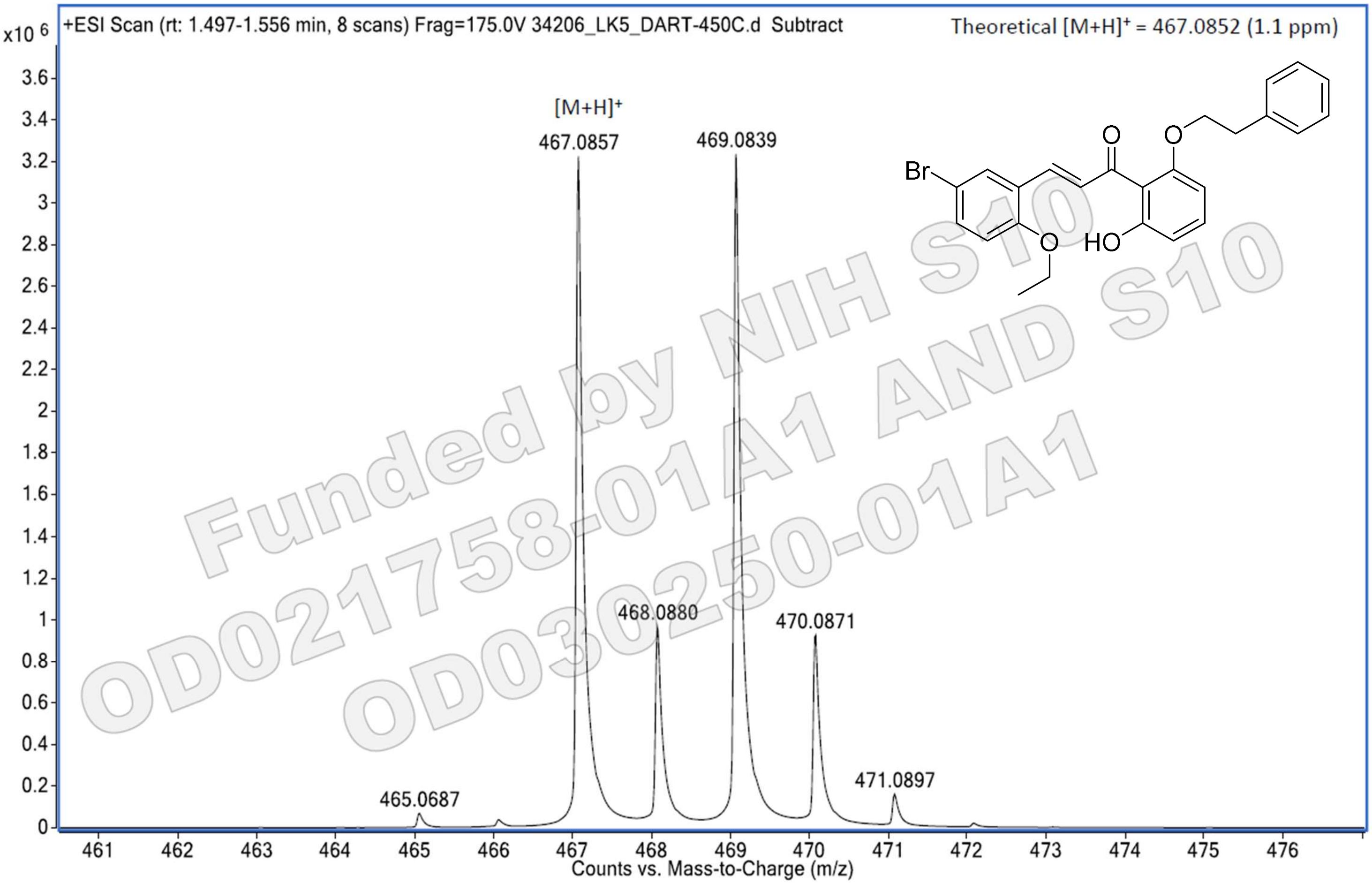

Funded by NIH S10 OD021758-01A1 AND S10 OD030250-01A1

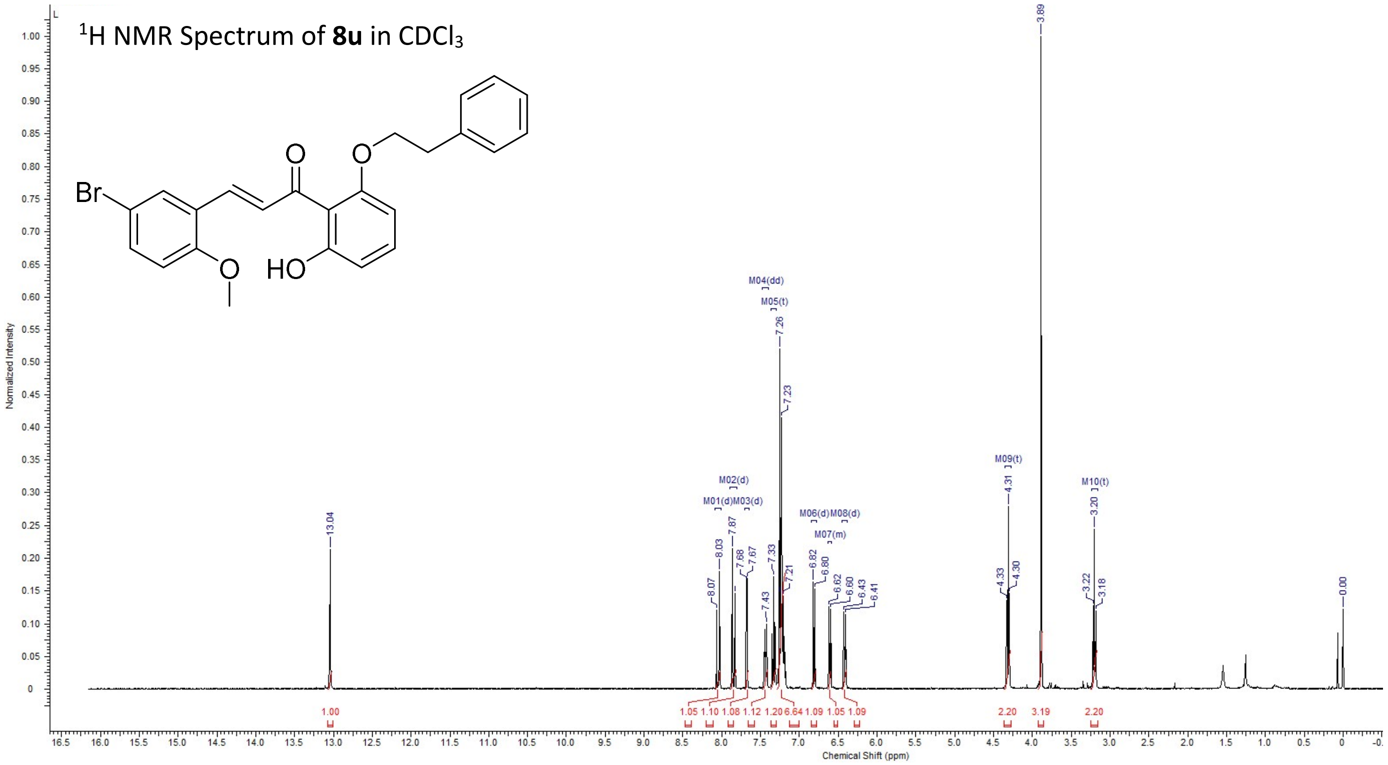
^{1}H NMR Spectrum of 8u in CDCl3
Br
O
O
HO
Normalized Intensity
Chemical Shift (ppm)
13.04
8.07
8.03
7.87
7.68
7.67
7.43
7.33
7.26
7.23
7.21
6.82
6.80
6.62
6.60
6.43
6.41
4.33
4.31
4.30
3.89
3.22
3.20
3.18
0.00
M01(d)
M02(d)
M03(d)
M04(dd)
M05(t)
M06(d)
M07(m)
M08(d)
M09(t)
M10(t)
1.00
1.05
1.10
1.08
1.12
1.20
6.64
1.09
1.05
1.09
2.20
3.19
2.20

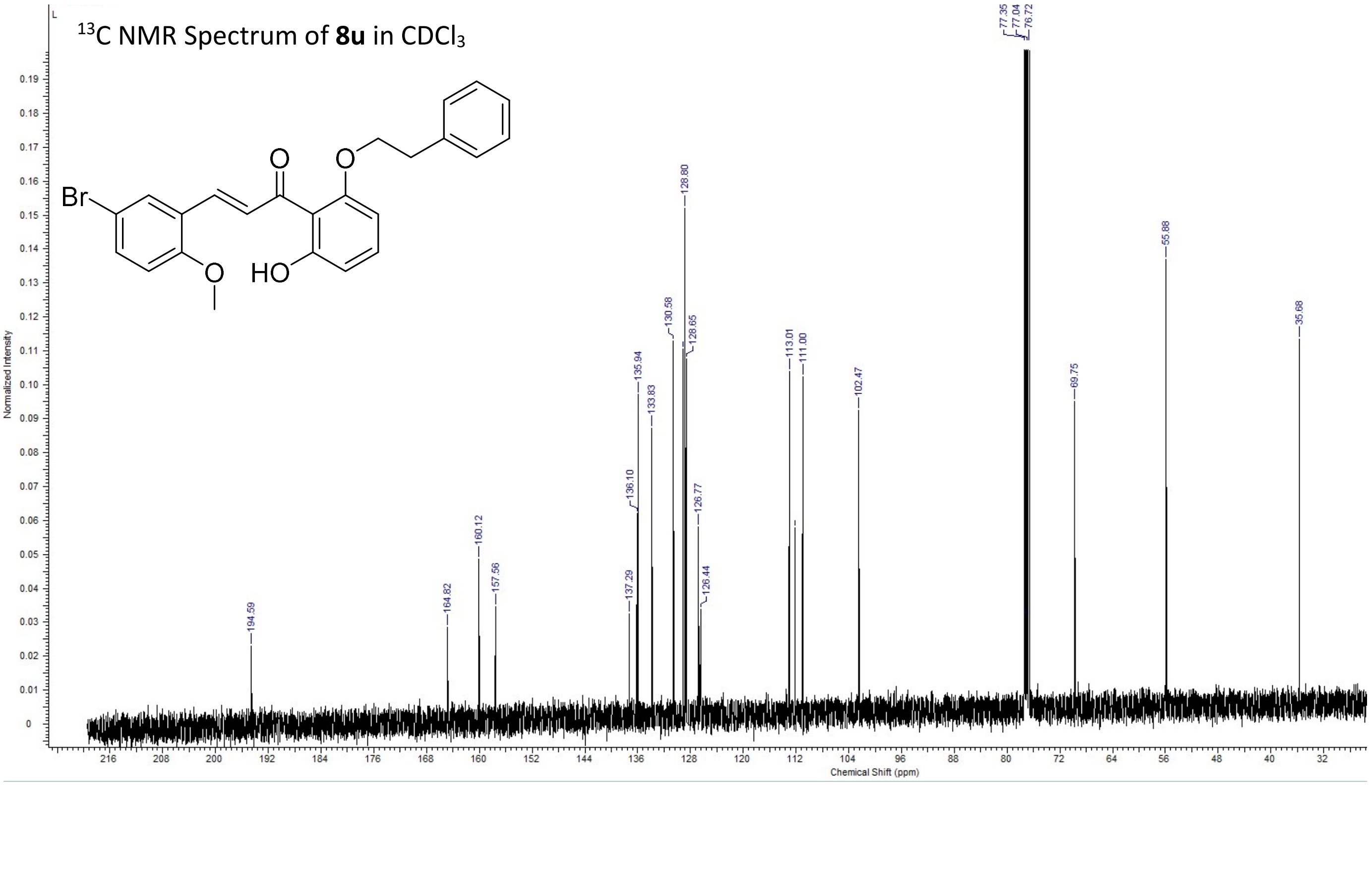
^{13}C NMR Spectrum of 8u in CDCl3
Br
O
O
HO
Normalized Intensity
Chemical Shift (ppm)
194.59
164.82
160.12
157.56
137.29
136.10
135.94
133.83
130.58
128.80
128.65
126.77
126.44
113.01
111.00
102.47
77.35
77.04
76.72
69.75
55.88
35.68

Mass Spectrum of **8u**

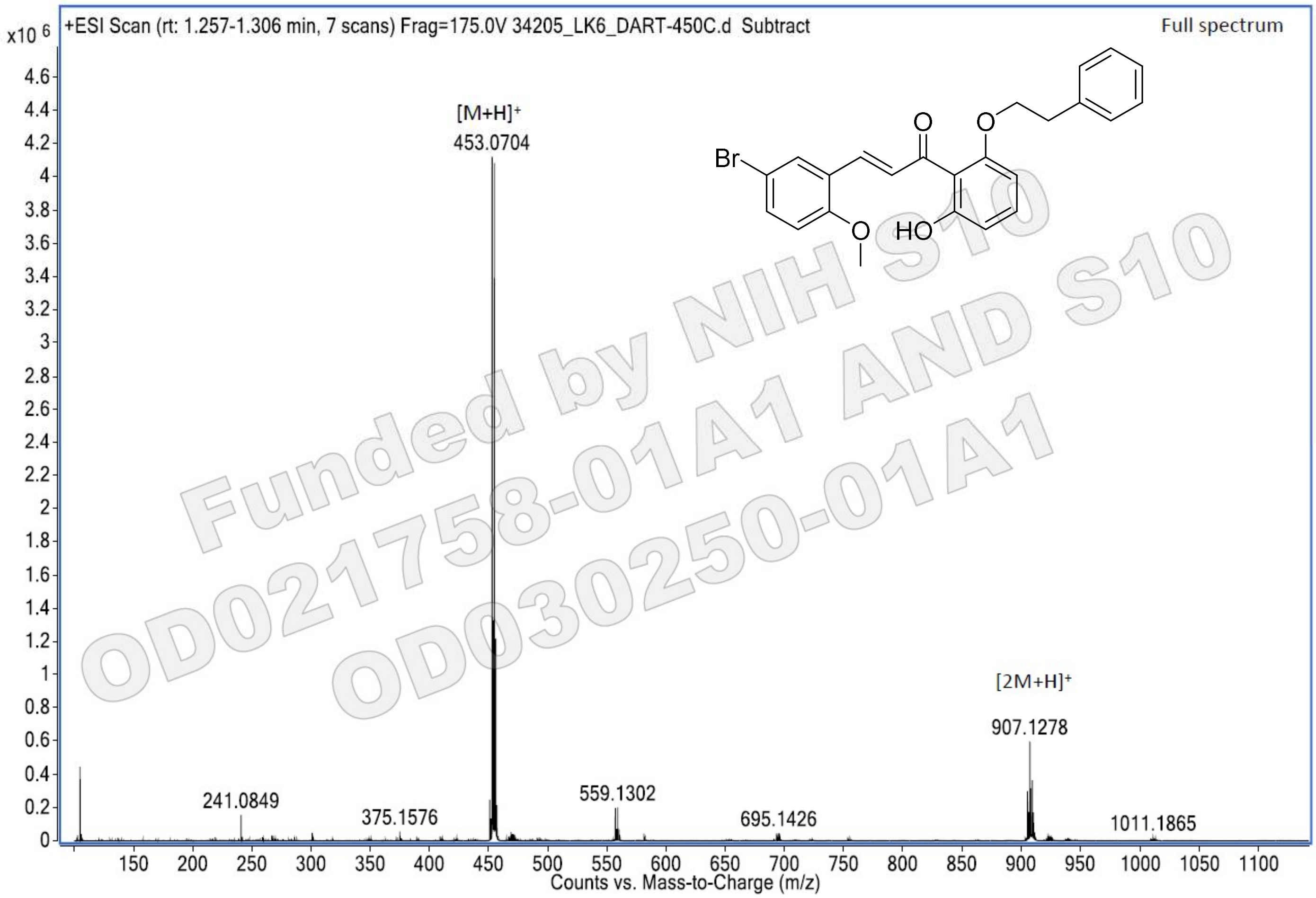

Funded by NIH S10 OD021758-01A1 AND S10 OD030250-01A1

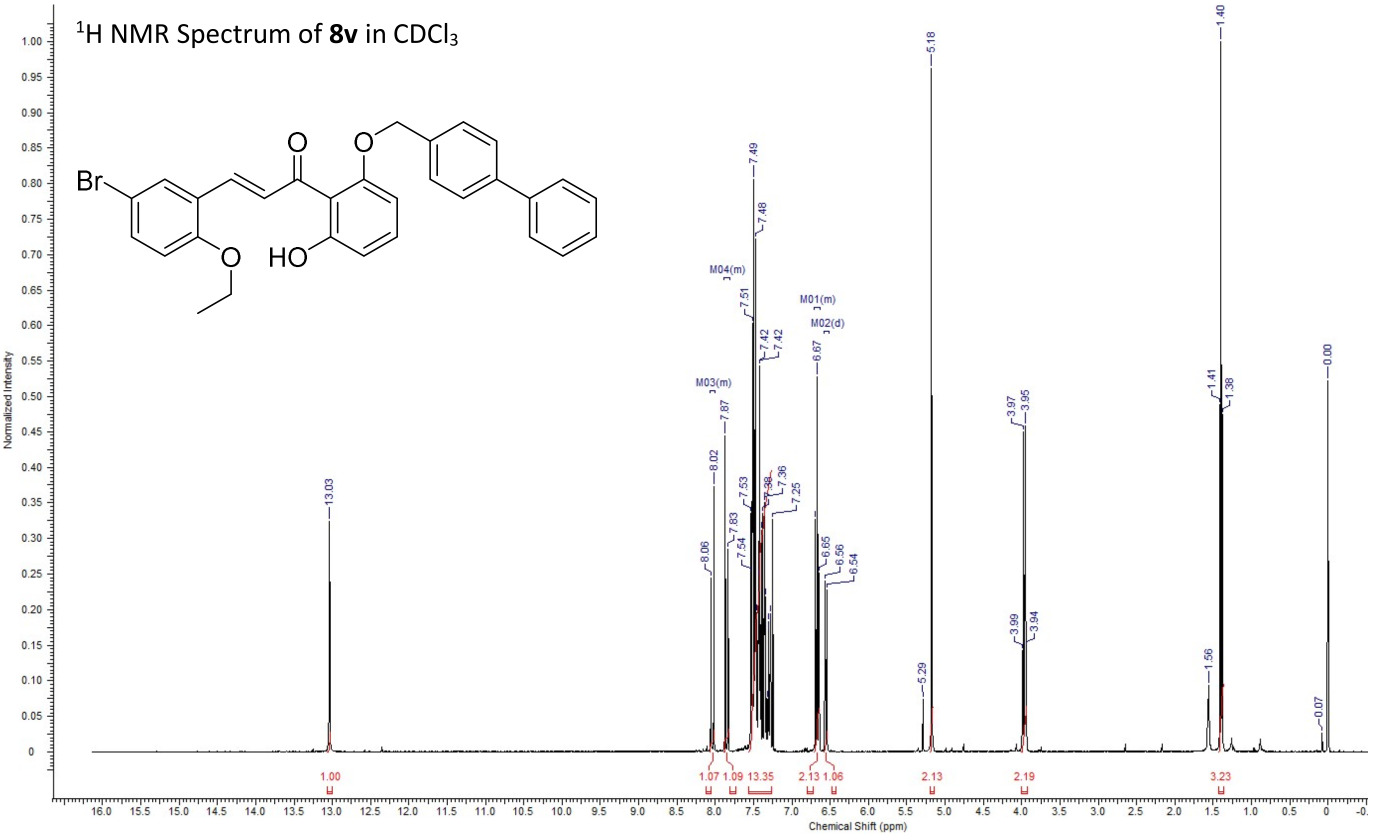

¹H NMR Spectrum of 8v in CDCl₃
Br
O
HO
Normalized Intensity
Chemical Shift (ppm)

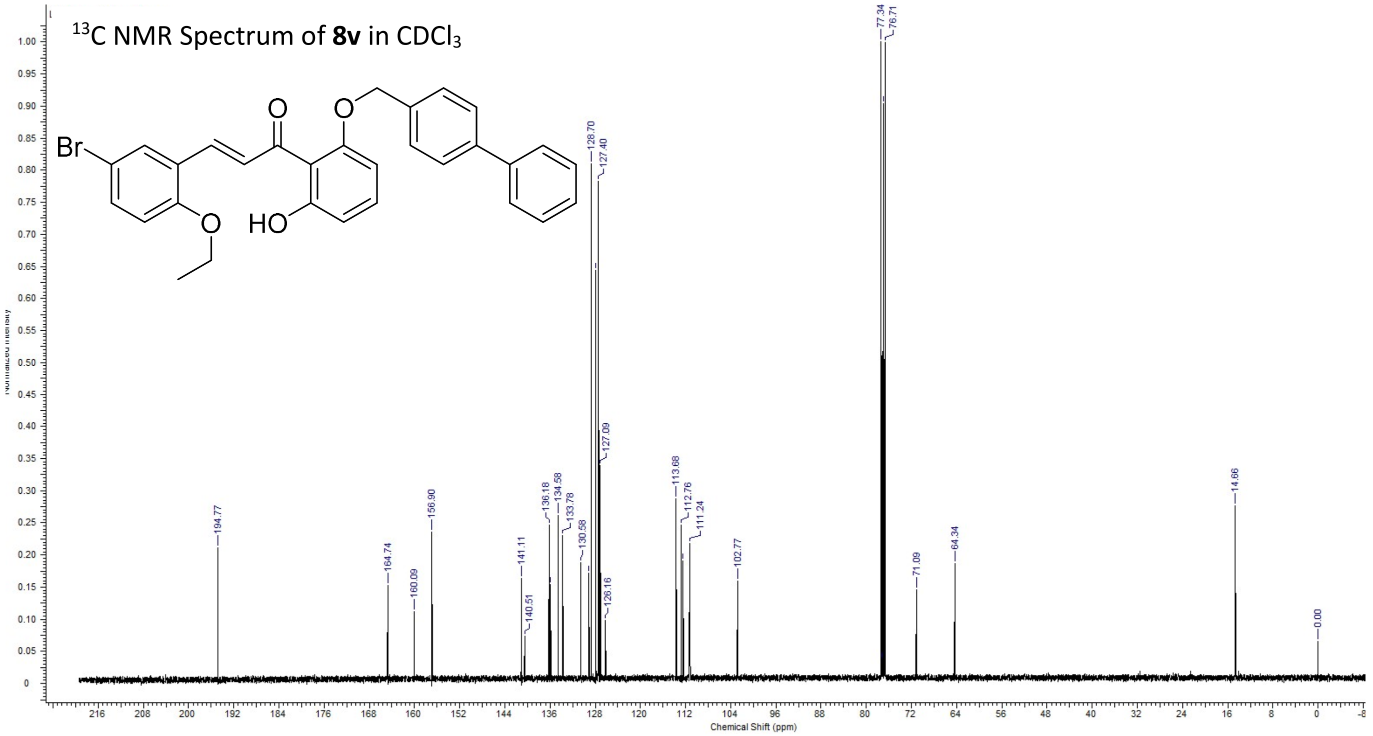

¹³C NMR Spectrum of 8v in CDCl₃
Br
O
HO
Chemical Shift (ppm)

Mass Spectrum of **8v**

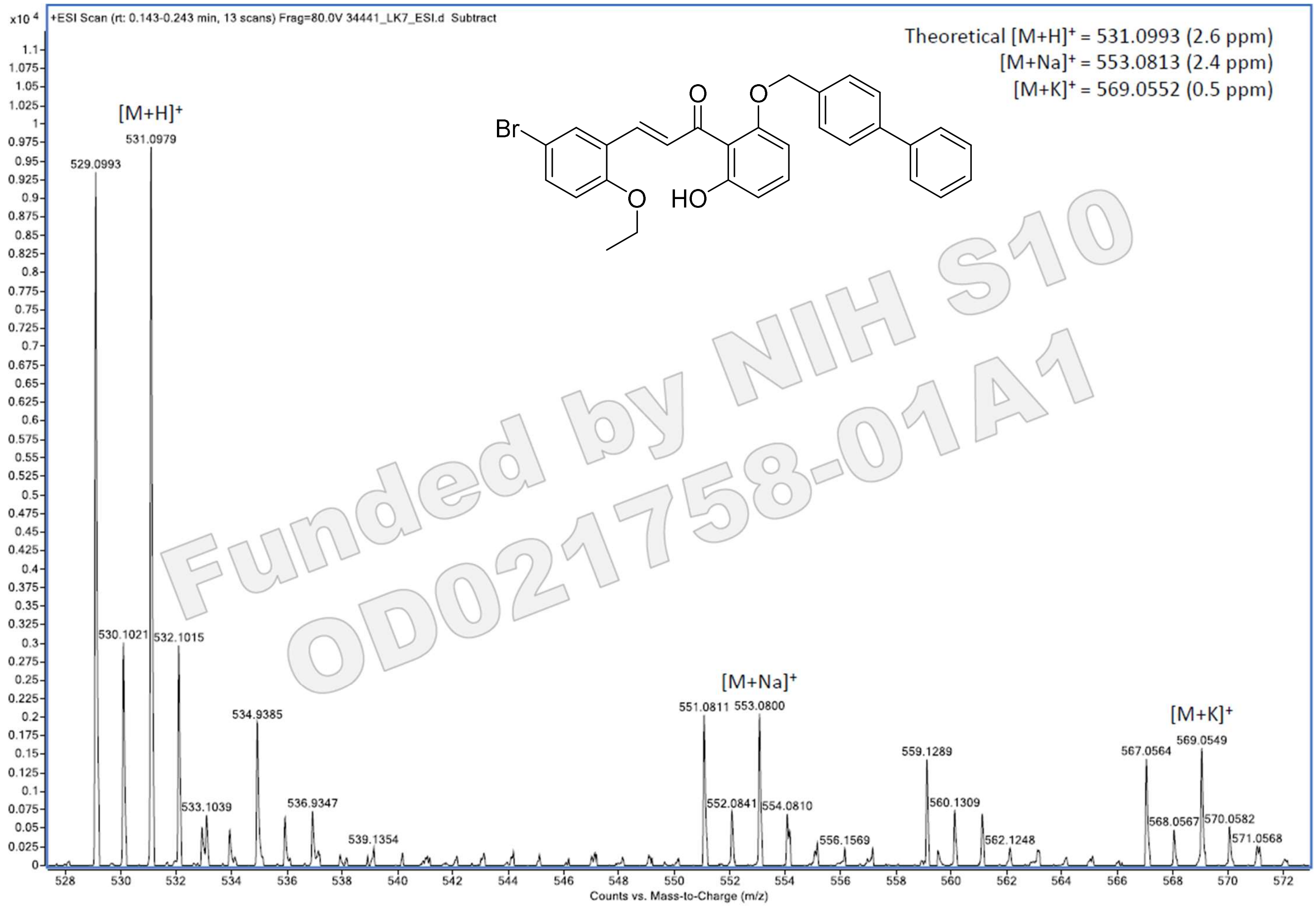

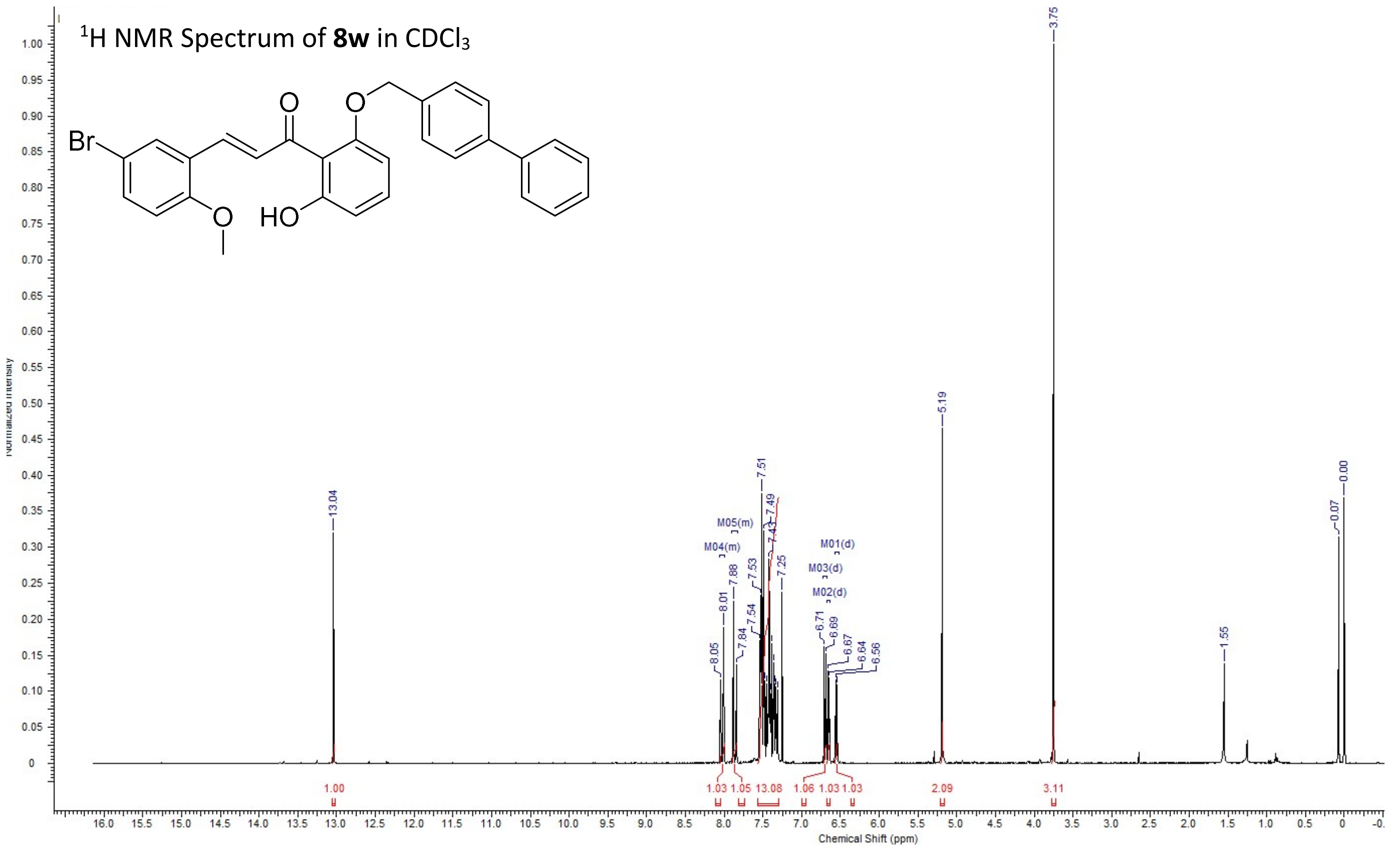

1H NMR Spectrum of 8w in CDCl3
Br
O
O
O
HO
13.04
8.05
8.01
7.88
7.84
7.54
7.53
7.51
7.49
7.43
7.25
6.71
6.69
6.67
6.64
6.56
5.19
3.75
1.55
0.07
0.00
M01(d)
M02(d)
M03(d)
M04(m)
M05(m)
1.00
1.03
1.05
13.08
1.06
1.03
1.03
2.09
3.11
Chemical Shift (ppm)
Normalized Intensity

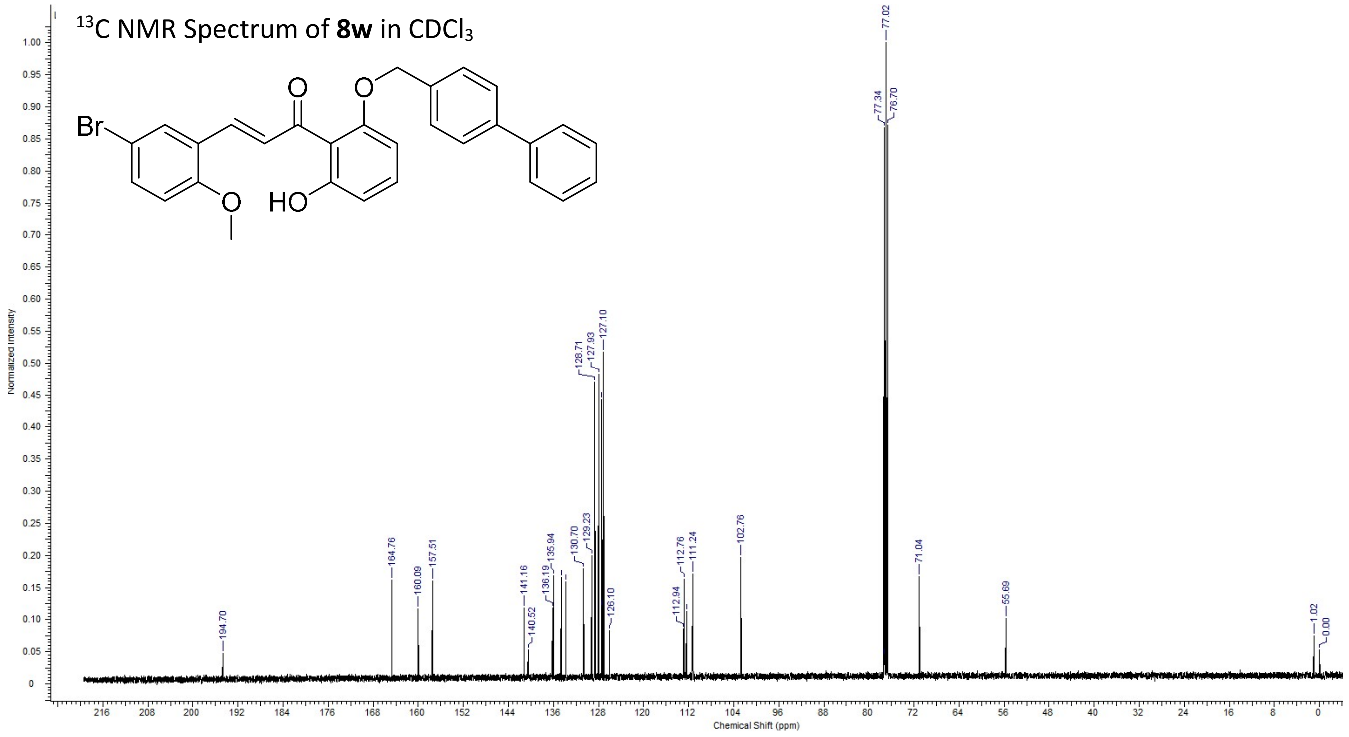

13C NMR Spectrum of 8w in CDCl3
Br
O
O
O
HO
194.70
164.76
160.09
157.51
141.16
140.52
136.19
135.94
130.70
129.23
128.71
127.93
127.10
126.10
112.94
112.76
111.24
102.76
77.34
77.02
76.70
71.04
55.69
1.02
0.00
Chemical Shift (ppm)
Normalized Intensity

Mass Spectrum of **8w**

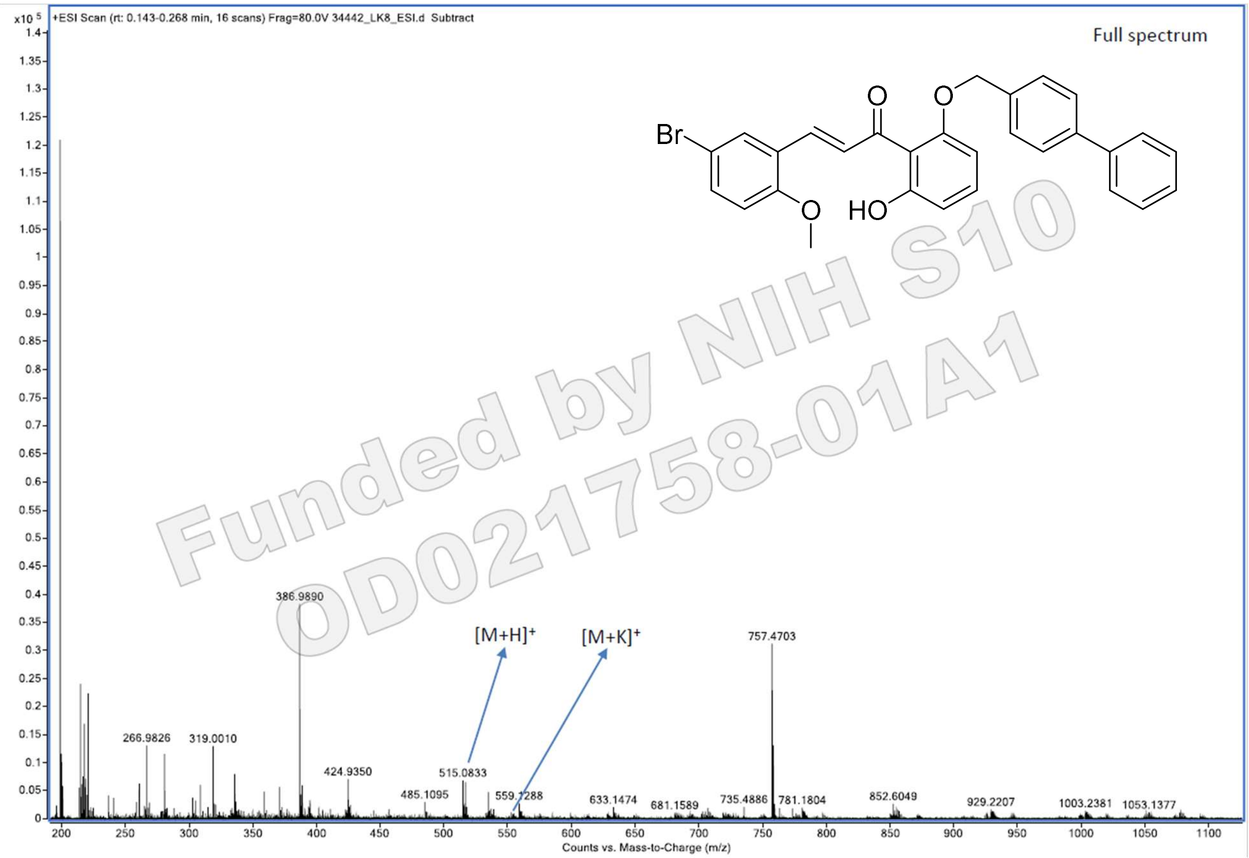

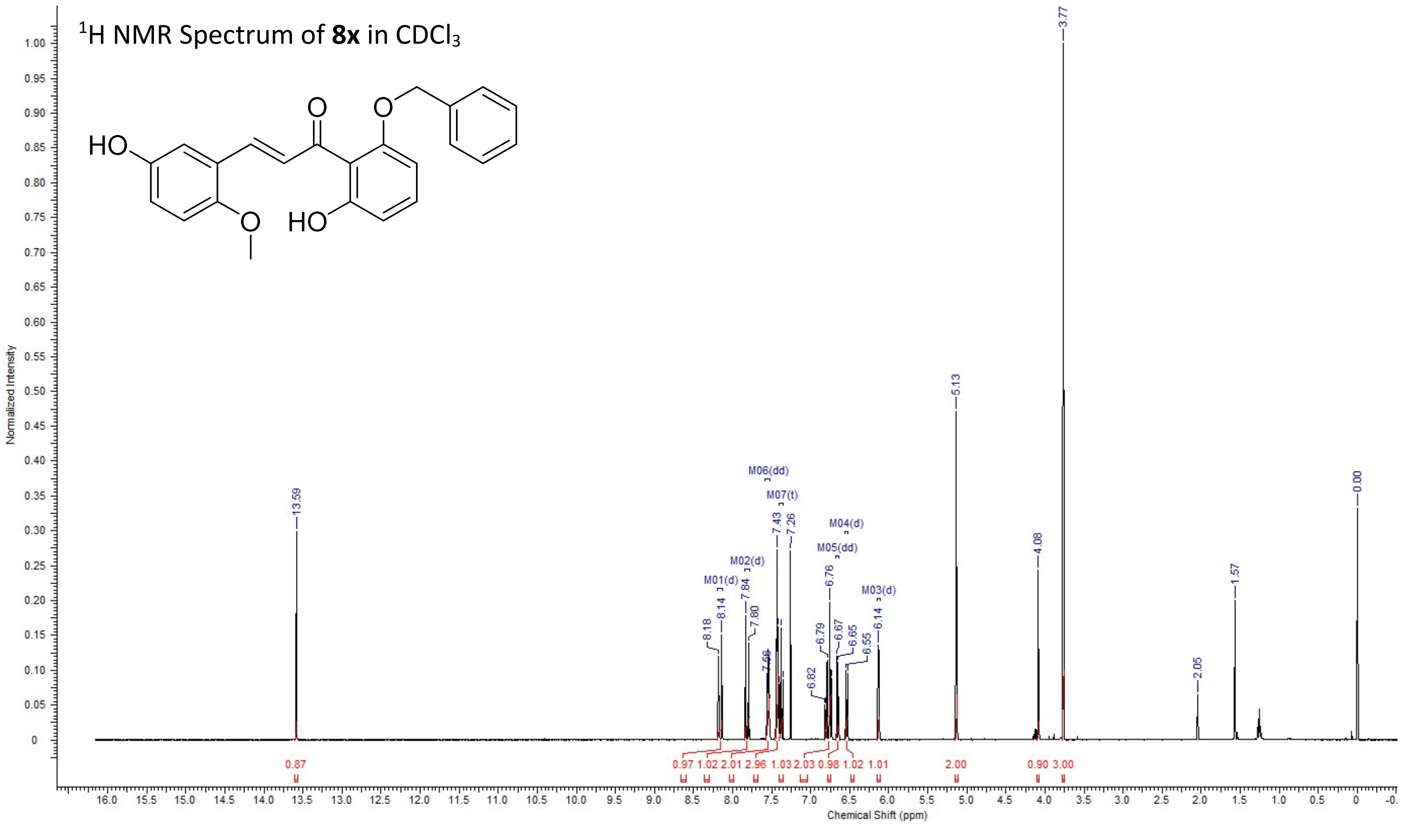

1H NMR Spectrum of 8x in CDCl3
HO
O
O
HO
O
Normalized Intensity
Chemical Shift (ppm)
13.59
M01(d)
M02(d)
M06(dd)
M07(t)
M04(d)
M05(dd)
M03(d)
8.18
8.14
7.84
7.80
7.56
7.43
7.26
6.82
6.79
6.76
6.67
6.65
6.55
6.14
5.13
4.08
3.77
2.05
1.57
0.00
0.87
0.97 1.02 2.01 2.96 1.03 2.03 0.98 1.02 1.01
2.00
0.90 3.00

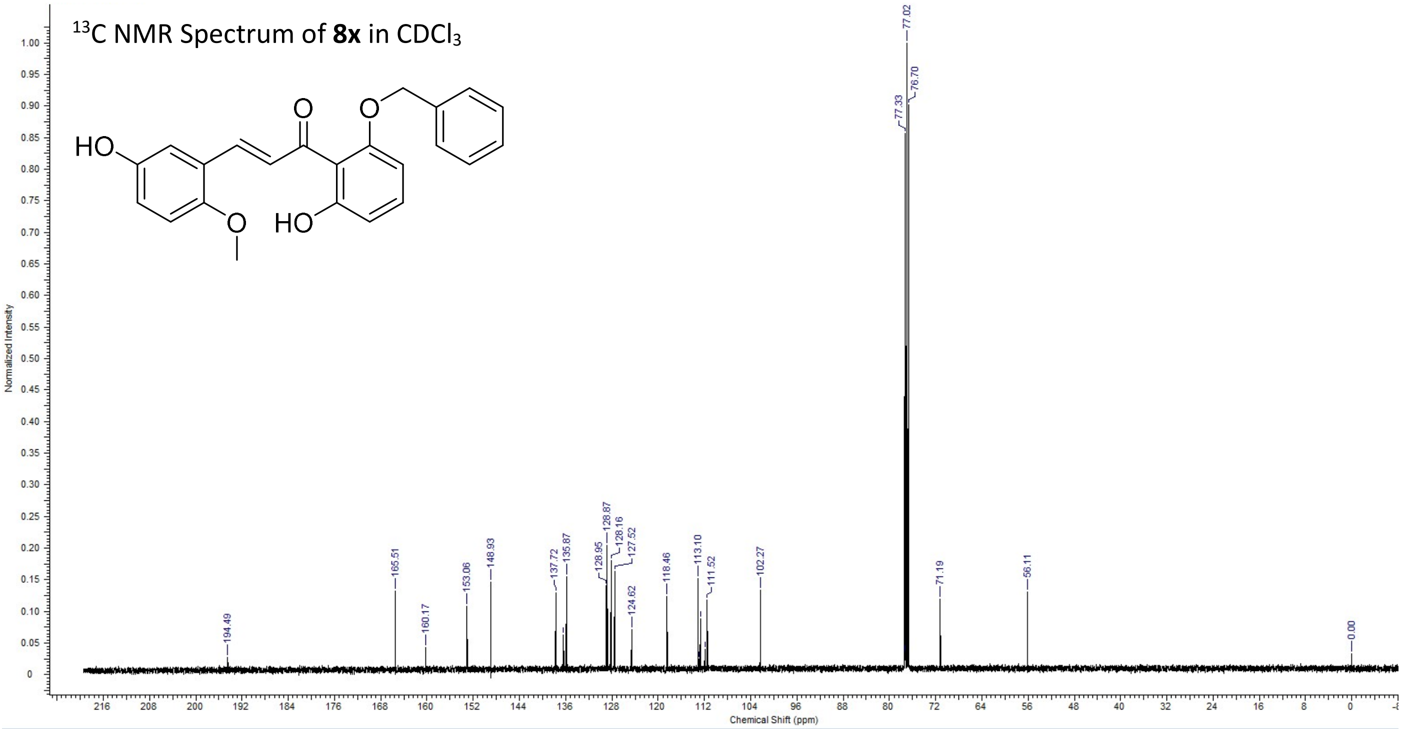

13C NMR Spectrum of 8x in CDCl3
HO
O
O
HO
O
Normalized Intensity
Chemical Shift (ppm)
194.49
165.51
160.17
153.06
148.93
137.72
135.87
128.95
128.87
128.16
127.52
124.62
118.46
113.10
111.52
102.27
77.33
77.02
76.70
71.19
56.11
0.00

Mass Spectrum of **8x**

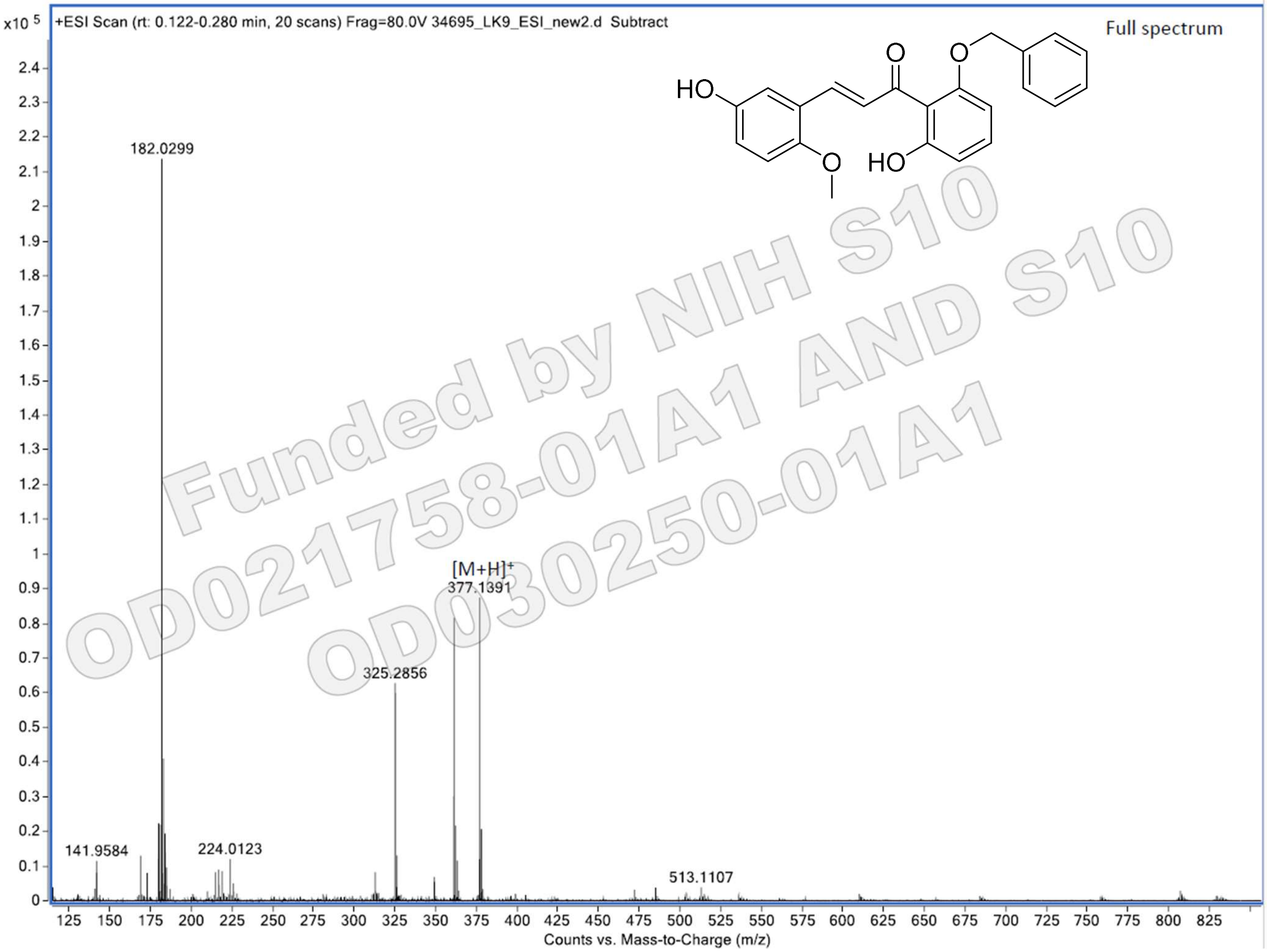

HPLC of **8x**

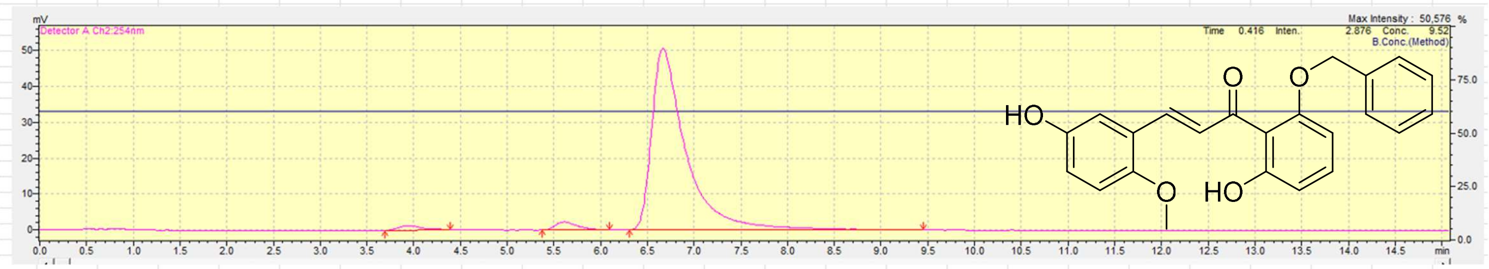

LK9: 0.75 mL/min @ 60% acetonitrile

254nm (10 uL)

Ret. Time	Area	% Purity	% Purity (-MeOH)
3.939	23514	95.48	97.17
5.609	37451		
6.665	1286829		

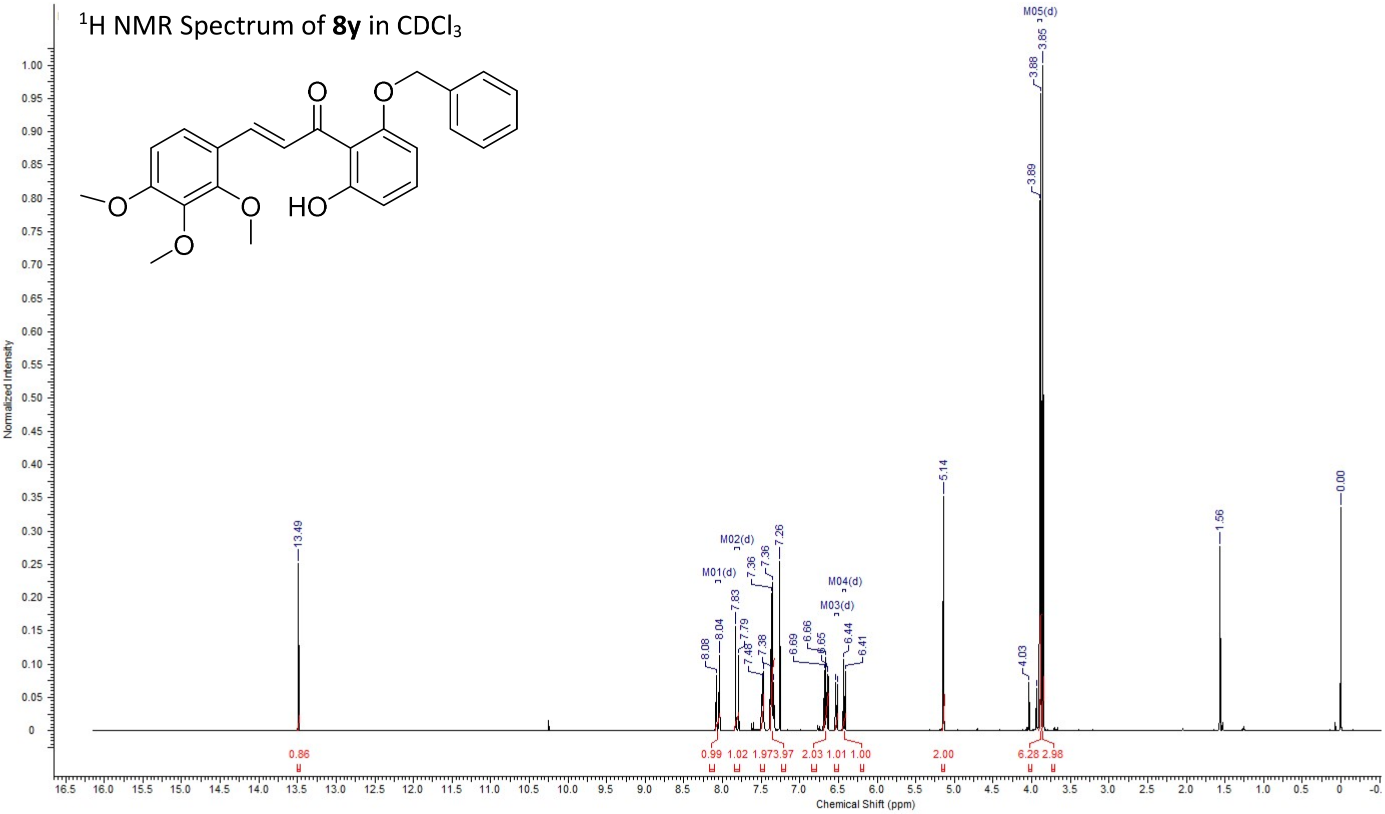

^{1}H NMR Spectrum of **8y** in CDCl$_3$
Normalized Intensity
Chemical Shift (ppm)

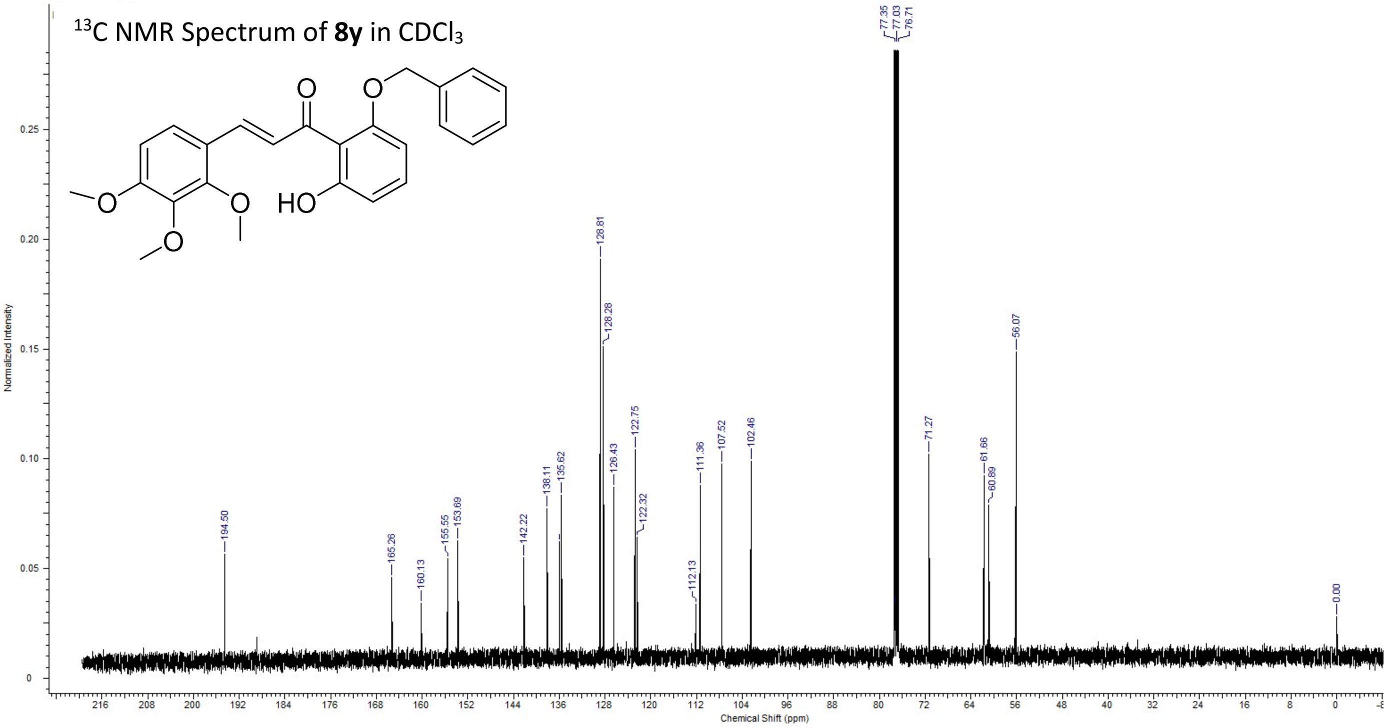

^{13}C NMR Spectrum of **8y** in CDCl$_3$
Normalized Intensity
Chemical Shift (ppm)

Mass Spectrum of **8y**

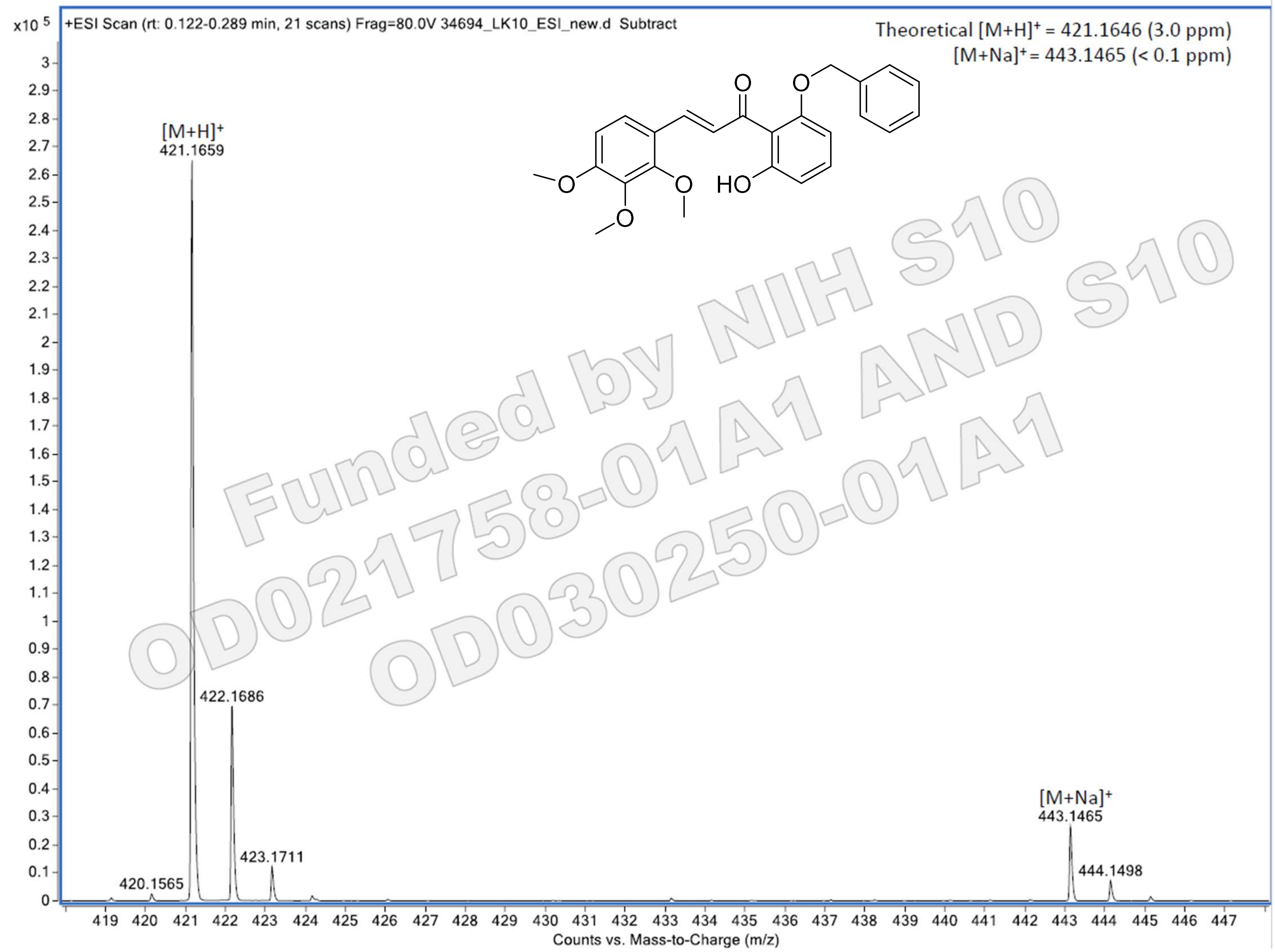

Funded by NIH S10 OD021758-01A1 AND S10 OD030250-01A1

HPLC of **8y**

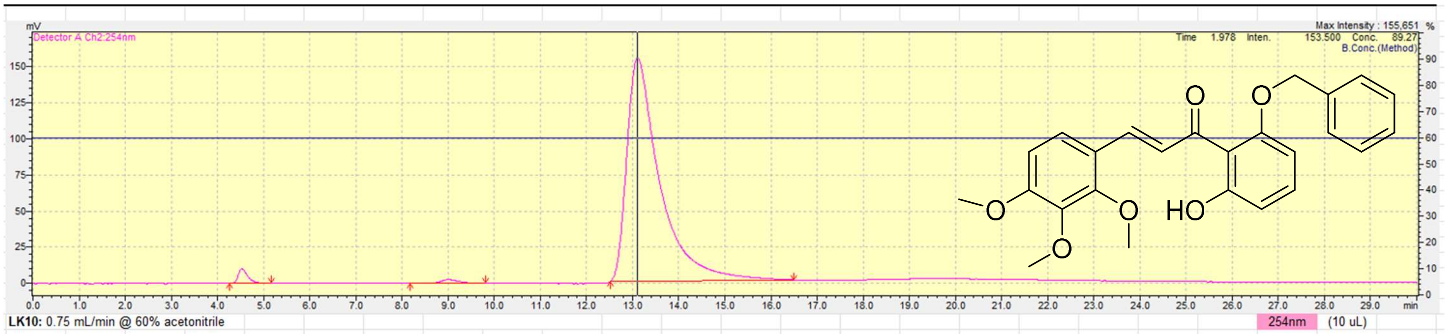

Ret. Time	Area	% Purity	% Purity (-MeOH)
4.527	141554	97.23	99.02
9.017	76003		
13.106	7644749		

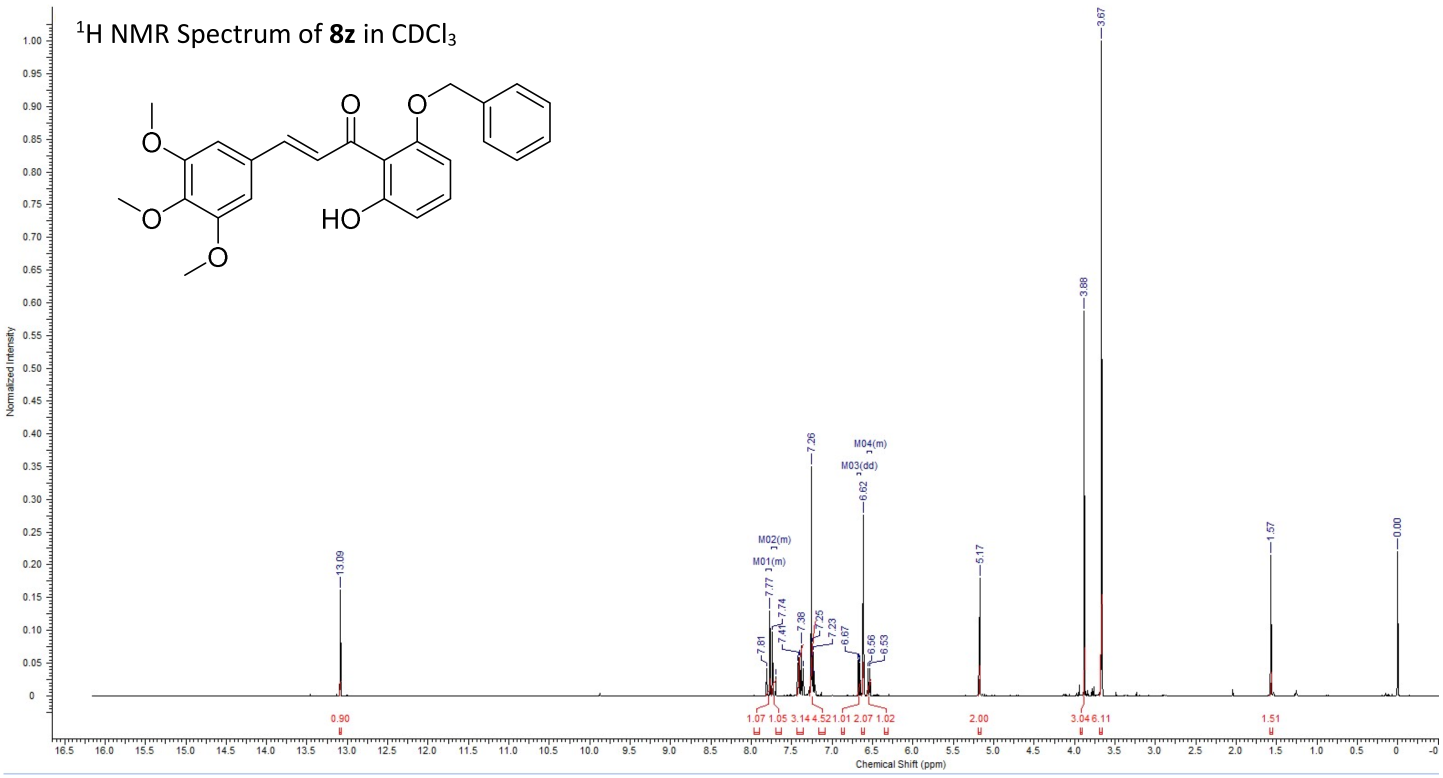
^{1}H NMR Spectrum of **8z** in CDCl$_3$
HO
O
Normalized Intensity
Chemical Shift (ppm)
13.09
7.81
7.77
7.74
7.41
7.38
7.26
7.25
7.23
6.67
6.62
6.56
6.53
5.17
3.88
3.67
1.57
0.00
M01(m)
M02(m)
M03(dd)
M04(m)
0.90
1.07
1.05
3.14
4.52
1.01
2.07
1.02
2.00
3.04
6.11
1.51

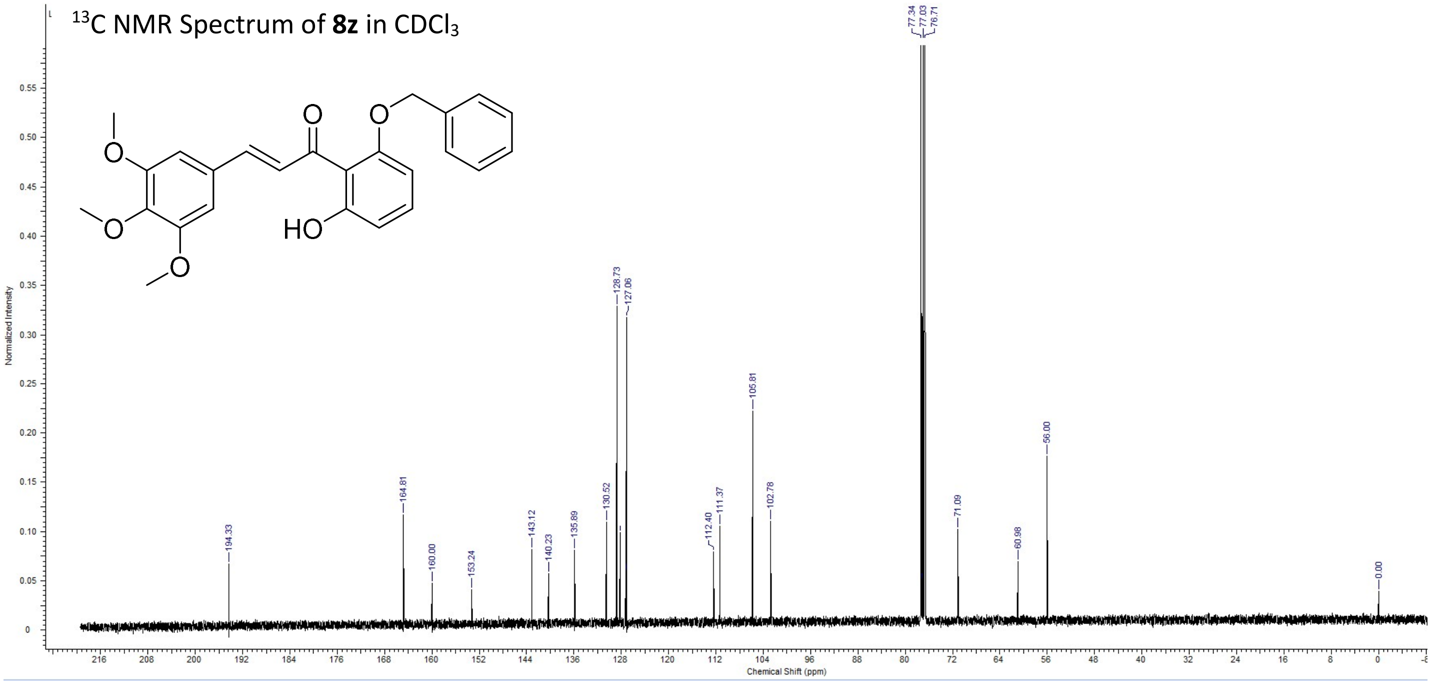
^{13}C NMR Spectrum of **8z** in CDCl$_3$
HO
O
Normalized Intensity
Chemical Shift (ppm)
194.33
164.81
160.00
153.24
143.12
140.23
135.89
130.52
128.73
127.06
112.40
111.37
105.81
102.78
77.34
77.03
76.71
71.09
60.98
56.00
0.00

Mass Spectrum of **8z**

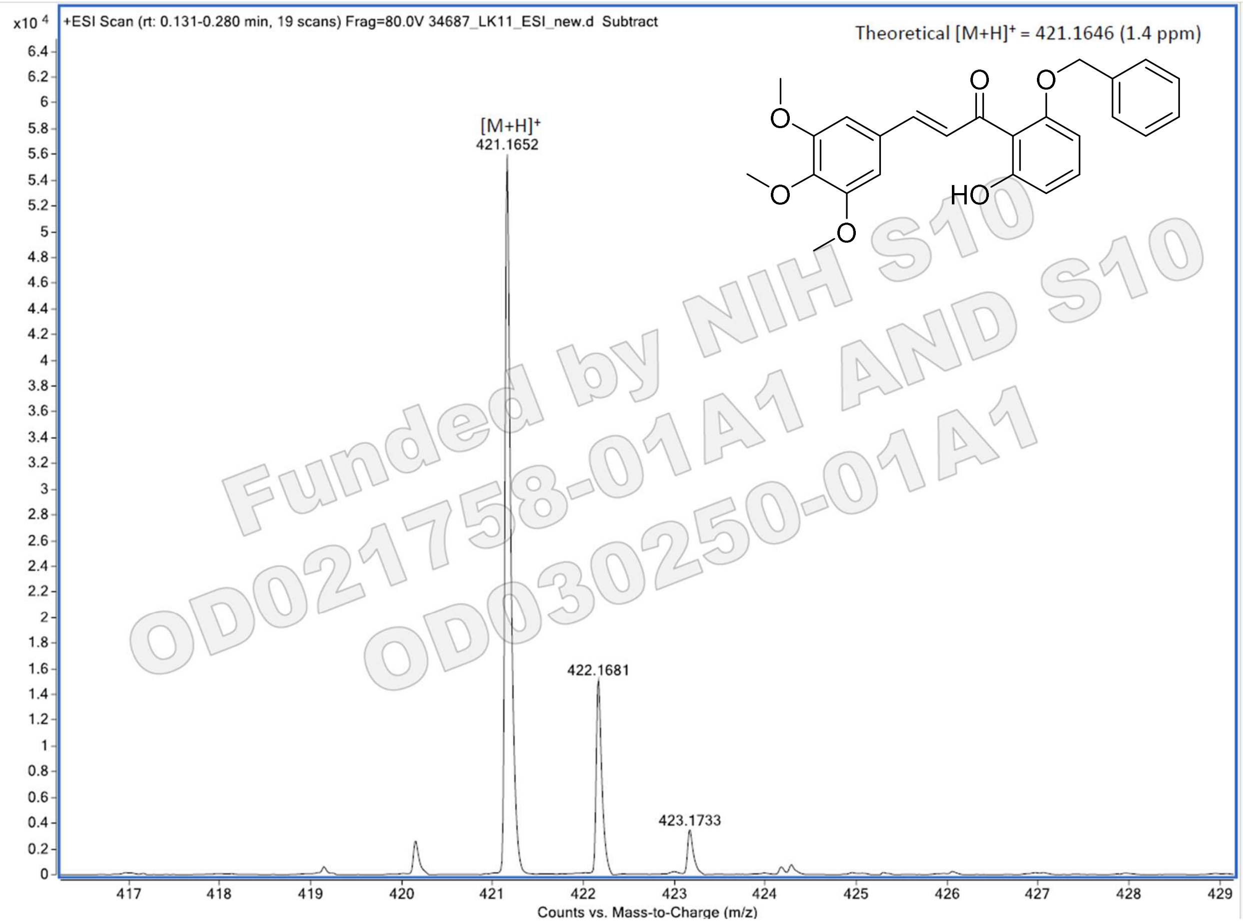

HPLC of **8z**

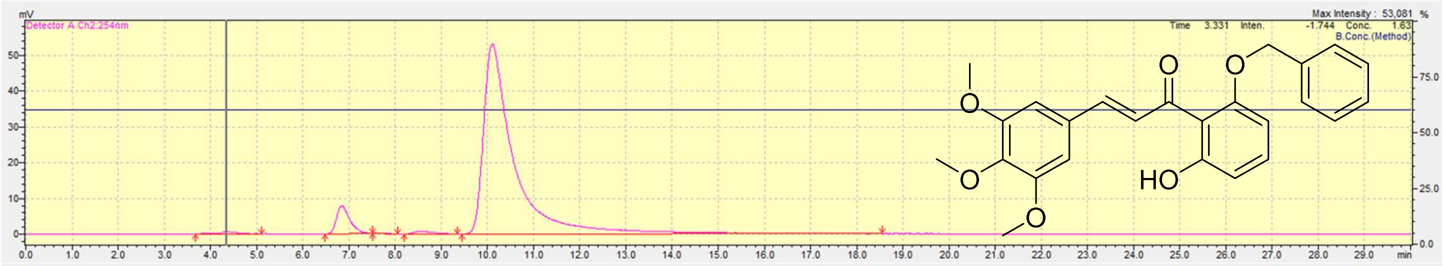

LK11: 0.75 mL/min @ 60% acetonitrile

254nm (10 uL)

Ret. Time	Area	% Purity	% Purity (-MeOH)
4.34	21849	92.36	93.13
6.844	157599		
7.568	1788		
8.551	21541		
10.102	2451936		

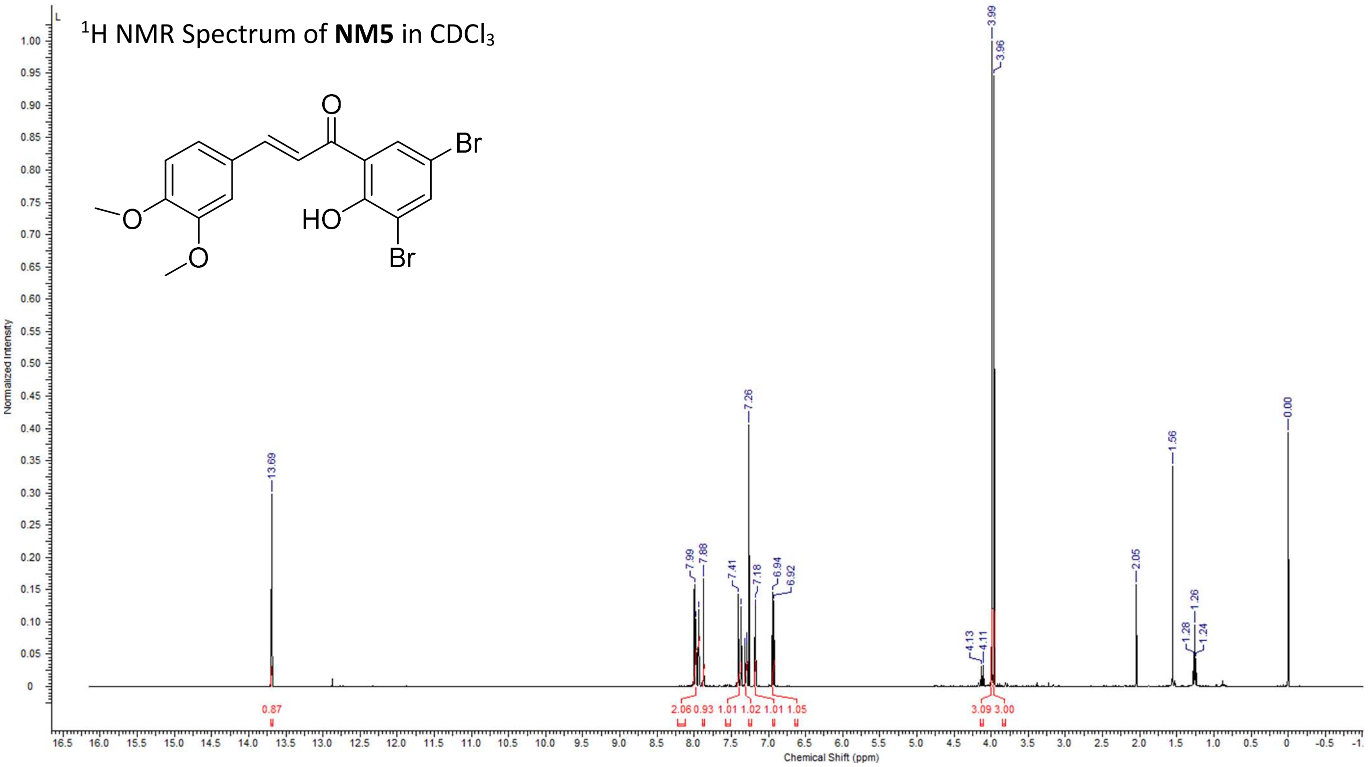

^{1}H NMR Spectrum of **NM5** in $CDCl_3$
O
Br
HO
Br
13.69
7.99
7.88
7.41
7.26
7.18
6.94
6.92
4.13
4.11
3.99
3.96
2.05
1.56
1.28
1.26
1.24
0.00
0.87
2.06
0.93
1.01
1.02
1.01
1.05
3.09
3.00
Normalized Intensity
Chemical Shift (ppm)

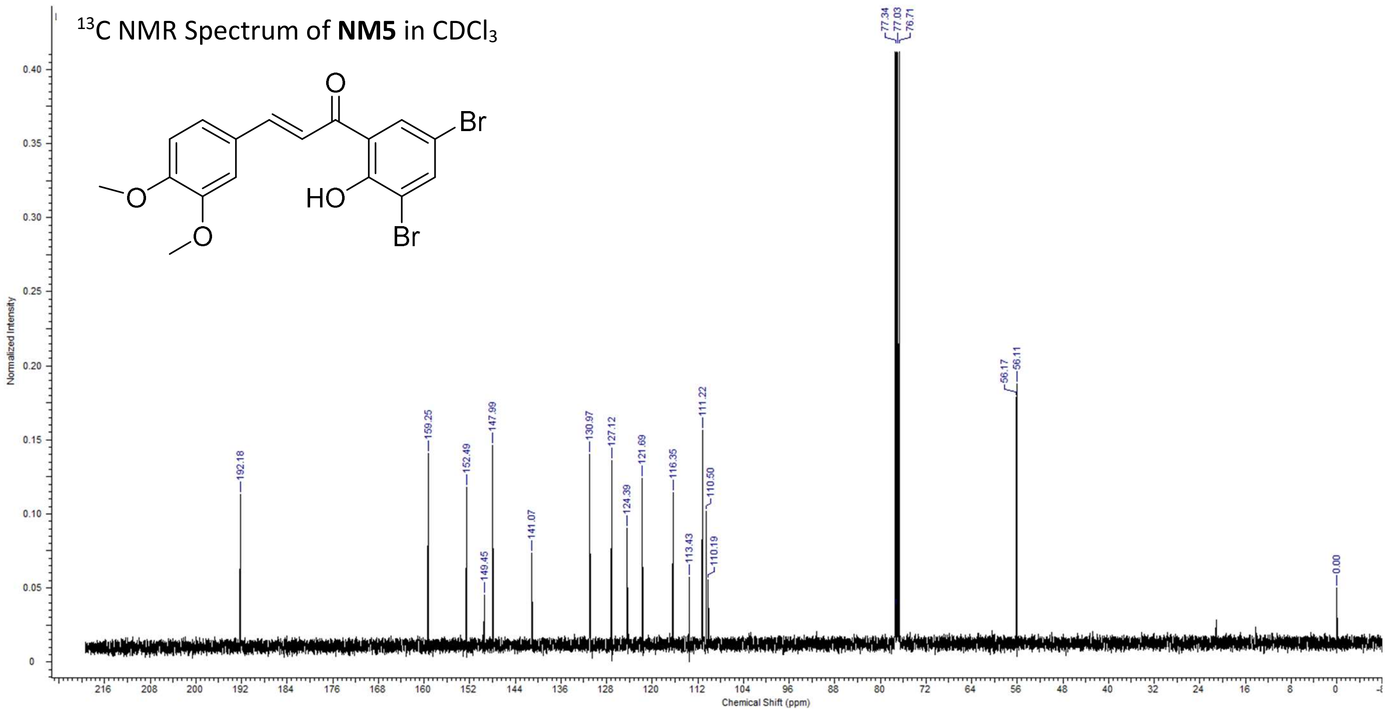

^{13}C NMR Spectrum of **NM5** in $CDCl_3$
O
Br
HO
Br
192.18
159.25
152.49
149.45
147.99
141.07
130.97
127.12
124.39
121.69
116.35
113.43
111.22
110.50
110.19
77.34
77.03
76.71
56.17
56.11
0.00
Normalized Intensity
Chemical Shift (ppm)

Mass Spectrum of **NM5**

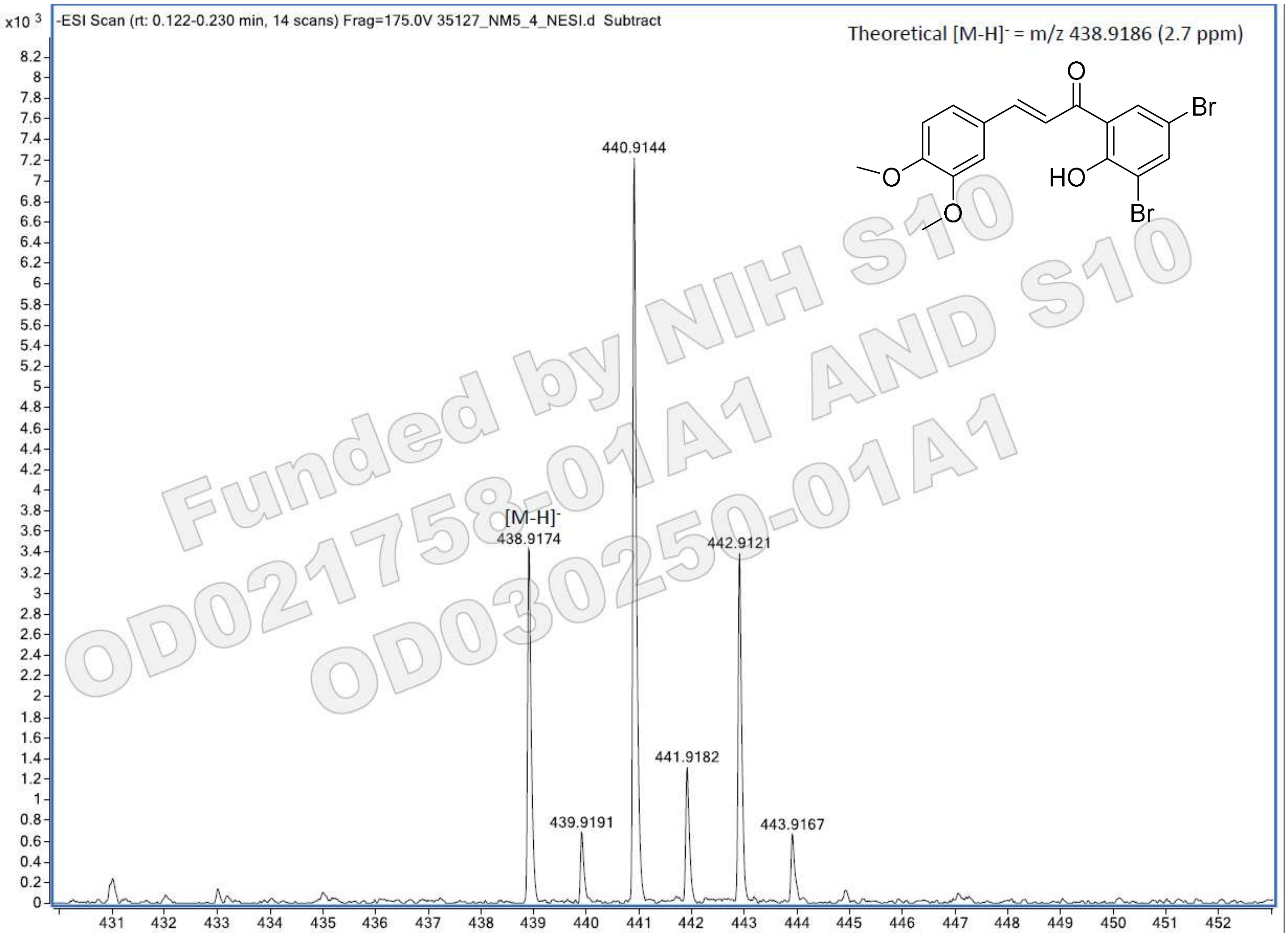

Table S1. Elemental Analyses for **7d, 8q-8w** and **NM5.**[a]

Elemental Analysis		Theory		Found	
**Compound**	**Formula**	**C**	**H**	**C**	**H**
**7d**	$C_{21}H_{18}O_3$	79.23	5.70	79.10	5.72
**8q**	$C_{18}H_{17}BrO_4$	57.31	4.54	57.22	4.53
**8r**	$C_{22}H_{25}BrO_4.H_2O$	60.35	5.87	60.34	5.90
**8s**	$C_{21}H_{23}BrO_4$	60.15	5.53	60.17	5.56
**8t**	$C_{25}H_{23}BrO_4$	64.25	4.96	64.05	5.29
**8u**	$C_{24}H_{21}BrO_4$	63.59	4.67	63.86	4.81
**8v**	$C_{30}H_{25}BrO_4.H_2O$	67.71	4.79	67.42	4.72
**8w**	$C_{29}H_{23}BrO_4.H_2O$	66.65	4.59	66.27	4.51
**NM5**	$C_{17}H_{14}Br_2O_4$	46.19	3.19	46.46	3.13

[a] the purity of compounds **8x-8z** were verified by HPLC (see above)

Original Western Blot Data

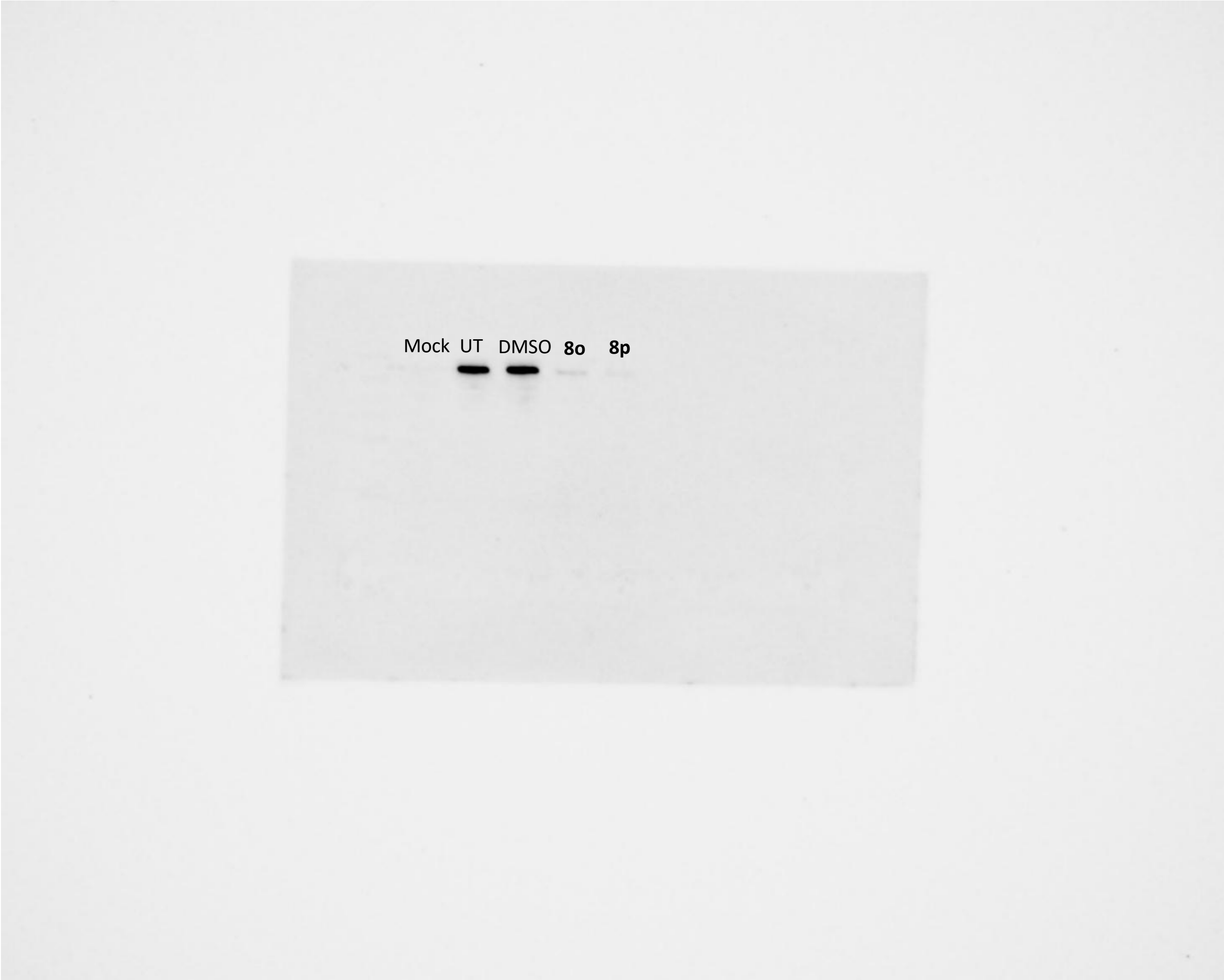

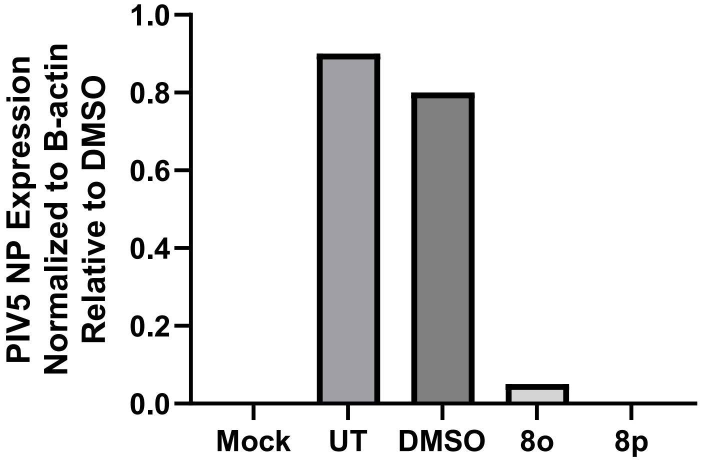

PIV5 NP Western Blot Uncropped Image and Intensity plot

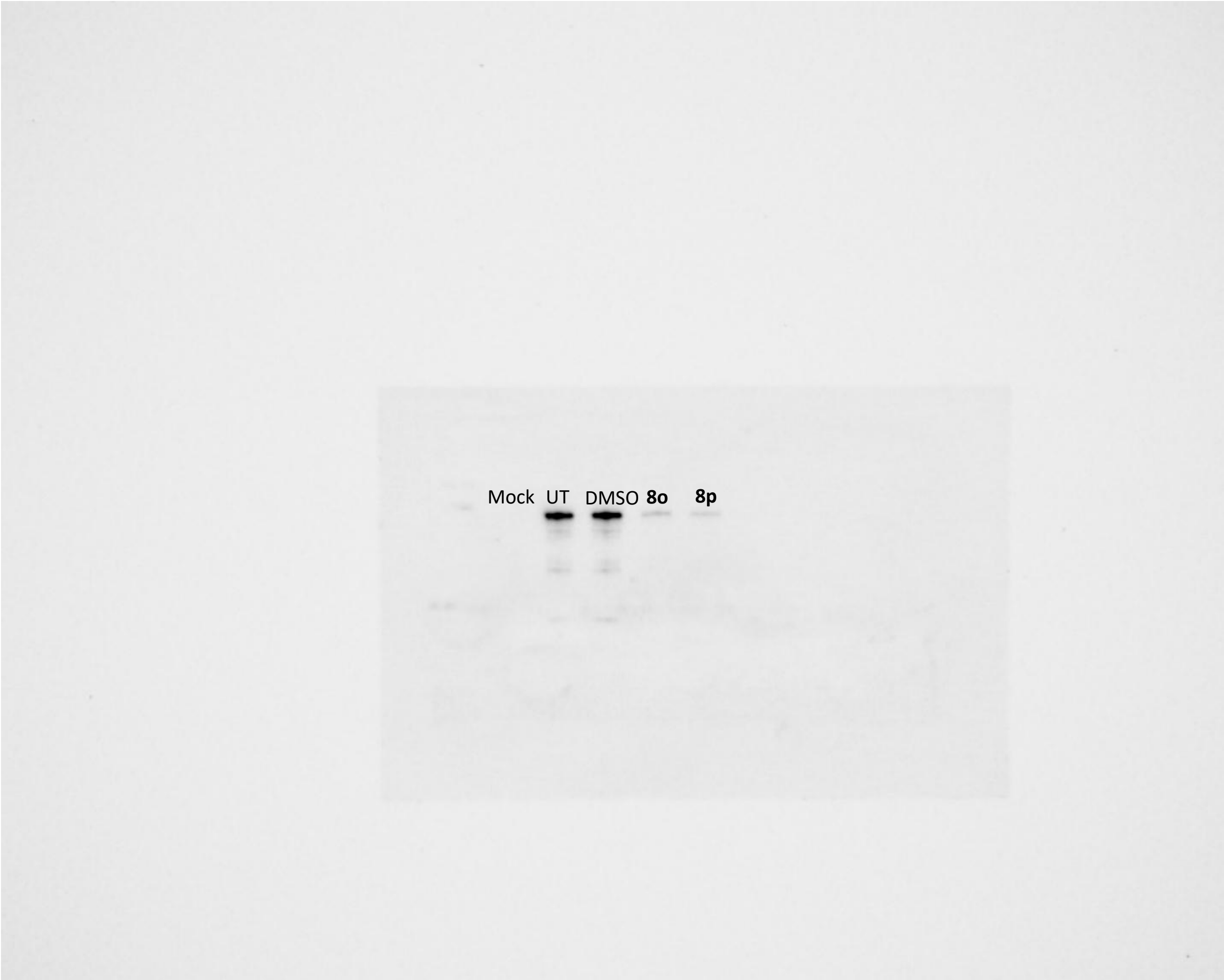

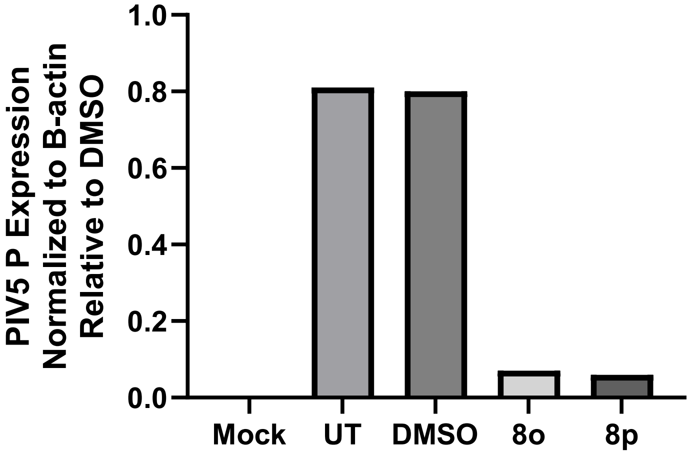

PIV5 P Western Blot Uncropped Image and Intensity Plot

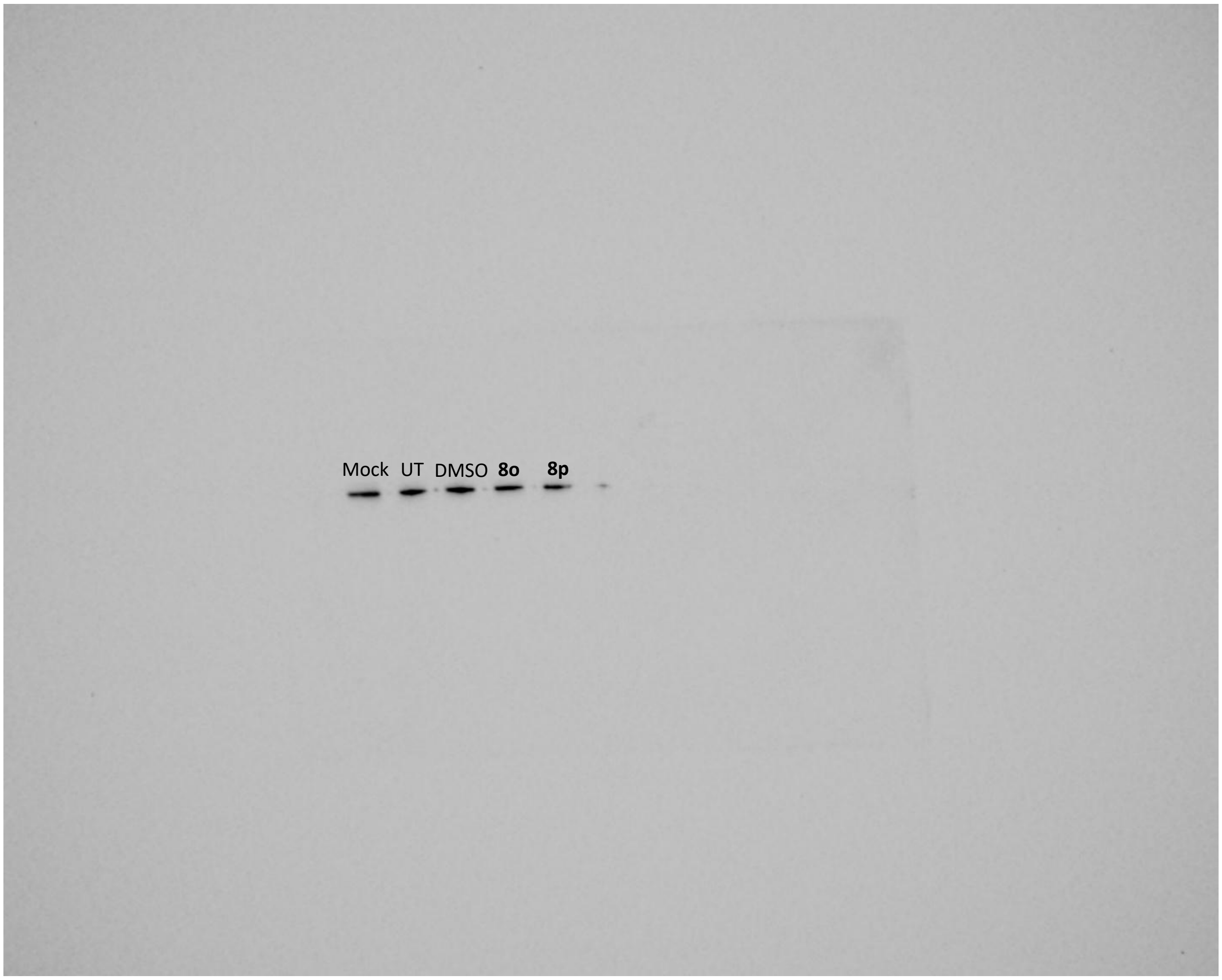

PIV5 B-Actin Western Blot Uncropped Image

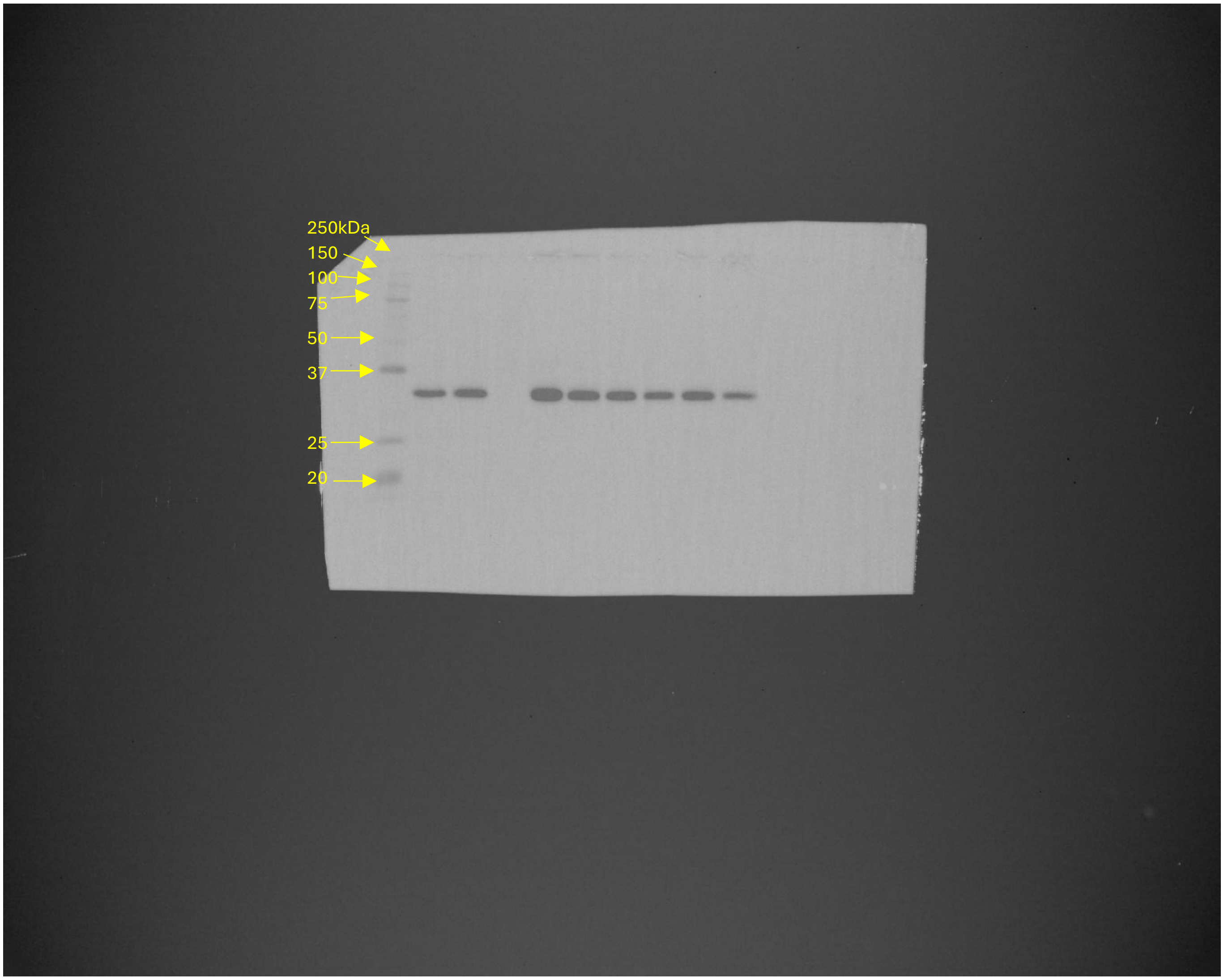

PANC-1 Western Blot Merged Uncropped Image of Membrane 1 for prps6

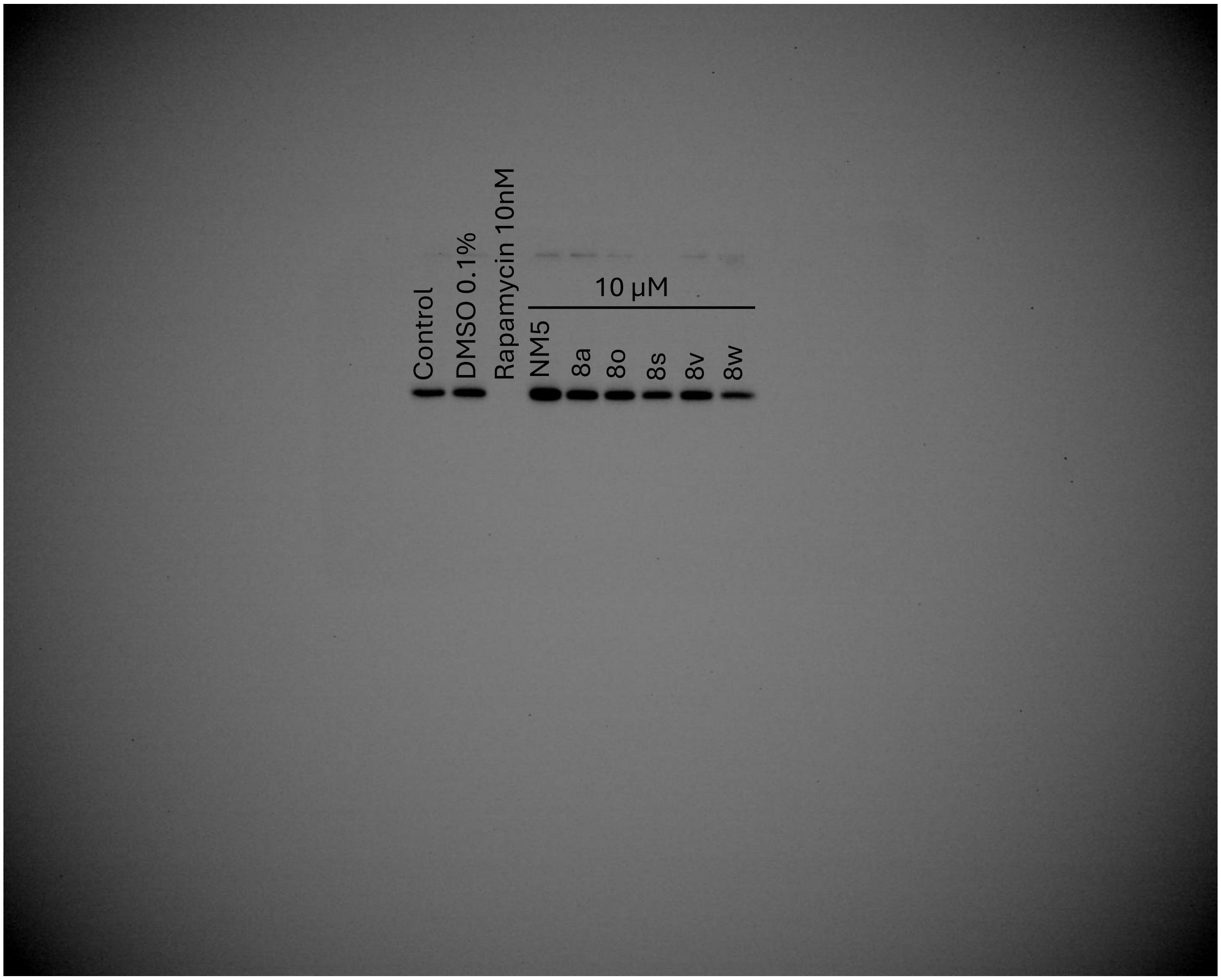

PANC-1 Western Blot Uncropped Image of Membrane 1 for prps6

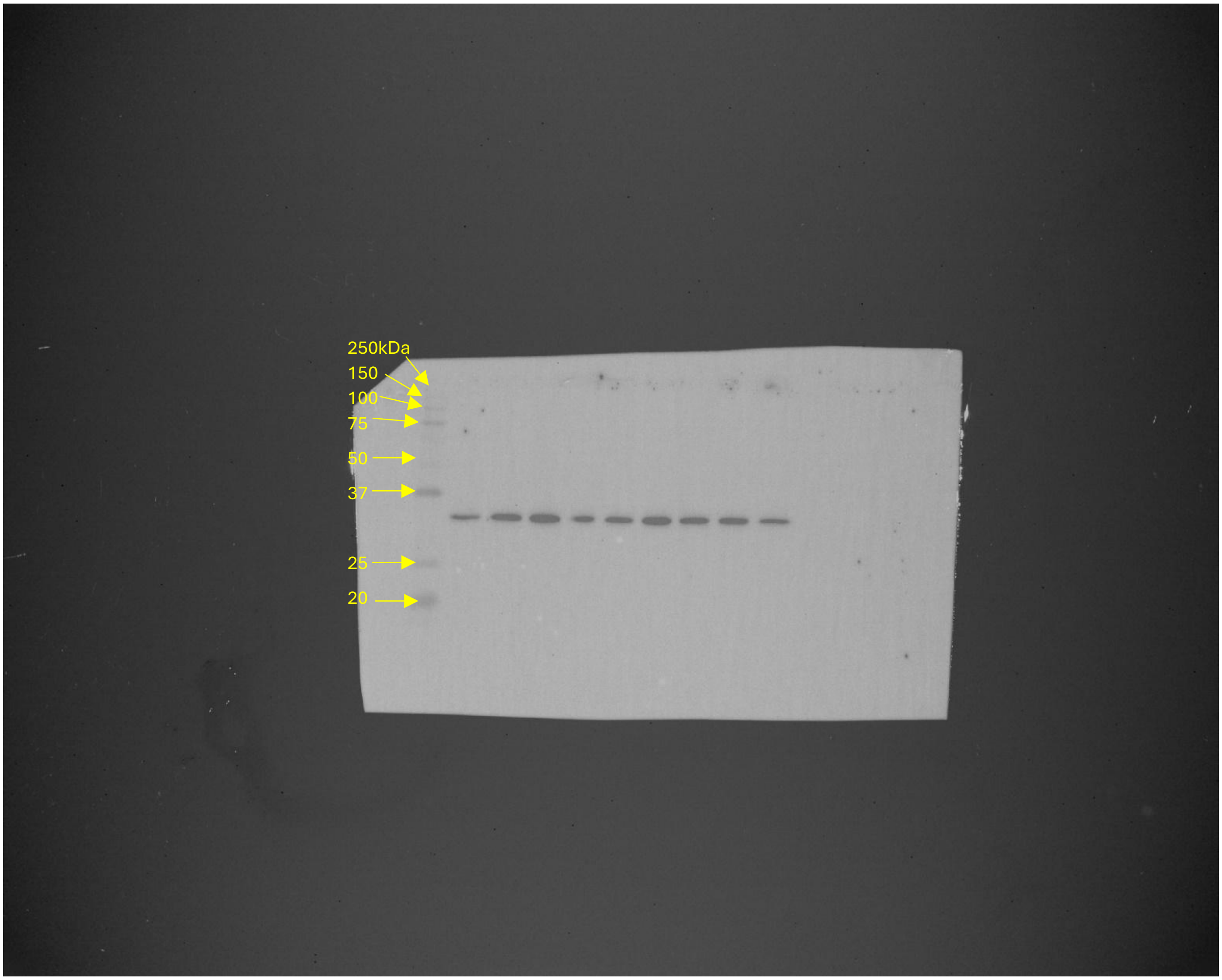

PANC-1 Western Blot Merged Uncropped Image of Membrane 1 for rps6

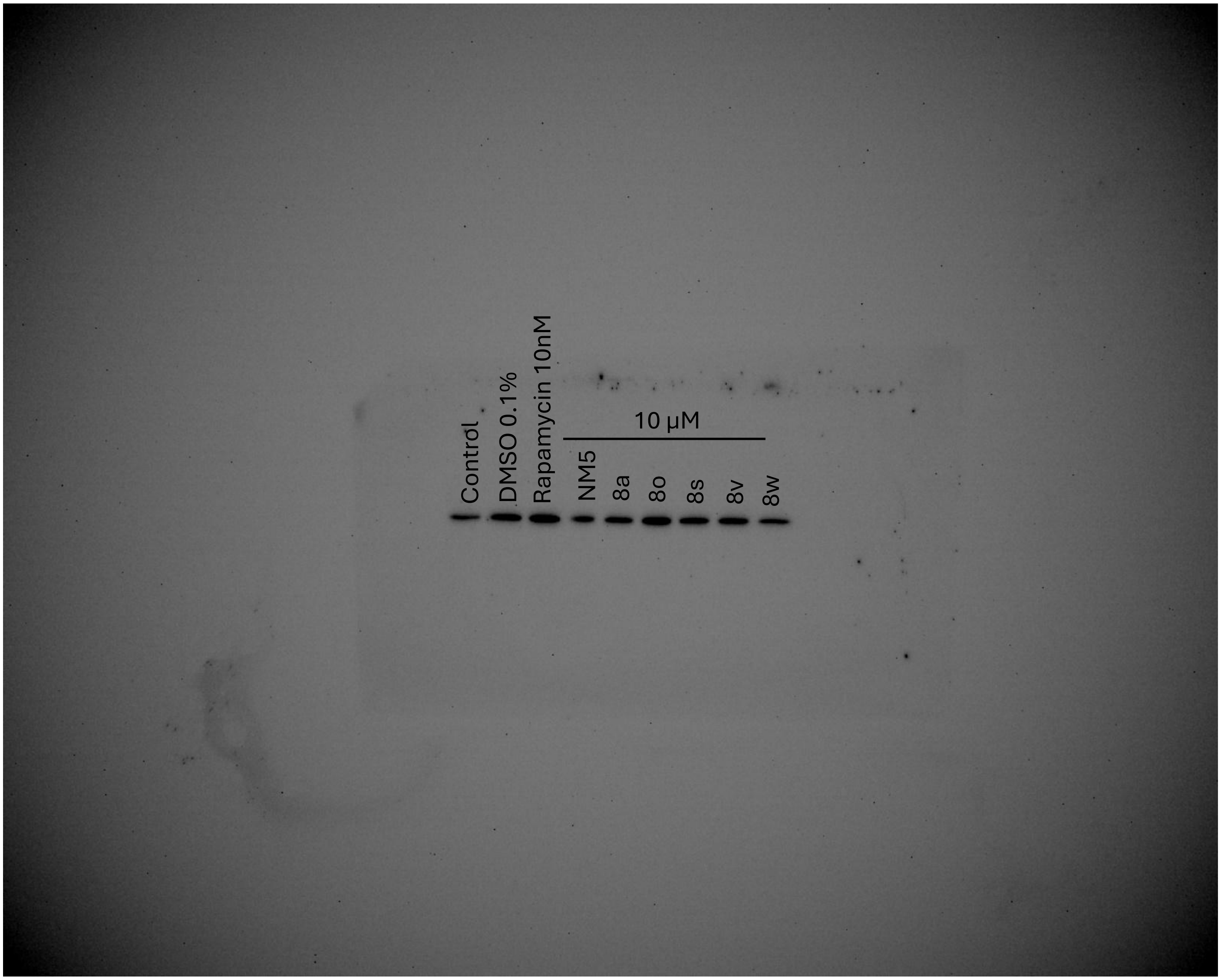

PANC-1 Western Blot Uncropped Image of Membrane 1 for rps6

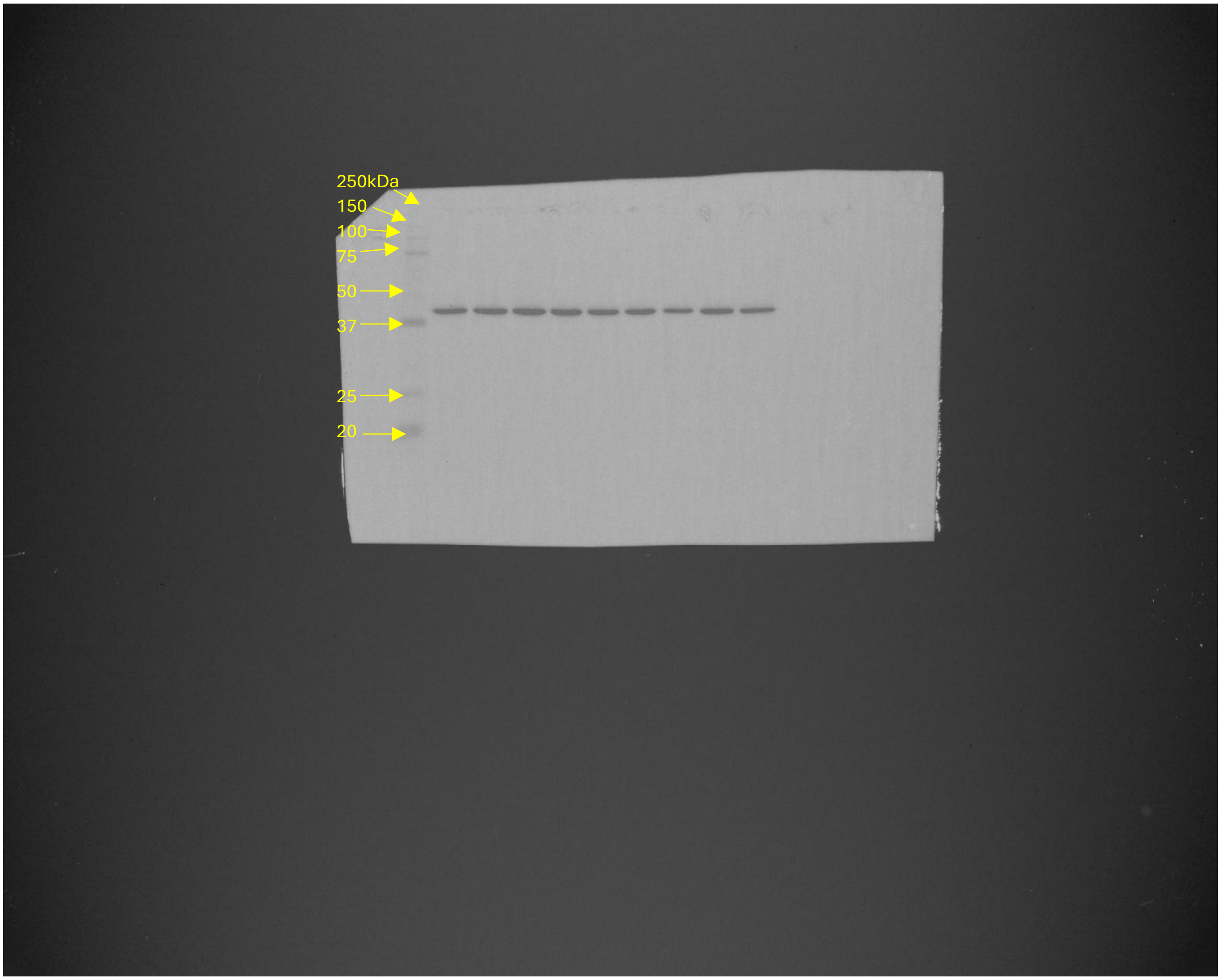

PANC-1 Western Blot Merged Uncropped Image of Membrane 1 for β-actin

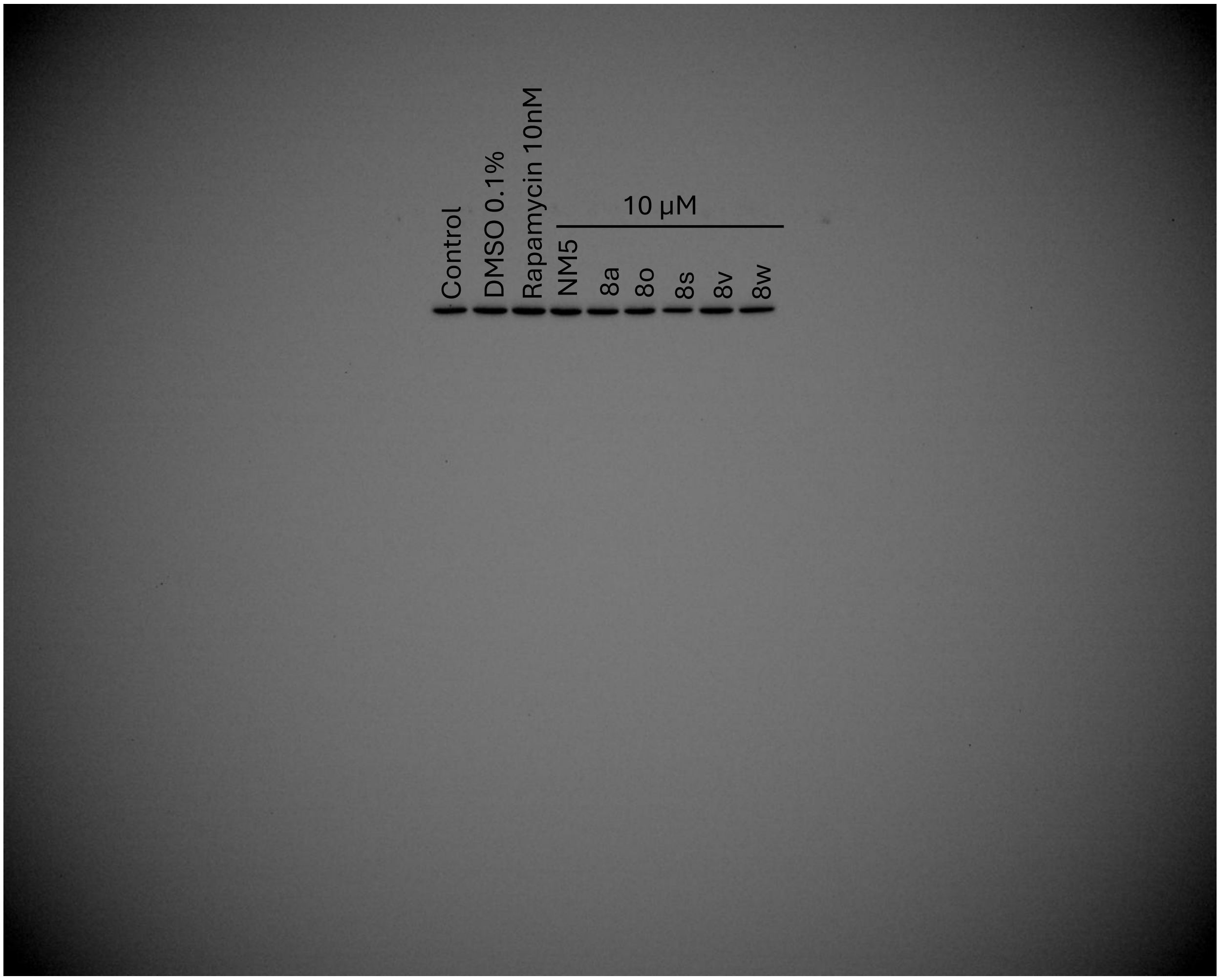

PANC-1 Western Blot Uncropped Image of Membrane 1 for β-actin

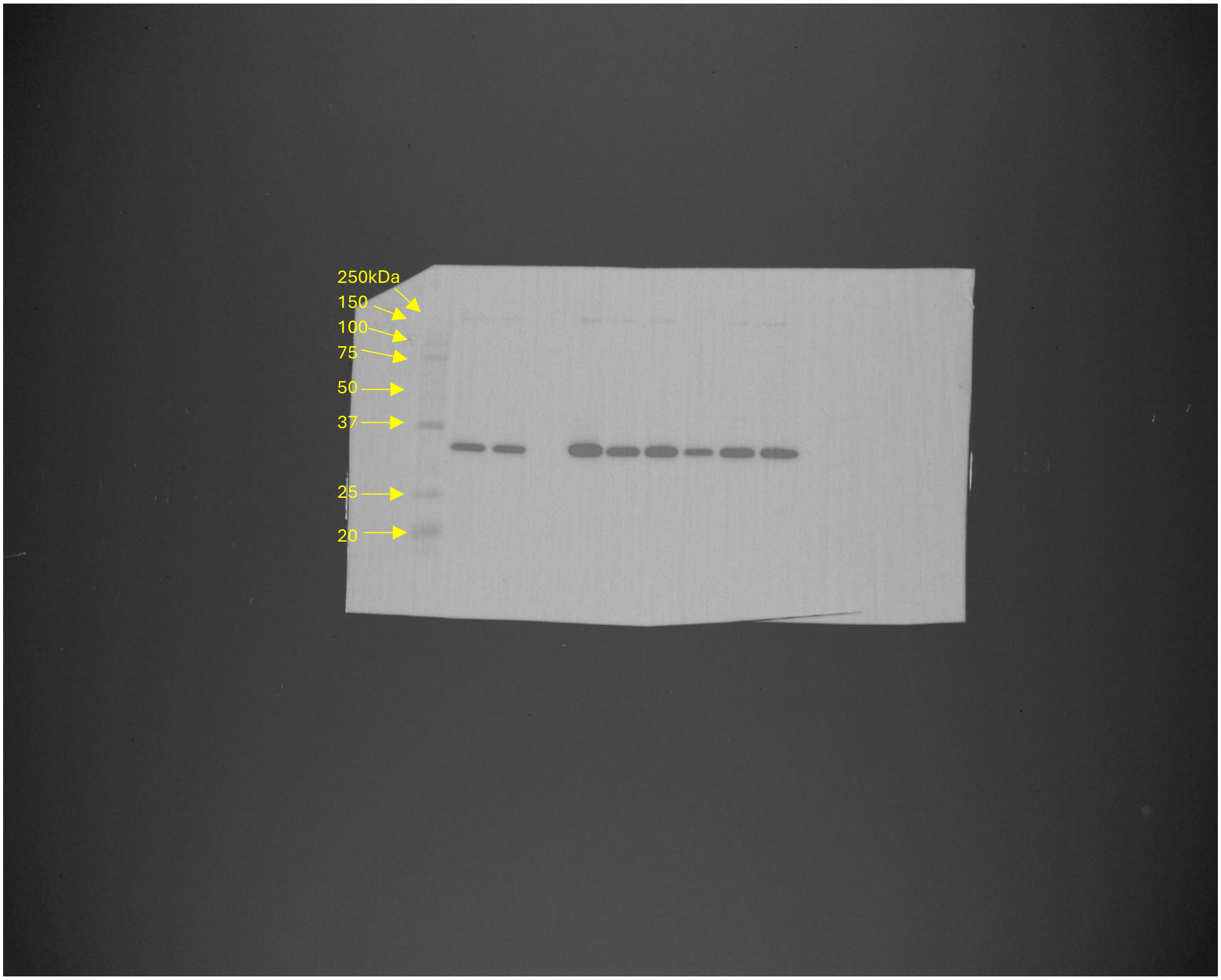

PANC-1 Western Blot Merged Uncropped Image of Membrane 2 for prps6

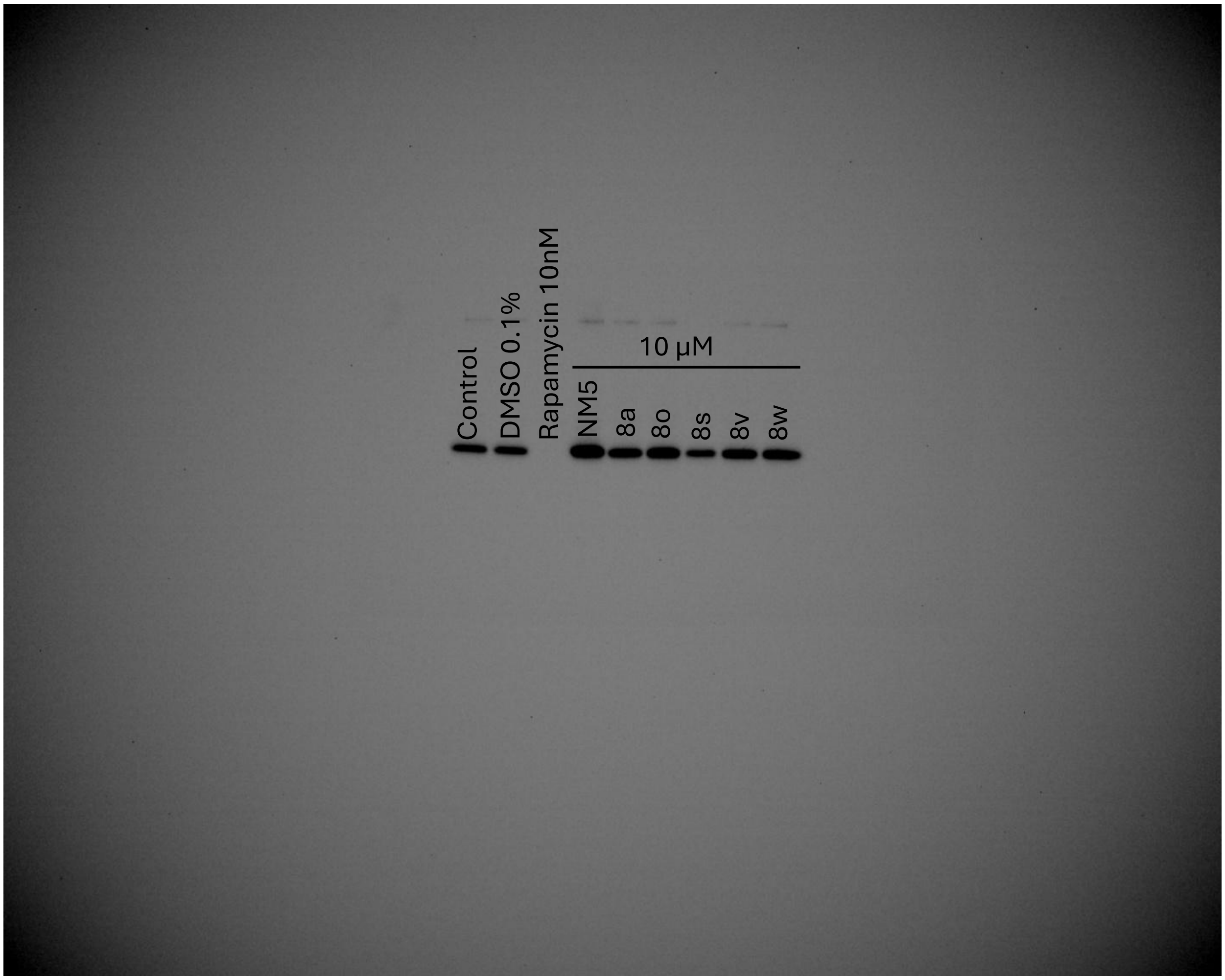

PANC-1 Western Blot Uncropped Image of Membrane 2 for prps6

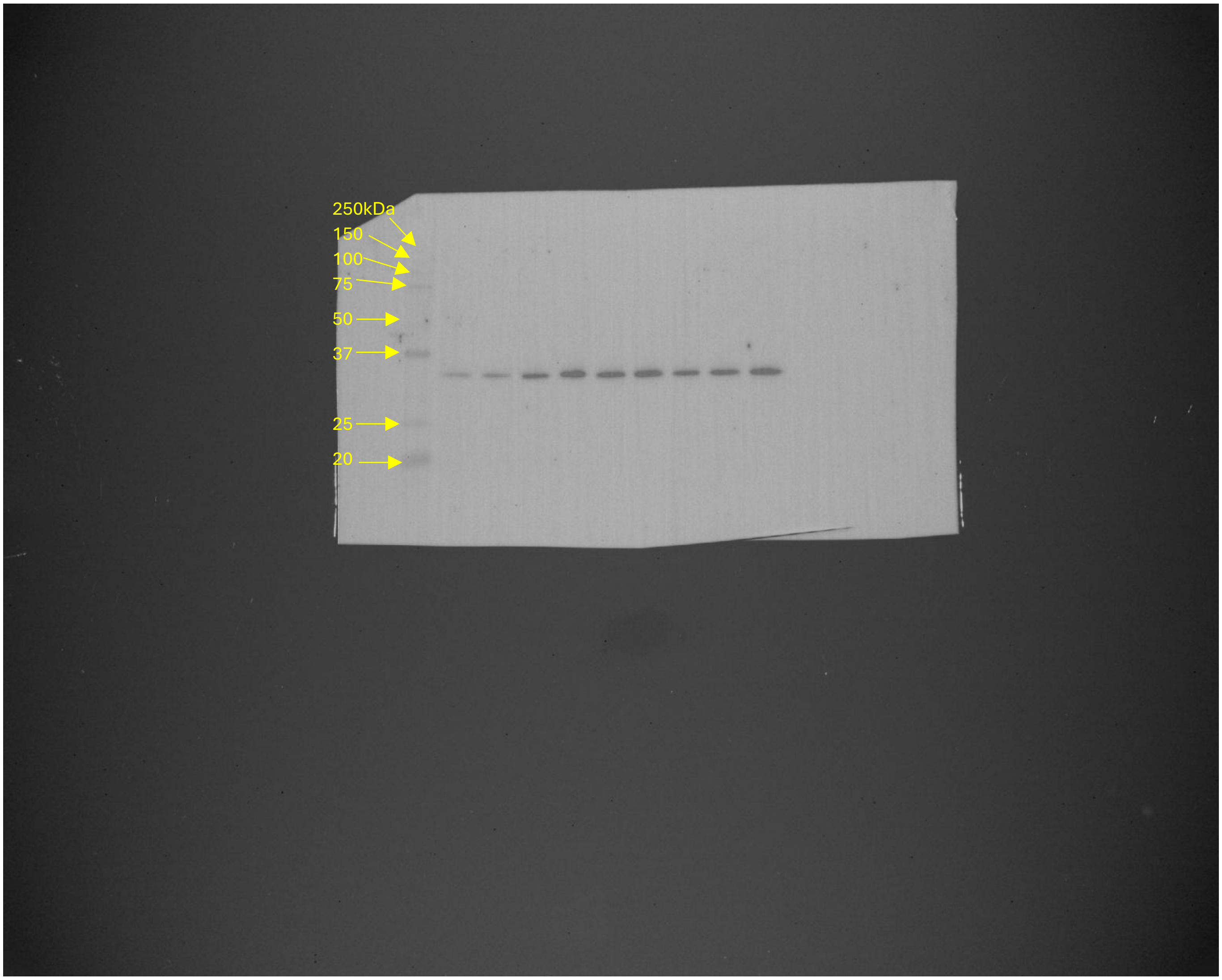

PANC-1 Western Blot Merged Uncropped Image of Membrane 2 for rps6

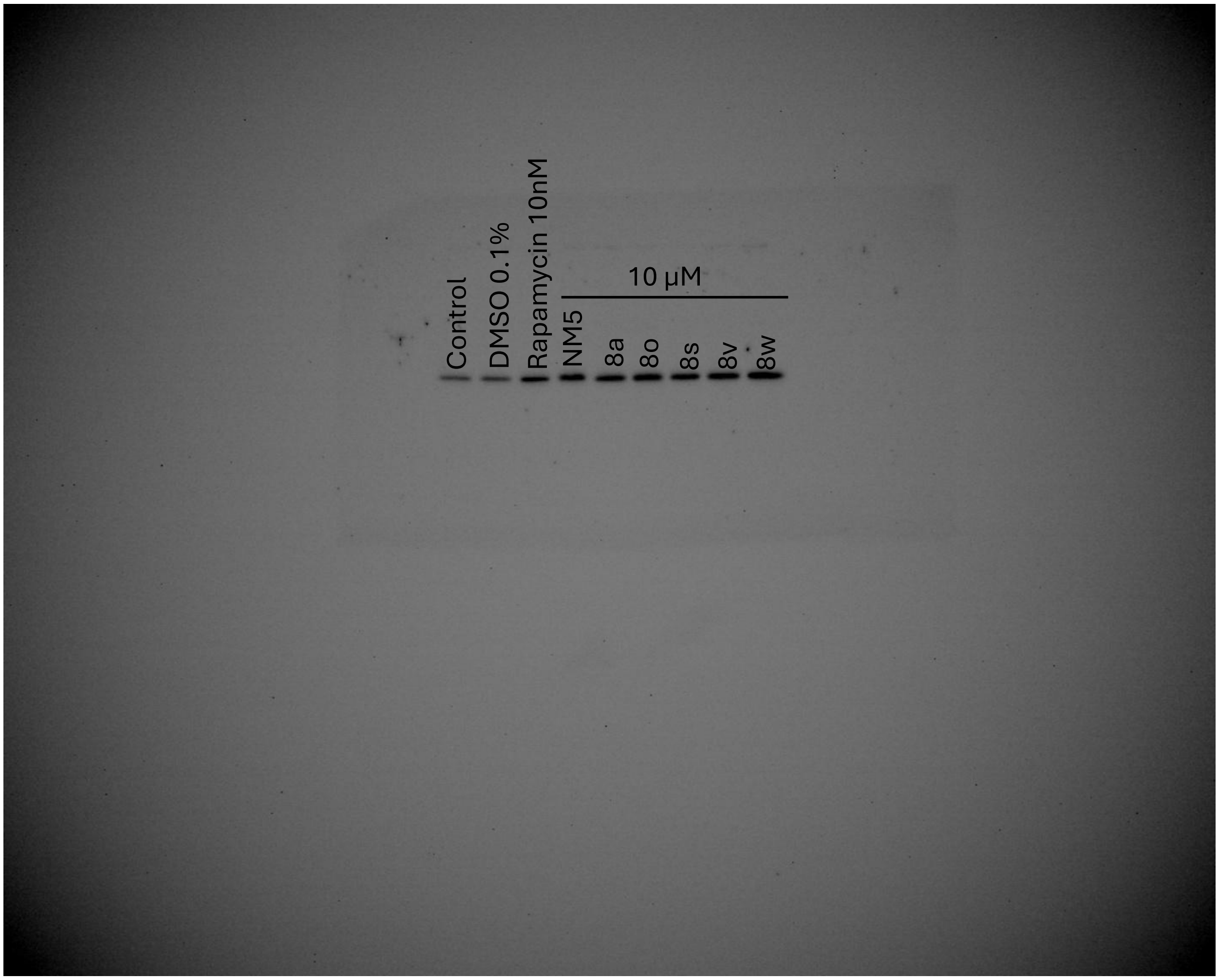

PANC-1 Western Blot Uncropped Image of Membrane 2 for rps6

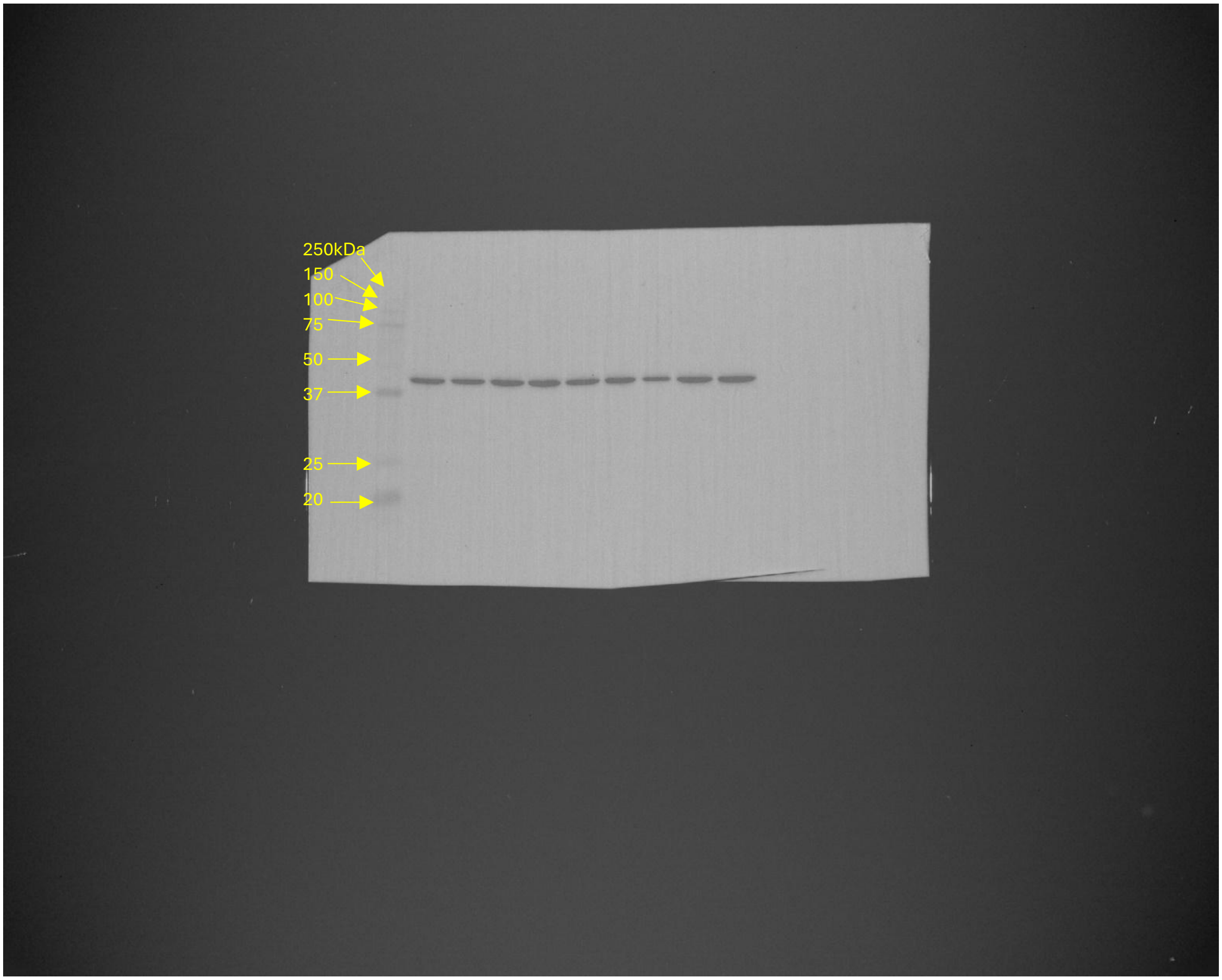

PANC-1 Western Blot Merged Uncropped Image of Membrane 2 for β-actin

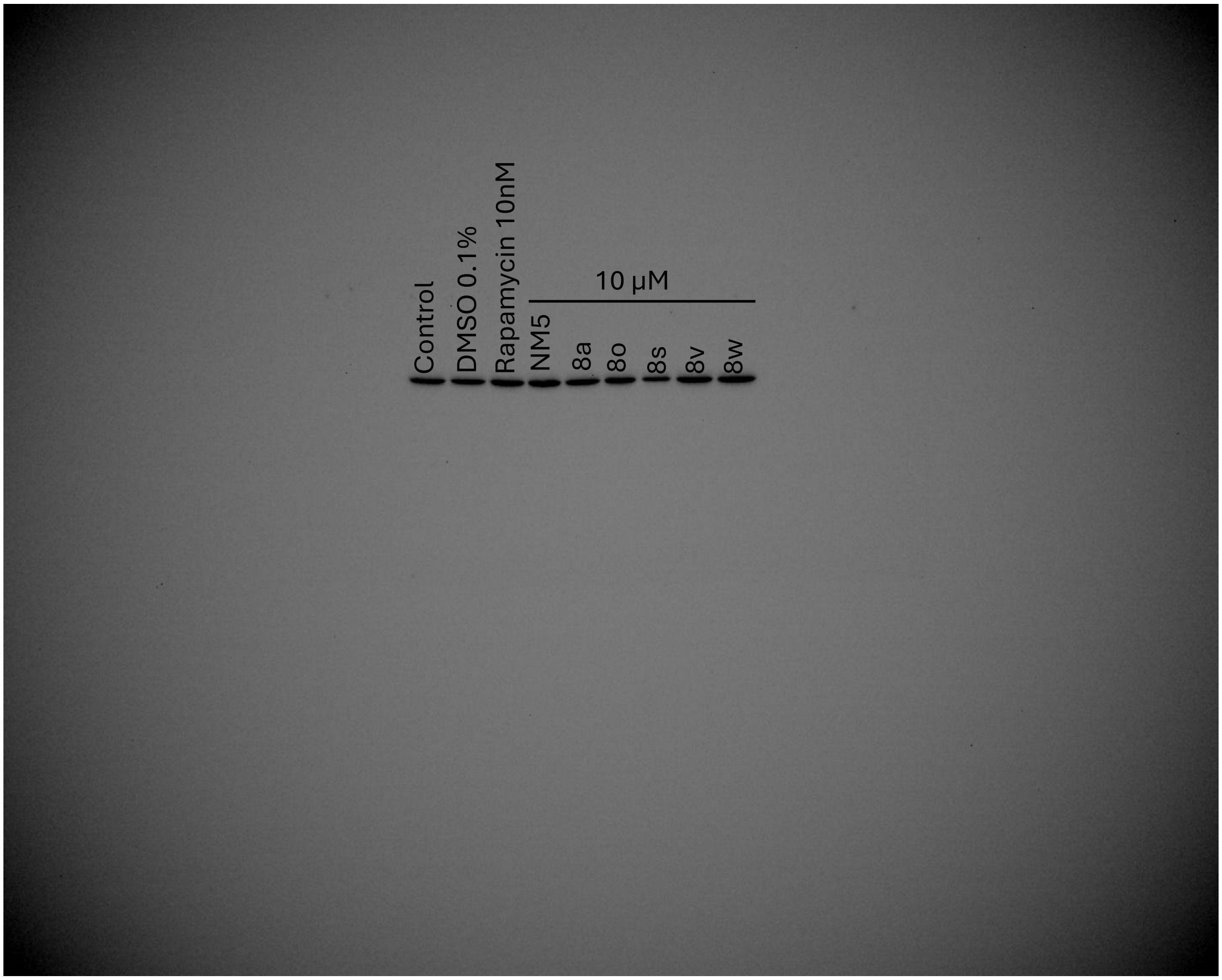

PANC-1 Western Blot Uncropped Image of Membrane 2 for β-actin
